# Silylated Stannanes and Stannides

**DOI:** 10.1021/acs.inorgchem.6c00724

**Published:** 2026-04-22

**Authors:** Roland C. Fischer, Christoph Marschner

**Affiliations:** Institut für Anorganische Chemie, Technische Universität Graz, Stremayrgasse 9, 8010 Graz, Austria

## Abstract

Tris­(trimethylsilyl)­stannyl potassium can be prepared by reaction of tetrakis­(trimethylsilyl)­stannane with ^*t*^BuOK. The compound allows for the easy introduction of a trisilylated stannyl ligand. To vary its steric and electronic properties, the replacement of trimethylsilyl for bulkier triorganosilyl groups or methyl or phenyl groups is possible. As long as the thus obtained stannanes still bear trimethylsilyl groups, they can be further converted to potassium stannides by reaction with ^*t*^BuOK. The reactions of 6-fold trimethylsilylated distannanes and silastannanes with ^*t*^BuOK permit access to even bulkier stannides and further to 1,2-dimetalated compounds. NMR spectroscopic and crystallographic characterization of the stannides provide insights into their electronic and structural properties.

## Graphical abstract

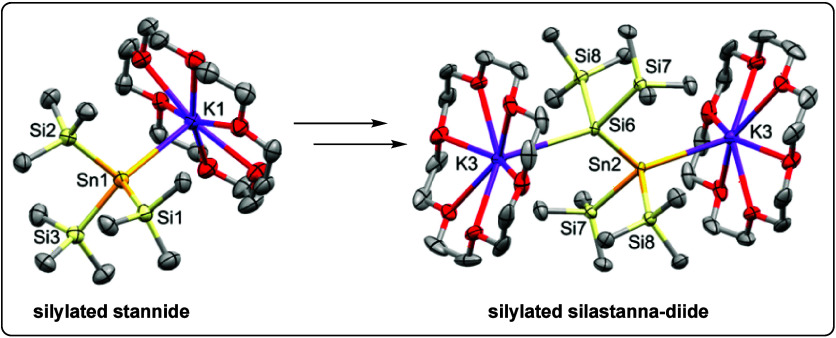

## Introduction

Anionic triorganosilyl substituted derivatives of carbon,^1^ silicon,^2,3^ and germanium^4,5^ have proven to be outstanding reagents for the synthesis of almost countless main group and transition metal compounds.^6^ Nevertheless, the chemistry of the respective tin compounds (R_3_Si)_3_SnM is by far less developed. It is interesting to point out that the synthesis of (Me_3_Si)_3_SiLi from (Me_3_Si)_4_Si and MeLi by Gilman and co-workers^7^ was reported almost simultaneous with the preparation of (Me_3_Si)_4_Si.^8^ Analogous syntheses of (Me_3_Si)_3_GeLi by Brook et al. 1986 ^4^ and in particular (Me_3_Si)_3_SnLi by Preuss et al. 1992 ^9^ were reported long after the initial preparation of (Me_3_Si)_4_Ge and (Me_3_Si)_4_Sn by Bürger and Goetze in 1968.^10^ Later Cardin et al. reported a one-pot synthesis and the first solid state structure of (Me_3_Si)_3_SnLi­(THF)_3_ from Me_3_SiCl, Li and SnCl_4_ in moderate to reasonable yields.^11,12^ Nanjo and Mochida could show that (Me_3_Si)_3_SnLi­(THF)_3_ can be distilled in vacuum to give the mono-THF adduct of the dimeric compound [(Me_3_Si)_3_SnLi]_2_.^13^

An alternative approach to the sterically more demanding trisilylated stannides (^*t*^Bu_2_MeSi)_3_SnLi and (^*t*^Bu_2_MeSi)_3_SnK was reported by Sekiguchi and co-workers, who reduced the stable trisilylated tin radical (^*t*^Bu_2_MeSi)_3_Sn^•^,^14^ with lithium and potassium.^15^ Apart from these mentioned triorganosilyl stannides, also the preparation of the even bulkier tris­[tris­(trimethylsilyl)­silyl]­stannides was reported.^16–18^

Our own approach to a series of alkali metal derivatives (Me_3_Si)_3_SnM (M = Li, Na, K, Rb, Cs) of the tris­(trimethylsilyl)­stannyl moiety extended our previous syntheses of (Me_3_Si)_3_SiK,^19,20^ (Me_3_Si)_3_GeK,^5^ and (Me_3_Ge)_3_GeK^21^ by reaction of potassium *tert*-butoxide with (Me_3_E’)_4_E (E = Si, Ge, Sn; E’ = Si, Ge) to tin.^22^ As shown in that account, NMR spectroscopic properties of these compounds strongly depend on the nature of the cation and the solvent system used.^29^Si and ^119^Sn NMR spectroscopy thus proved superb tools for the study of even subtle electronic effects as both, chemical shifts and in particular ^1^ *J*_29Si‑117/119Sn_ coupling constants are highly sensitive toward small changes in structure and bonding. Hence, we believe that silylated stannyl groups should be excellent ligands for probing electronic properties of novel main group and transition metal compounds. This was nicely demonstrated recently by Sarazin and co-workers in their study on metal–metal bonded ytterbium and alkaline-earth distannyls.^23,24^

Nevertheless, for a versatile approach to the synthesis and isolation of stannyl substituted main group and transition metal complexes, it is critical to be able to exactly adjust steric demand and electronic properties of the silylated stannyl ligands.

Although tris­(trimethylsilyl)­stannyl lithium as an entry into the chemistry of triorganosilylstannides can be prepared by other methods,^9,11,12^ we found the cleavage of tin – silicon bonds with potassium *tert*-butoxide to be the most convenient synthetic route.^22^ The reaction was found to be highly regioselective and in particular, it allows also for the preparation of more complex stannides. Herein, we thus present a systematic approach to adjust the steric demand and the electronic properties of triorganosilyl substituted potassium stannides. Moreover, selective single or double metalation of hexakis­(trimethylsilyl) substituted distannanes and silastannanes can be used to generate a higher class of stannyl mono- and dianions.

The chemistry of silylated stannides is strongly connected to silylated stannanes, as the former are most useful building blocks for the latter. As the number of acyclic neutral tris- and tetrasilylated stannanes is still rather small,^25,26^ further development of silylated stannide chemistry seems warranted.

## Results and Discussion

### Synthesis

Reaction of tetrakis­(trimethylsilyl)­stannane^10,27^ with one equivalent of potassium *tert*-butoxide and 18-crown-6 in benzene or toluene provides a straightforward approach to the respective potassium stannide **1** (Scheme 1).^22^ Although, metalation with ^*t*^BuOK can also be achieved in ethereal solvents like THF or DME, cleaner results in subsequent derivatization steps with electrophiles and an increased stability of the stannyl potassium species clearly favor the use of aromatic solvents.

**Scheme 1:**
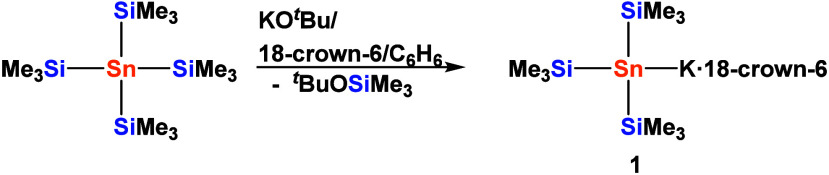
Synthesis of Tris­(trimethylsilyl)­stannyl Potassium **1**

Single crystal XRD analysis of **1** (Figure 1) caused some challenge as there seems to be some phase transition of the crystals at a temperature above 100 K.

**Figure 1:**
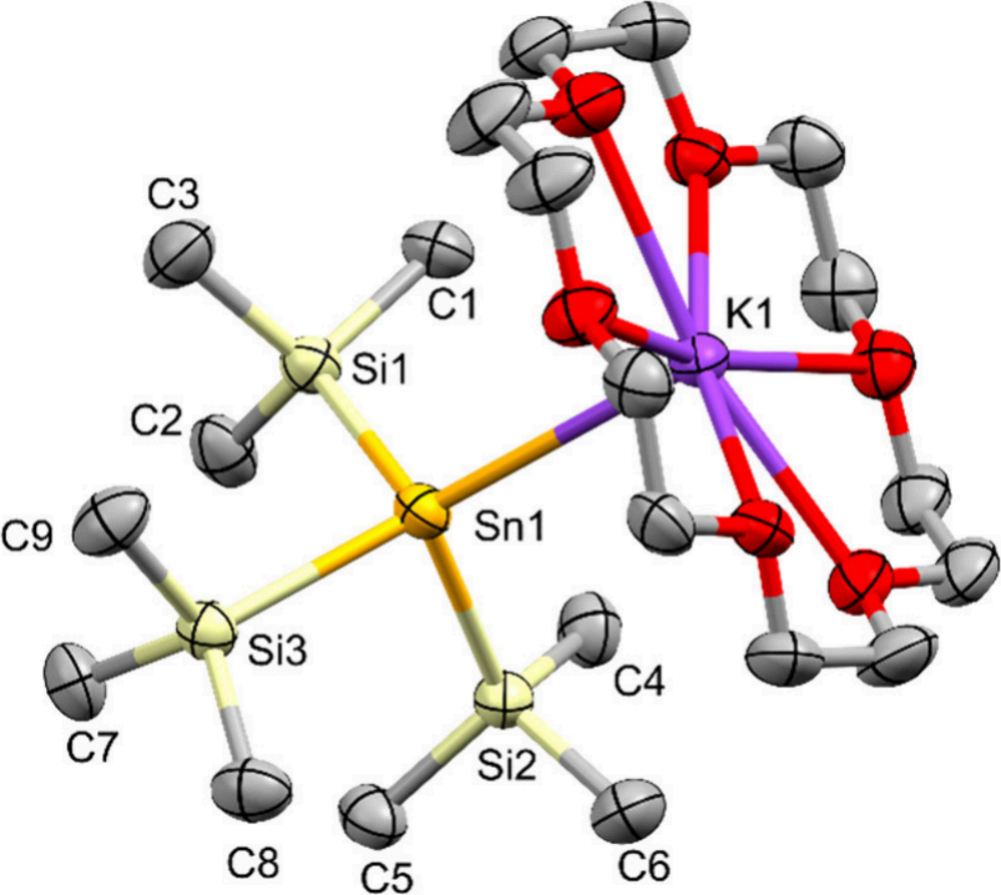
Molecular structure of tris­(trimethylsilyl)­stannyl potassium **1** in the solid state (thermal ellipsoid plot drawn at the 50% probability level). Only one of four independent molecules in the asymmetric unit is depicted. All hydrogen atoms are omitted for clarity (bond lengths in Å, angles in deg). Sn(1)–Si(1) 2.5929(14), Sn(1)–Si(2) 2.6010(13), Sn(1)–Si(3) 2.5891(13), Sn(1)–K(1) 3.6463(11), Si(1)–Sn(1)–Si(2) 96.65(4), Si(1)–Sn(1)–Si(3) 96.30(4), Si(2)–Sn(1)–Si(3) 96.32(4).

Stannide **1** is an excellent starting point for the synthesis of more complex organosilylstannyl compounds. Reactions with *tert*-butyldimethylchlorosilane, triisopropylchlorosilane, and triisopropylchlorostannane led to the respective silyl- and stannyl derivatives **2**, **3**, and **4** in high yields. Clean methylation to yield **5** can be achieved by reacting **1** with either dimethyl sulfate or with methyl iodide at low temperatures (−30 °C) (Scheme 2).

**Scheme 2:**
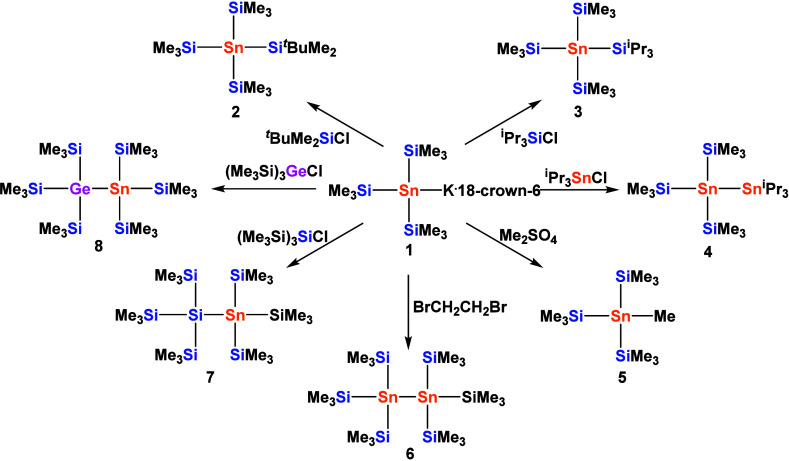
Derivatization Reactions of Tris­(trimethylsilyl)­stannyl Potassium **1**

Attempts to obtain **5** from the reaction of **1** with methyl iodide at ambient temperature resulted in the formation of hexakis­(trimethylsilyl)­distannane **6** as a side product, presumably as a consequence of initial metal halogen exchange reaction. Oxidative coupling of **1** with 1,2-dibromoethane gave hexakis­(trimethylsilyl)­distannane **6** ^28,29^ in almost quantitative yield.

Furthermore, reactions of **1** with tris­(trimethylsilyl)­chlorosilane^30^ and tris­(trimethylsilyl)­chlorogermane^4^ provided access to the hexakis­(trimethylsilyl) substituted mixed silicon–tin and germanium–tin homologues **7** and **8** (Scheme 2).^29^ However, all attempts to obtain (Me_3_Si)_3_SnH failed. Although the addition of various proton sources to solutions of **1** caused the yellow color of the solution to vanish quickly, after a short period of induction, gas evolution was observed and elemental tin deposited on the walls of the reaction vessel.

Reaction of methylated stannane **5** with potassium *tert*-butoxide/18-crown-6 provided access to methylbis­(trimethylsilyl)­stannyl potassium **9**. Derivatization of **9** with dimethyl sulfate afforded the previously reported dimethylbis­(trimethylsilyl)­stannane **10** (Scheme 3).^31,32^ The latter can be converted to (Me_3_Si)­Me_2_SnK,^31^ so that the whole series of methylated and trimethylsilylated potassium stannides ((Me_3_Si)_3–n_Me_n_SnK is now synthetically accessible (see Table 1 for a comparison of NMR spectroscopic properties).

**Scheme 3:**
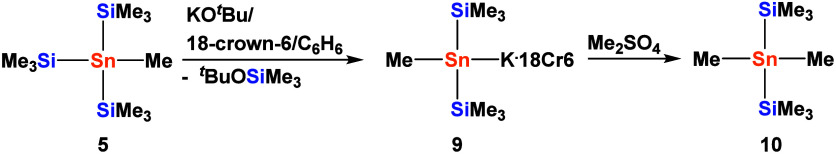
Formation and Derivatization of Methylbis­(trimethylsilyl)­stannyl Potassium **9**

**Table 1:** Trends in ^119^Sn and ^29^Si NMR Chemical Shifts and ^119^Sn–^29^Si Scalar Couplings of Neutral and Anionic Methylated, Phenylated, and Trimethylsilylated Stannanes^a^

compound	δ^119^Sn [ppm]	δ^29^Si [ppm]	^1^ *J*_29Si‑119Sn_ [Hz]	ref.
(Me_3_Si)_4_Sn	–664.4	–9.7	351	^22^
(Me_3_Si)_3_SnK (**1**)	–890.3	–12.6	281	^22^
(Me_3_Si)_3_SnMe (**5**)	–457.4	–10.1	423	this work
(Me_3_Si)_2_MeSnK (**9**)	–439.1	–5.6	347	this work
(Me_3_Si)_2_SnMe_2_	–274.4	–10.4	525	^32^
(Me_3_Si)Me_2_SnK	–346.8	–8.4	456	^31^
Me_3_SiSnMe_3_	–126.7	–11.0	656	^44^,^45^
Me_3_SnK	–168.0	n.a.	n.a.	^46^
Me_4_Sn	0	n.a.	n.a.	^45^,^47^
(Me_3_Si)_2_SnPh_2_	–255.2	–7.0	520	^31^
(Me_3_Si)Ph_2_SnK (**11**)	–227.0	–9.7	402	^31^

a All anionic compounds chosen have 18-crown-6 ether potassium as counter ions.

In a related way, it was possible to obtain (Me_3_Si)­Ph_2_SnK (**11**) from the reaction of (Me_3_Si)_2_SnPh_2_ ^32^ with ^*t*^BuOK in the presence of crown ether (Scheme 4).

**Scheme 4:**
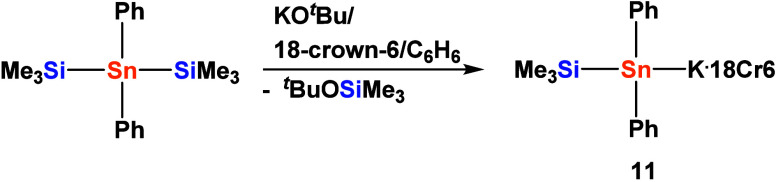
Formation of Diphenyl­(trimethylsilyl)­stannyl Potassium **11**

The 18-crown-6 adducts of (Me_3_Si)­Me_2_SnK and (Me_3_Si)­Ph_2_SnK (**11**) were originally reported by the reactions of (Me_3_Si)_2_SnR_2_ (R = Me, Ph) with KH in the presence of 18-crown-6.^31^ While this is a valuable reaction, the easier handling of ^*t*^BuOK compared to KH makes the synthetic method using the alkoxide preferable. Single crystal XRD analysis of **11** (Figure 2) again showed a typical structure.

**Figure 2:**
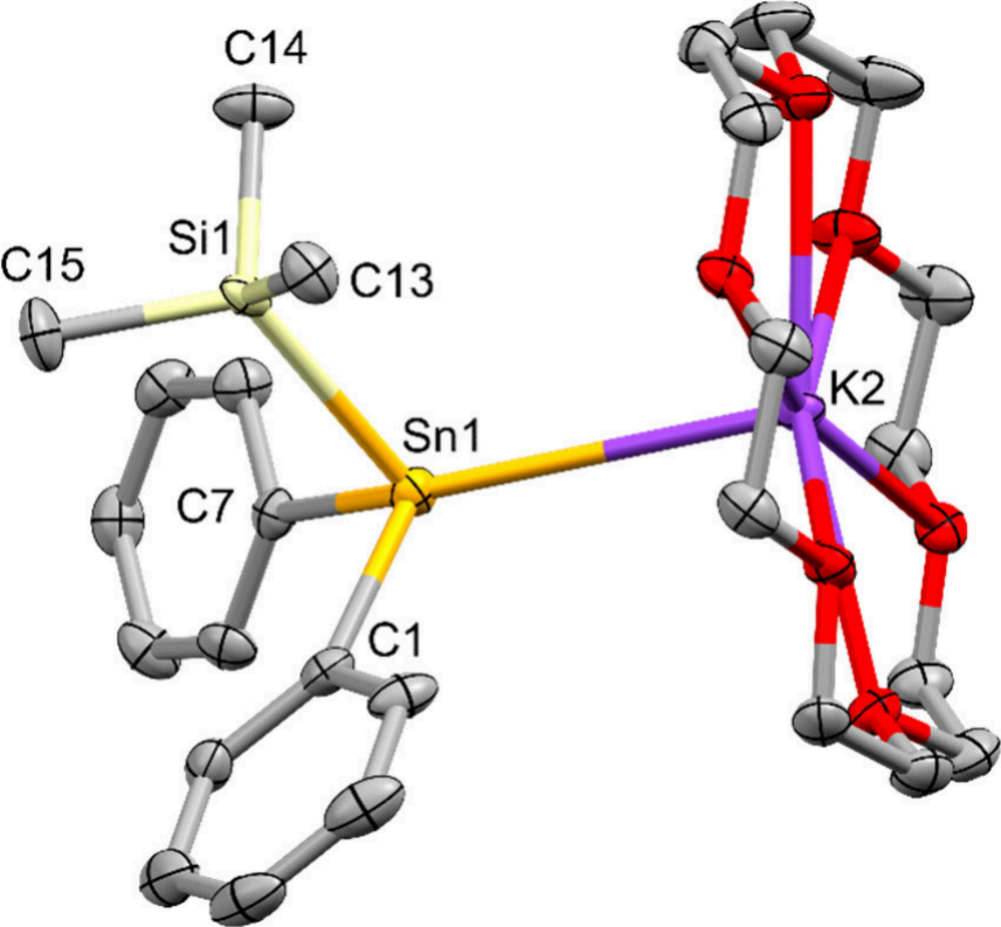
Molecular structure of diphenyl­(trimethylsilyl)­stannyl potassium **11** in the solid state (thermal ellipsoid plot drawn at the 50% probability level). All hydrogen atoms are omitted for clarity (bond lengths in Å, angles in deg). Sn(1)–Si(1) 2.6286(11), Sn(1)–C(1) 2.229(4), Sn(1)–C(7) 2.224(4), Sn(1)–K(2) 3.7366(9), Si(1)–Sn(1)–C(1) 92.15(10), Si(1)–Sn(1)–C(7) 95.56(10), C(1)–Sn(1)–C(7) 101.78(14).

The cleavage of trimethylsilyl–tin bonds with potassium *tert-*butoxide is highly regioselective as demonstrated by the clean formation of anions **12** (see Figure 3 for the molecular structure of **12** in the solid state) and **13**, starting from **2** and **3**, respectively (Schemes 5 and 6).

**Scheme 5:**
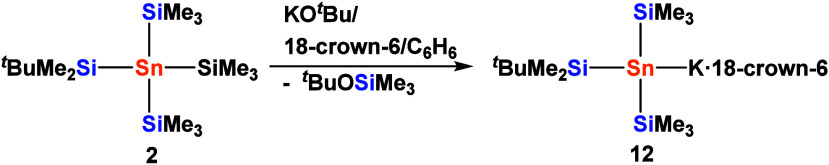
Formation of *tert*-Butyldimethylsilylbis­(trimethylsilyl)­stannyl Potassium **12**

**Figure 3:**
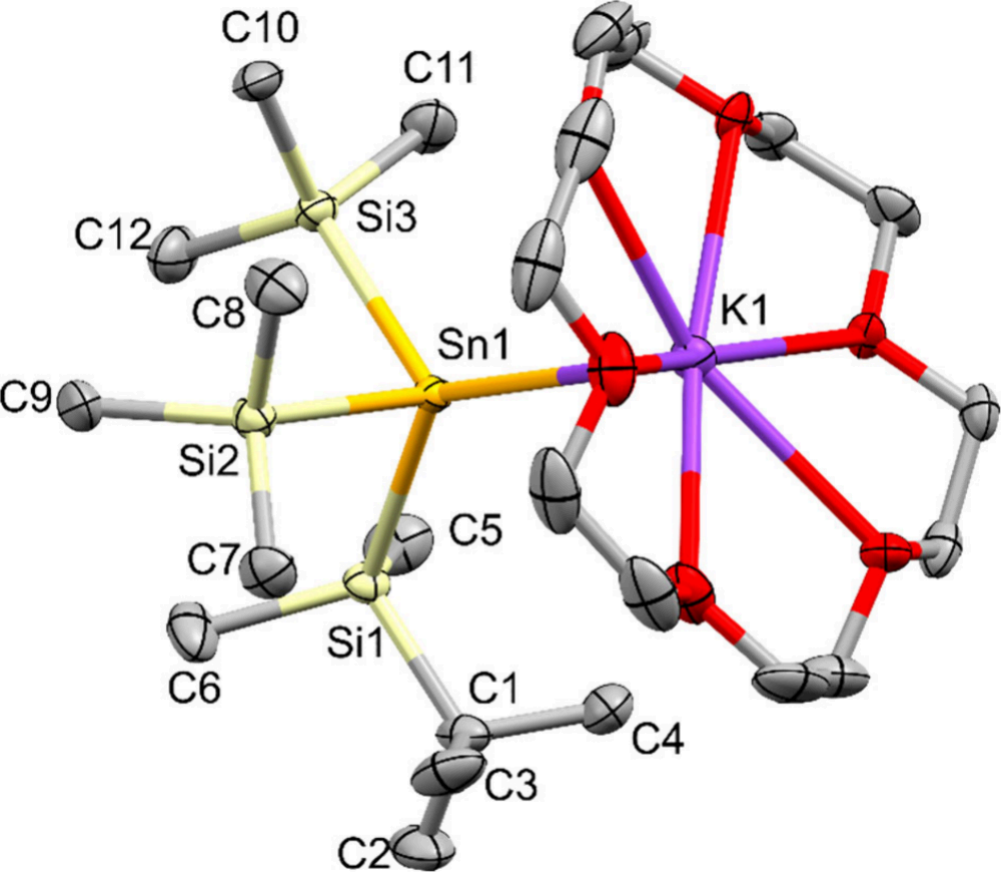
Molecular structure of *tert*-butyldimethylsilylbis­(trimethylsilyl)­stannyl potassium **12** in the solid state (thermal ellipsoid plot drawn at the 50% probability level). All hydrogen atoms are omitted for clarity (bond lengths in Å, angles in deg). Sn(1)–Si(1) 2.6006(7), Sn(1)–Si(2) 2.5993(7), Sn(1)–Si(3) 2.6102(7), Sn(1)–K(1) 3.7083(6), Si(1)–Sn(1)–Si(2) 98.11(2), Si(1)–Sn(1)–Si(3) 98.03(2), Si(2)–Sn(1)–Si(3) 94.05(2).

**Scheme 6:**
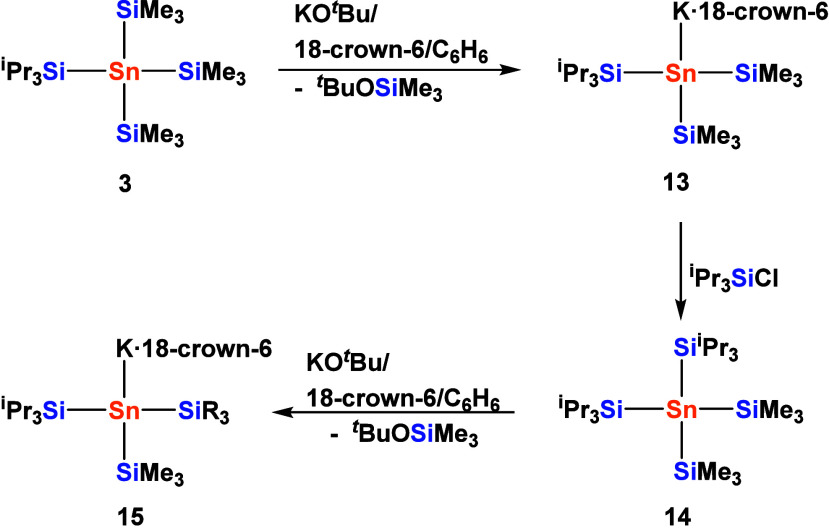
Stepwise Formation of Potassium Stannides **13** and **15** with One and Two Bulky Triisopropylsilyl Substituents, Respectively

This allows for the directed synthesis of silyl substituted stannyl anions with adjustable steric demand. A repeated stepwise silylation/cleavage procedure provides access to the bulky tetrasilylated stannane **14** and the respective stannide **15** (see Figure 4 for the molecular structure of **15** in the solid state), bearing two triisopropylsilyl groups (Scheme 6).

**Figure 4:**
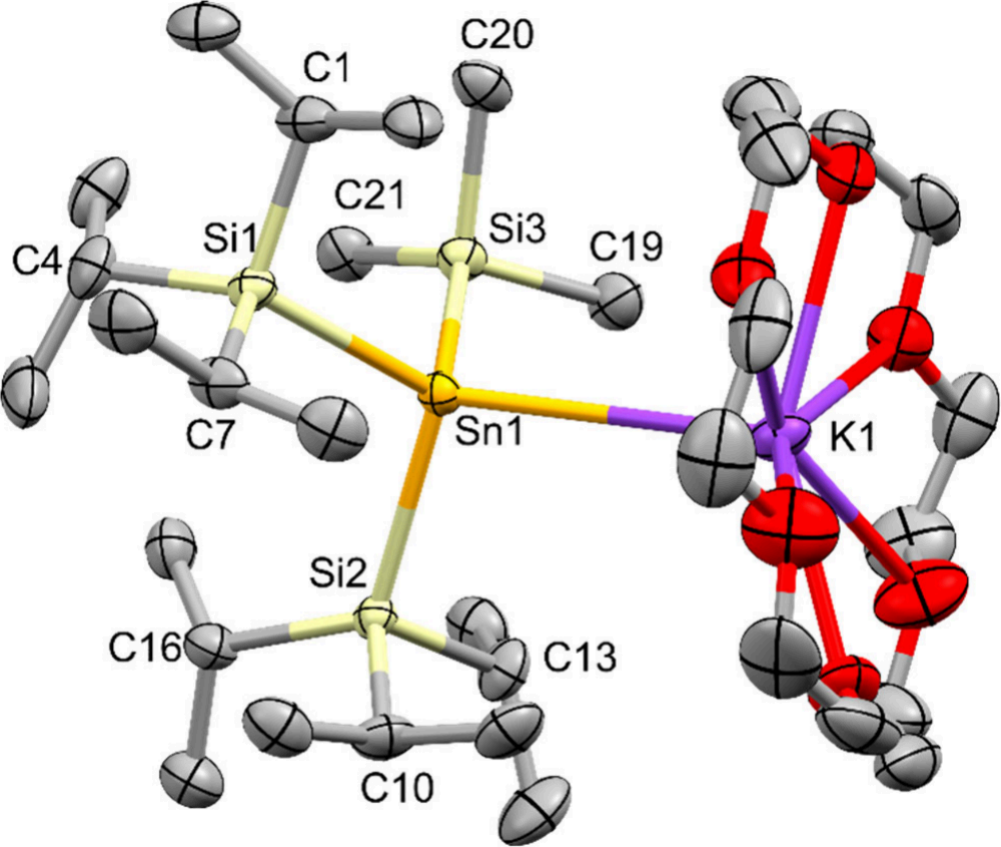
Molecular structure of bis­(triisopropylsilyl)­(trimethylsilyl)­stannyl potassium **15** in the solid state (thermal ellipsoid plot drawn at the 50% probability level). All hydrogen atoms are omitted for clarity (bond lengths in Å, angles in deg). Sn(1)–Si(1) 2.6335(17), Sn(1)–Si(2) 2.614(2), Sn(1)–Si(3) 2.6125(19), Sn(1)–K(1) 3.7866(16), Si(1)–Sn(1)–Si(2) 110.33(6), Si(1)–Sn(1)–Si(3) 104.83(7), Si(2)–Sn(1)–Si(3) 104.28(7).

The reactions of the 6-fold trimethylsilylated compounds **6** and **7** with one equivalent of potassium *tert*-butoxide/18-crown-6 yielded the respective monoanions **16** and **17** (see Figures 5 and 6 for their molecular structures in the solid state) as the only products (Scheme 7). In the case of **7**, the metalation was found to occur only at the tin center and by multinuclear NMR spectroscopy, no silyl anion could be detected in the reaction mixture.

**Scheme 7:**
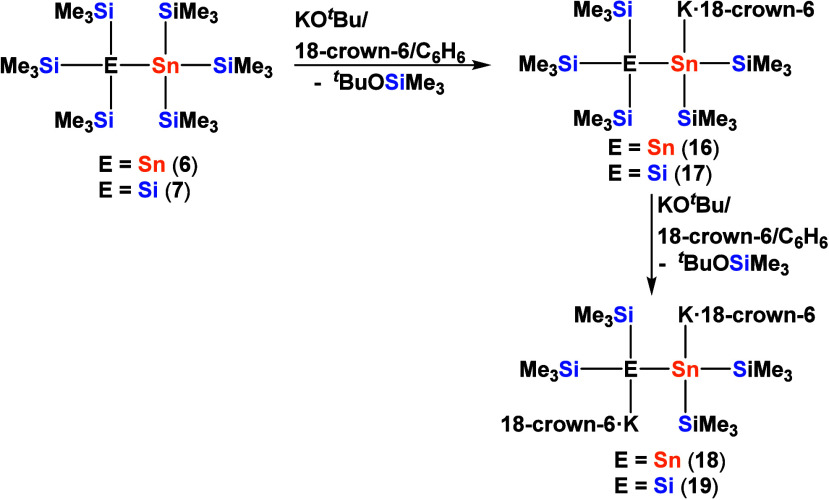
Selective Mono- and Dimetalations of the 6-Fold Trimethylsilylated Compounds **6** and **7**

**Figure 5:**
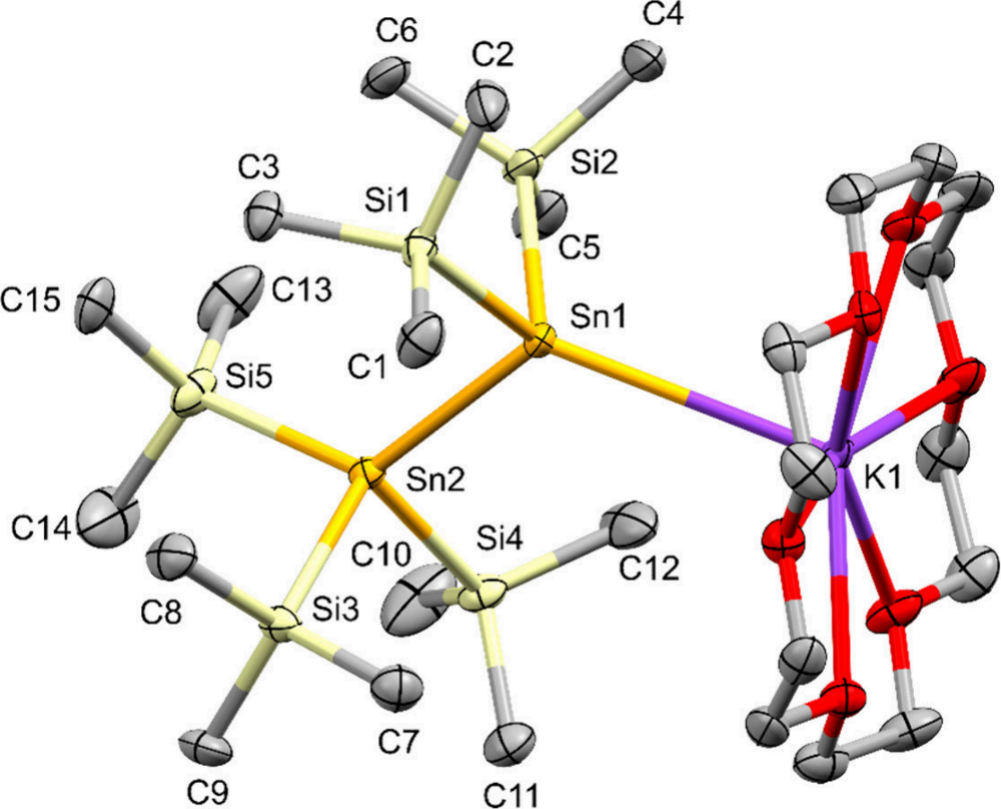
Molecular structure of pentakis­(trimethylsilyl)­distannyl potassium **16** in the solid state (thermal ellipsoid plot drawn at the 50% probability level). Only one of five independent molecules in then asymmetric unit is depicted. All hydrogen atoms are omitted for clarity (bond lengths in Å, angles in deg). Sn(1)–Si(1) 2.6057(19), Sn(1)–Si(2) 2.603(2), Sn(1)–Sn(2) 2.8382(6), Sn(1)–K(1) 3.6844(14), Sn(2)–Si(3) 2.5823(18), Sn(2)–Si(4) 2.589(2), Sn(2)–Si(5) 2.572(2), Si(1)–Sn(1)–Si(2) 95.69(6), Si(1)–Sn(1)–Sn(2) 100.08(4), Si(2)–Sn(1)–Sn(2) 94.72(5), Si(3)–Sn(2)–Si(4) 104.33(6), Si(3)–Sn(2)–Si(5) 104.43­(7), Si(4)–Sn(2)–Si(5) 105.53(7), K(1)–Sn(1)–Sn(2)-Si(5) 147.57(7).

**Figure 6:**
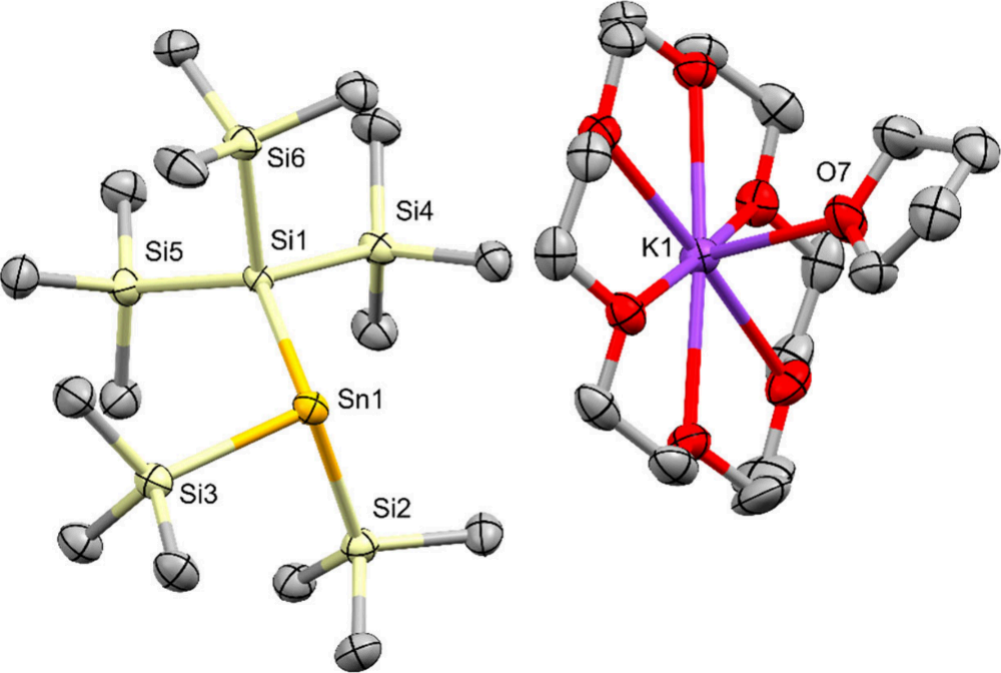
Molecular structure of pentakis­(trimethylsilyl)-2-siladistannyl potassium **17** in the solid state (thermal ellipsoid plot drawn at the 50% probability level). All hydrogen atoms are omitted for clarity (bond lengths in Å, angles in deg). Sn(1)–Si(1) 2.6263(6), Sn(1)–Si(2) 2.6184(7), Sn(1)–Si(3) 2.6124(7), Sn(1)–K(1) 6.1783(8), Si1(2)–Si(4) 2.3506(8), Si1(2)–Si(5) 2.3509(8), Si1(2)–Si(6) 2.3668(8), K(1)–O(7) 2.673(6), Si(1)–Sn(1)–Si(2) 105.254(19), Si(1)–Sn(1)–Si(3) 102.074(19), Si(2)–Sn(1)–Si(3) 94.65(2), Si(4)–Si(1)–Si(5) 104.44(3), Si(4)–Si(1)–Si(6) 105.61(3), Si(5)–Si(1)–Si(6) 105.96­(3), Si(5)–Si(1)–Sn(1) 123.78(3).

However, addition of a second equivalent of metalating agent produces the respective dianions **18** ^33^ (see Figure 7 for the molecular structure of **18** in the solid state) and **19** as the sole products. Although a couple of 1,2-dianionic distannanes^31,34–39^ have been reported, only the two examples of bistannole-1,2-dianions^38,39^ have been structurally characterized. Therefore, to the best of our knowledge, **18** represent the first fully structurally characterized examples of a silylated 1,2-dimetalated distannane and **19** is the first reported 1,2-dianionic silastannane (Scheme 7).

**Figure 7:**
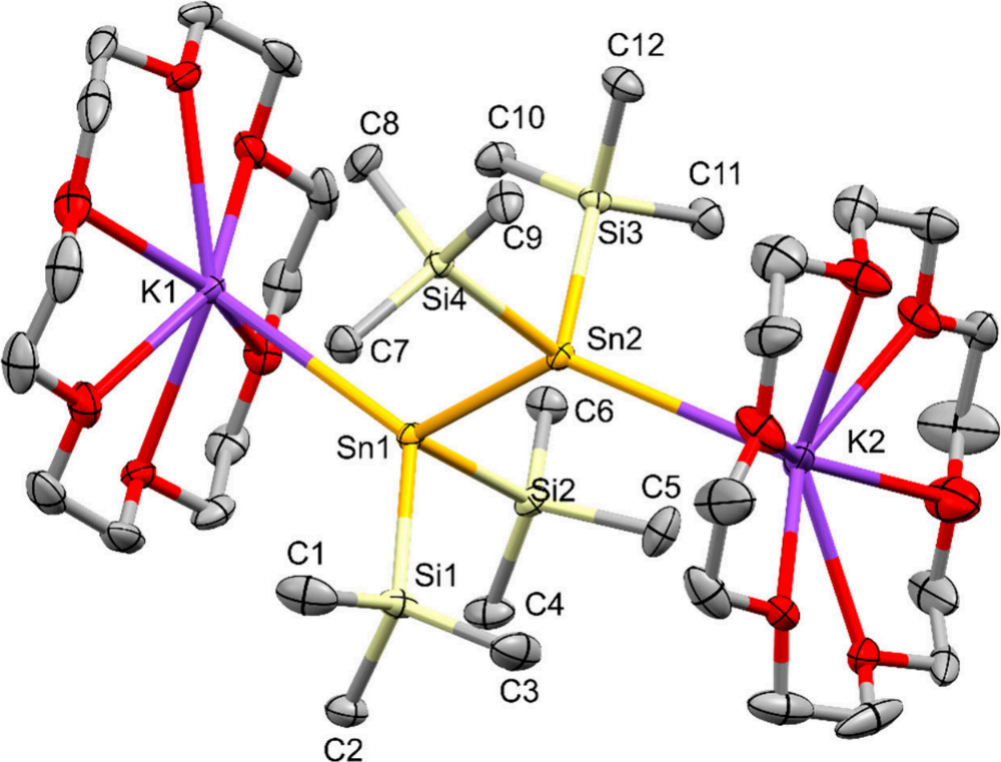
Molecular structure of tetrakis­(trimethylsilyl)­distanna-1,2-diyl dipotassium **18** in the solid state (thermal ellipsoid plot drawn at the 50% probability level). Another half molecule of **18** on a special position, two cocrystallized benzene molecules and all hydrogen atoms are omitted for clarity (bond lengths in Å, angles in deg). Sn(1)–Si(1) 2.6071(5), Sn(1)–Si(2) 2.6089(5), Sn(1)–Sn(2) 2.9038(2), Sn(1)–K(1) 3.5887(4), Sn(2)–Si(3) 2.6098(5), Sn(2)–Si(4) 2.6004(5), Sn(2)–K(2) 3.6006(11), Si(1)–Sn(1)–Si(2) 94.75(2), Si(1)–Sn(1)–Sn(2) 95.20(2), Si(2)–Sn(1)–Sn(2) 95.90(2), Si(3)–Sn(2)–Si(4) 92.31(2), Si(3)–Sn(2)–Sn(1) 95.39(2), Si(4)–Sn(2)–Sn(1) 96.10(2).

### NMR Spectroscopy

^29^Si and ^119^Sn NMR chemical shifts as well as scalar coupling constants ^1^ *J*_29Si‑117/119Sn_ are well suited for probing electronic and bonding properties in silylated stannyl compounds.^40^

Compared to the respective neutral compounds, the chemical shift behavior and ^1^ *J*_Sn–Si_ coupling constants of fully^12,22^ and partially silylated stannides resemble what is already known for silylated silanides.^41^ Exchange of a trimethylsilyl group from (Me_3_Si)_3_Sn­(SiR_3_) by an alkali metal at the central tin atom induces an upfield shift of some 200 ppm in the ^119^Sn NMR spectra (Tables 1 and 2). In addition, also the resonances for the silicon atoms attached to the central tin atom are shifted slightly to higher field. Replacement of a trimethylsilyl substituent by a methyl group causes a downfield shift in ^119^Sn NMR spectra of more than 200 ppm (Table 1). To obtain complete sets of all members of the series (Me_3_Si)_4–*n*_SnMe_*n*_ and (Me_3_Si)_3–*n*_Me_*n*_SnK, the novel compounds **5** and **9** were synthesized and their spectroscopic data, compared to literature data (Table 1). The chemical shift trends observed for (Me_3_Si)_4_Sn and (Me_3_Si)_3_SnK hold for all members of the series but the degree of the chemical shift change becomes smaller with increased methylation character of the compounds (Table 1). The upfield shift caused by the metalation of Me_3_SiSnMe_3_ eventually amounts only to some 40 ppm. But the downfield shift caused by the exchange of the final trimethylsilyl group of Me_3_SiSnMe_3_ to obtain Me_4_Sn is still 126.7 ppm. While ^119^Sn resonances are experiencing a downfield shift upon successive methylation, the ^1^ *J*_29Si‑119Sn_ coupling constants are rising from 381 Hz for (Me_3_Si)_4_Sn to 656 Hz for Me_3_SiSnMe_3_ (Table 1). This behavior likely is caused by a stronger sp^3^ character of the Sn-Me bonds, which in turn raises the s-character of the Sn–Si bonds. The same trend of increasing ^1^ *J*_29Si‑119Sn_ coupling constants for higher methylated compounds is also observed for the (Me_3_Si)_3–*n*_Me_*n*_SnK stannide series (Table 1). The involved values here rise from 281 Hz for (Me_3_Si)_3_SnK to 456 Hz for (Me_3_Si)­Me_2_SnK (Table 1).

**Table 2:** ^119^Sn and ^29^Si NMR Chemical Shifts and ^119^Sn–^29^Si Scalar Coupling Constants of Neutral and Anionic Stannanes with Trimethylsilyl and More Bulky Silyl Groups

	δ^119^Sn	δ^29^Si Me_3_Si	δ^29^Si R_3_Si	^1^ *J*_29Si‑119Sn_ Me_3_Si–Sn	^1^ *J*_29Si‑119Sn_ R_3_Si–Sn	ref
	[ppm]	[ppm]	[ppm]	[Hz]	[Hz]	
(Me_3_Si)_4_Sn	–664.4	–9.7	n.a.	351	n.a.	^22^
(MeSi_3_)_3_SnK (**1**)	–892.2	–12.7	n.a.	271	n.a.	^22^
(*^t^* BuMe_2_Si)(Me_3_Si)_3_Sn (**2**)	–673.1	–10.3	10.3	338	343	this work
(*^i^* Pr_3_Si)(Me_3_Si)_3_Sn (**3**)	–685.0	–11.1	29.3	323	337	this work
(*^i^* Pr_3_Sn)(Me_3_Si)_3_Sn (**4**)	–701.7/ –10.7(SnMe_3_)	–8.7	n.a.	325	n.a.	this work
[(Me_3_Si)_3_Sn]_2_ (**6**)	–677.8	–10.0	n.a.	320	n.a.	this work
(Me_3_Si)_3_SnSi(SiMe_3_)_3_ (**7**)	–627.3	–10.3	–135.4	324	314	this work
(Me_3_Si)_3_SnGe(SiMe_3_)_3_ (**8**)	–609.0	–10.4	n.a.	321	n.a.	this work
(*^t^* BuMe_2_Si)(Me_3_Si)_2_SnK (**12**)	–908.4	–13.4	5.6	278	252	this work
(*^i^* Pr_3_Si)(Me_3_Si)_2_SnK (**13**)	–908.6	–16.1	26.2	281	296	this work
(*^i^* Pr_3_Si)_2_(Me_3_Si)_2_Sn (**14**)	–673.8	–11.6	25.1	286	301	this work
(*^i^* Pr_3_Si)_2_(Me_3_Si)SnK (**15**)	–929.8	–19.4	23.8	344	363	this work
(MeSi_3_)_3_Sn(Me_3_Si)_2_SnK·18-c-6 (**16**)	–725.4/ –836.2(SnK)	–12.0/ –14.4(SnK)	n.a.	202/346(SnK)	n.a.	this work
(MeSi_3_)_3_Si(Me_3_Si)_2_SnK·18-c-6 (**17**)	–812.9	–15.1	–152.6	348	345	this work
[(MeSi_3_)_2_SnSn(SiMe_3_)_2_]K_2_(18-c-6)_2_ (**18**)	–899.1	–17.6	n.a.	416	n.a.	this work
[(MeSi_3_)_2_SnSi(SiMe_3_)_2_]K_2_(18-c-6)_2_ (**19**)	–775.1	–18.4	–205.5	348	122	this work

There is not enough data yet to do a similar analysis on the series of (Me_3_Si)_4–*n*_SnPh_*n*_ and (Me_3_Si)_3–*n*_Ph_*n*_SnK. However, the comparison of (Me_3_Si)_2_SnPh_2_ with (Me_3_Si)­Ph_2_SnK (**11**) does not show a ^119^Sn shift to higher field as observed for the (Me_3_Si)_2_SnMe_2_ to (Me_3_Si)­Me_2_SnK case, but causes even a downfield shift of some 30 ppm (Table 1). For the analogous silicon case, namely the conversion of (Me_3_Si)_2_SiPh_2_ ^42^ to (Me_3_Si)­Ph_2_SiK,^43^ a slight upfield shift of less than 2 ppm was observed.

The formal replacements of trimethylsilyl from (Me_3_Si)_4_Sn (δ^119^Sn: −664.4 ppm) against triorganosilyl groups to compounds **2** and **3**, which build up more steric strain, cause moderate ^119^Sn upfield shifts of 8.7 and 20.6 ppm, respectively, for the central tin atoms (Table 2). This behavior is in contrast to what was observed for similar neopentasilanes, where the replacements of trimethylsilyl groups from (Me_3_Si)_4_Si for sterically more demanding triorganosilyl groups usually causes a slight downfield shift of the central silicon ^29^Si resonance.^20^ Introduction of the large tris­(trimethylsilyl)­silyl and tris­(trimethylsilyl)­germyl units on the other hand shift the ^119^Sn resonance to lower field by 37.1 and 55.4 ppm, respectively.

As was already found for oligosilanes and oligosilanides, the ^29^Si NMR resonances of sterically more demanding triorganosilyl groups are shifted to lower field compared to trimethylsilyl substituents. However, while the chemical shift of SiMe_3_ groups of (Me_3_Si)_4–*n*_Si­(SiR_3_)_*n*_ and (Me_3_Si)_4–*n*_Sn­(SiR_3_)_*n*_ are very similar, the bulky SiR_3_ groups attached to a tin atom are shifted to a larger degree to lower field. The ^1^ *J*_29Si‑119Sn_ coupling of (Me_3_Si)_4_Si amounts to 351 Hz. For the cases of **2** and **3**, similar despite somewhat lower values were observed with the coupling to the bulky silyl group slightly higher than the ones to the SiMe_3_ (Table 2). For compound **14** with two SiMe_3_ and two Si^*i*^Pr_3_ groups, a ^119^Sn shift comparable to **2** and **3** was observed but the ^1^ *J*_29Si‑119Sn_ coupling constants are markedly smaller amounting only to 286 and 301 Hz, respectively.

The stannides **1**, **12** and **13** derived from (Me_3_Si)_4_Sn, **2**, and **3** display the same pattern discussed already above for the (Me_3_Si)_4–*n*_SnMe_*n*_/(Me_3_Si)_3–*n*_Me_*n*_SnK series. Upon metalation with ^*t*^BuOK the ^119^Sn resonances shift some 230 ppm to higher field (Table 2). Accordingly, the ^1^ *J*_29Si‑119Sn_ coupling constants (to both SiMe_3_ and SiR_3_ groups) are diminished by some 40 to 80 Hz, with the compounds bearing more bulky silyl groups displaying the effect to a smaller extent. Compound **15** is following the trend by shifting the ^119^Sn resonance from −673.8 ppm for **14** to higher field to a value of −929.8 ppm. However, the ^1^ *J*_29Si‑119Sn_ coupling constants now display a converse effect being increased to 344 and 363 Hz for the SiMe_3_ and Si^*i*^Pr_3_ groups. While this behavior is unexpected, we observed something related for compounds **16**, **17**, **18**, and **19**. For compound **16** derived from **6**, both ^119^Sn resonances are shifted to higher field, with the anionic tin atom resonating at −836.2 ppm. The ^1^ *J*_29Si‑119Sn_ coupling constants as observed in the ^29^Si and ^119^Sn spectra are 346 Hz at the anionic tin and 202 Hz at the neutral tin atom. Although this is unexpected, it is consistent with the values observed for compound **17**, where the ^1^ *J*_29Si‑119Sn_ coupling constant to the SiMe_3_ groups is 348 Hz. The ^119^Sn-NMR spectrum of **16** displays the interesting phenomenon of an AB coupling pattern of the ^119^Sn satellites. For this reason, the intensities of the ^119^Sn satellites are not equal any more but displays a roof effect and the doublets move away from the center of gravity whereas the ^117^Sn satellites follow the usual behavior as being symmetrical around the central signal.^48,49^

As mentioned, for compound **17** the coupling constant to the SiMe_3_ groups is 348 Hz and the coupling to the central silicon of the attached tris­(trimethylsilyl)­silyl group is very similar (345 Hz.). The ^119^Sn resonance is at −812.9 ppm and the ^29^Si resonance of the attached central Si atom experiences an upfield shift of 17 ppm to −152.6 ppm.

For the 1,2-distannane diide **18** the ^1^ *J*_29Si‑119Sn_ coupling constant to the SiMe_3_ groups increases even further to 416 Hz, while the ^119^Sn resonance at −899.1 ppm is close to what was observed for **1**, **12**, **13**, and **15**. The mixed dianion **19** exhibits again some unexpected spectroscopic features. The ^119^Sn resonance displays the lowest field shift of all stannides in this series at −775.1 ppm, whereas the ^29^Si resonance of the anionic silicon atom at −205.5 ppm is clearly shifted to somewhat higher field compared to the more usual −195 ppm for (Me_3_Si)_3_SiK.^19^ Quite striking is the discrepancy between the ^1^ *J*_29Si‑119Sn_ coupling constant to the SiMe_3_ of 348 Hz, which falls within the range of what was observed for **15**, **16**, **17**, and **18** and a ^1^ *J*_29Si‑119Sn_ coupling constant of only 122 Hz to the neighboring anionic silicon atom (Table 2).

In a crude approximation, it seems valid to associate the magnitude of the ^1^ *J*_29Si‑119Sn_ coupling constants with the s-character of the respective bonds. The diminished coupling constants of tin to the attached silyl groups in compounds **1**, **12**, **13**, and **14** compared to the neutral compounds (Me_3_Si)_4_Sn, **2**, **3**, and **4** can thus be interpreted as a higher p-character in the Sn–Si bonds. This is consistent with an increased degree of pyramidalization as described below in the discussion of the solid-state structures. The comparably larger ^1^ *J*_29Si‑119Sn_ coupling constant of stannide **15** suggests a stronger s-orbital contribution in the bonds, which correlates nicely with its solid-state structure, where the degree of pyramidalization as is diminished by steric constraints. We assume that the cause for the larger ^1^ *J*_29Si‑119Sn_ coupling constants of **16**, **17**, **18**, and **19** is also a diminished degree of pyramidalization. This argumentation is not entirely supported by the crystallographic data but since coupling constants are determined in solution other effects may be in operation.

In our previous study we have shown that the degree of interaction of the [(Me_3_Si)_3_Sn]^−^ unit with the alkali metal counterion has a huge influence on the ^1^ *J*_29Si‑119Sn_ coupling constants with values from ranging from 107 Hz for (Me_3_Si)_3_SnLi·3THF to 349 Hz observed for separated ion pairs where the Cs^+^ counterion is encapsulated in a [2.2.2]­crypt unit.^22^ Even smaller coupling constants as low as 82 Hz were observed by Sarazin and co-workers in their study of the alkaline-earth derivatives of the tris­(trimethylsilyl)­stannide.^23^ We therefore assume that the Sn–K interactions in the current study also have substantial influence on the degree of pyramidalization and thus the magnitude of the ^1^ *J*_29Si‑119Sn_ coupling constants.

It is unfortunate that the ^119^Sn-NMR spectroscopic characterization of related compounds such as [(Me_3_Si)_3_Si]_3_SnLi·THF,^16^ {[(Me_3_Si)_3_Si]_3_SnK}_2_,^18^ [(Me_3_Si)_3_Si]_3_SnK·benzene,^17^ and the variants of (^*t*^Bu_2_MeSi)_3_SnLi^15^ is either nonexistent or not very exhaustive. For this reason, it is difficult to check whether the observed trends with respect to ^1^ *J*_29Si‑119Sn_ coupling constants are rather common.

### X-ray Crystallography

Structural aspects of the neutral compounds **3**, **6**, **7**, **8** and **14** were already discussed in a comparative study of silyl-substituted silanes, germanes and stannanes.^29^ Herein, we report the structures of stannides **1**, **11**, **12**, **15**, **16**, **17**, and **18** (Figures 1–7).

Compound **1** crystallizes in the triclinic space group P-1 with four independent molecules in the asymmetric unit. It likely undergoes a phase transition at a temperature somewhat warmer than 100 K, requiring a higher measurement temperature. Compared to the previously reported 18-crown-6 adducts of potassium tris­(trimethylsilyl)­silanide^50^ and potassium tris­(trimethylsilyl)­germanide,^51^ the degree of pyramidalization increases from Si to Sn with sums of bond angles from 305.93(8) deg for Si, 302.64(3) deg for Ge to 286.9(1) deg for Sn (**1**) (Table 3). This value is even lower than the 296.29(7) deg found for (MeSi_3_)_3_SnLi·(THF)_3_ ^12^ and the 297.2(3) deg for (MeSi_3_)_3_SnNa·15-c-5.^22^ Such behavior points to a high degree of p-character in the Si–Sn bonds. This goes along with a small but noticeable elongation of the Sn–Si bond distances which range from 2.5805(14) to 2.6019(14) Å in comparison to a range of 2.563(2) to 2.581(3) Å for (MeSi_3_)_3_SnLi·(THF)_3_ ^12^ and 2.563(6) to 2.593(5) Å for (MeSi_3_)_3_SnNa·15-c-5.^22^

**Table 3:** Structural Details of Potassium Stannides **1**, **11**, **12**, **15**, **16**, **17**, and **18**, and the Related Compounds (Me_3_Si)_3_EK·18-Crown-6 (E = Si, Ge) and (Me_3_Si)_3_SnNa·15-Crown-5 and (Me_3_Si)_3_SnLi·12-Crown-4

R_*n*_(Me_3_Si)_3–*n*_EK·18-c-6	*d*_E‑SiMe3_ [Å]	*d*_E‑R_ [Å]	*d*_E‑K_ [Å]	Σ Sn-bond angles [°]	ref.
(MeSi_3_)_3_SiK·18-c-6	2.346(1)–2.350(2)	n.a.	3.447(3)	305.93(8)	^50^
(MeSi_3_)_3_GeK·18-c-6	2.3803(9)–2.3876(9)	n.a.	3.3995(7)	302.64(3)	^51^
(MeSi_3_)_3_SnLi·(THF)_3_	2.563(2)–2.581(3)(Li)	n.a.	2.865(5)Li	296.29(7)	^12^
(MeSi_3_)_3_SnNa·15-c-5	2.563(6)–2.593(5) (Na)	n.a.	3.077(2)Na	297.2(3)	^22^
(MeSi_3_)_3_SnK·18-c-6 (**1**)	2.5805(14)–2.6019(14)(K)	n.a.	3.6463(11)	289.27(4)	this work
(MeSi_3_)Ph_2_SnK·18-c-6 (**11**)	2.6286(11)	2.229(4)/2.224(4)	3.7366(9)	289.4(1)	this work
(MeSi_3_)_2_(*^t^* BuMe_2_Si)SnK·18-c-6 (**12**)	2.5993(7)/2.6102(7)	2.6006(7)	3.7083(6)	290.19(2)	this work
(MeSi_3_)(*^i^* Pr_3_Si)_2_SnK·18-c-6 (**15**)	2.6125(19)	2.6335(17)/2.614(2)	3.7866(16)	319.44(7)	this work
(MeSi_3_)_3_Sn(Me_3_Si)_2_SnK·18-c-6 (**16**)	2.5942(19)–2.6057(19)	2.8257(7)–2.8404(7)	3.6443(13)–3.6844(14)	289.62(7)–291.02(7)	this work
(MeSi_3_)_3_Si(Me_3_Si)_2_SnK·18-c-6 (**17**)	2.6184(7)/2.6124(7)	2.6263(6)	6.1783(8)	301.978(3)	this work
[(MeSi_3_)_2_SnSn(SiMe_3_)_2_]K_2_ (18-c-6)_2_ (**18**)	2.6004(5)–2.6112(5)	2.9027(2)/2.9039(2)	3.5887(4)–3.6214(4)	280.65(2)–285.86(2)	this work

A similar sum of bond angles around tin was also observed for stannide **11** (289.4(1) deg) which crystallizes in the monoclinic space group Pn. Somewhat counterintuitively, with 2.6286(11) Å the Sn–Si bond distances of **11** are the longest in the whole series. However, also the Sn–C distances of 2.229(4)/2.224(4) Å to the attached phenyl groups are clearly elongated compared to the 2.14 Å typically found for Sn-Ph bond lengths.

Stannide **12**, where formally one of the SiMe_3_ groups of **1** is replaced by a SiMe_2_^*t*^Bu unit, crystallizes in the monoclinic space group *P*2_1_/*c*. This increased steric bulk does neither affect the sum of bond angles around tin (290.19(2) deg) nor the Sn–Si bonding distances of 2.5993(7) to 2.6102(7) Å.

This behavior changes with stannide **15**, which crystallizes in the orthorhombic space group *P*2_1_2_1_2_1_. In contrast to the increased degree of pyramidalization for **1**, **11**, **12**, **16** and even more so **18** (see Table 3), for **15** the sum of bond angles around tin increases strongly to 319.44(7) deg, indicating a much more flattened geometry. This is most likely caused by the steric interaction of the two triisopropylsilyl groups which widen the angle between them to 110.33(6) deg. Similar flattening effects have been observed for the tris­[tris­(trimethylsilyl)­silyl]­stannides of lithium and potassium^16,18^ and even more pronounced the structures of (^*t*^Bu_2_MeSi)_3_SnLi.^15^ Interestingly, the Si–Sn distances of **15** fit very much into the rest of the samples, indicating that the steric repulsion within the molecule mainly causes geometric distortion before bond elongation.

Stannide **16**, derived from hexakis­(trimethylsilyl)­distannane **6**, was found to crystallize in the monoclinic space group P2_1_/n with 5 independent molecules in the asymmetric unit. The sums of bond angles around the metalated Sn atoms range from 289.62(7) to 291.02(7) deg (Table 3), indicating a similar degree of pyramidalization as was found for **1**, **11**, and **12**. This clearly shows that the steric demand of a single tris­(trimethylsilyl)­stannyl group together with two trimethylsilyl units is not sufficient to cause a noticeable decrease of pyramidalization. The Me_3_Si–Sn bond distances to the metalated tin atom of **16** range from 2.594(2) to 2.606(2) Å which is somewhat elongated compared to the distances to the neutral tin atom which cover a range from 2.567(2) to 2.594(2) Å and to the related bond in **6** of 2.5707(17) Å.^29^ This slight elongation is also visible in the Sn–Sn distances which range from 2.8258(7) to 2.8404(7) Å in comparison to the 2.7930(12) Å in **6**.^29^

Stannide **17**, derived from hexakis­(trimethylsilyl)­silastannane **7**, crystallized in the monoclinic space group *P*2_1_/*c* as a separated ion pair. The potassium atom in the K·18-c-6 unit is coordinated with one disordered THF molecule on one side of the crown ether, while there seems to be a weak interaction to a methyl group of the tris­(trimethylsilyl)­silyl unit on the other side. While all other stannides in this study display Sn–K distances in the range between 3.58 and 3.78 A (Table 3) the respective distance in **17** is close to 6.2 A. The degree of pyramidalization as indicated by a sum of bond angles around the metalated Sn atom of 301.978(3) deg (Table 3), is noticeable smaller than in **16** showing that the steric effect of a tris­(trimethylsilyl)­silyl group is more pronounced than that of the tris­(trimethylsilyl)­stannyl in **16**, as the shorter Sn–Si and Si–Si bonds allow for a more effective steric interaction. Nevertheless, the effect of one tris­(trimethylsilyl)­silyl group together with two trimethylsilyl units in **17** is still not close to the decrease of pyramidalization caused by the interaction of three tris­(trimethylsilyl)­silyl groups such as in [(Me_3_Si)_3_Si]_3_SnLi·THF^16^ or [{[(Me_3_Si)_3_Si]_3_Sn}_2_K]­[K·(THF)_6_],^18^ with sums of bond angles of 334.33(8) and 325.74(1) deg, respectively.

The distannane diide **18**, crystallizing in the triclinic space group P-1 features one and a half molecule in the asymmetric unit with the half molecule sitting on the inversion center. Compared to the 2.7930(12) Å in **6** ^29^ and the 2.8258(7) to 2.8404(7) Å in **16**, the Sn–Sn bond in **18** is further elongated to 2.9027(2)/ 2.9039(2) Å (Table 3). Pyramidalization of tin is further increased as shown by small sums of angles around the tin atoms of 280.65(2) to 285.86(2) deg.

The dianionic compound **19**, derived from **7**, crystallizes in the triclinic space group P-1 with cell constants very close to that of **18**. Its structure could be solved, but the central atoms Sn(1) and Si(1) were found to be disordered over the two central positions in both molecules of **19** (Figure S70 displays a part of the molecular structure of **19**). For this reason, a meaningful discussion of bond lengths and angles cannot be undertaken.

## Conclusions

The chemistry of silylated stannanes is still not a very mature field. In order to develop synthetic strategies to these compounds, which allow for more selectivity than Wurtz type coupling, the reactions of stannides with electrophiles is an excellent strategy.^25^ The number of known silylated stannides is again not very high^52^ and therefore research devoted into this direction seems worthwhile. In the present account we have demonstrated how versatile the method of stannide formation via the trimethylsilyl group abstraction with ^*t*^BuOK is. Starting from (Me_3_Si)_4_Sn facile access to (Me_3_Si)_3_SnK is possible, which can be converted to more diverse tetrasilylated stannanes (Me_3_Si)_3_SnSiR_3_ by reaction with R_3_SiCl. Alternatively, reactions with other electrophiles allow for access to compounds of the general formula (Me_3_Si)_3_SnR. Both types of compounds: (Me_3_Si)_3_SnSiR_3_ and (Me_3_Si)_3_SnR can further be subjected to reactions with ^*t*^BuOK yielding a set of different silylated stannides.

The NMR spectroscopic and crystallographic characterization of these stannides provide valuable insights into their electronic and structural properties. In particular it is possible to link the observed ^1^ *J*_29Si‑119Sn_ coupling constants of the stannanes to the degree of pyramidalization of the anion, which is dependent on the steric interactions of the attached groups (i.e., large substituents prevent strong pyramidalization) and the interaction with the alkali metal counterion.

The range of described stannides in this report should permit synthetic access not only to a wide variety of structurally diverse silylated stannanes but also to a number of low-valent silylated tin compounds, which might be within reach from the dianionic compounds described.

## Experimental Section

All reactions were carried out in a glovebox under an atmosphere of dry nitrogen. Solvents were dried using a column solvent purification system.^53^ Tetrakis­(trimethylsilyl)­stannane,^27^ triisopropylchlorosilane,^54^ tris­(trimethylsilyl)­chlorosilane,^30^ tris­(trimethylsilyl)­chlorogermane^4^ and diphenylbis­(trimethylsilyl)­stannane^32^ were prepared following published procedures. All other chemicals were used as received from different chemical suppliers. Elemental analyses were attempted with a Heraeus VARIO ELEMENTAR EL analyzer. No satisfactory elemental analyses of the alkali metal stannides could be obtained

^1^H (300 or 400 MHz), ^13^C (75.4 or 100.5 MHz), ^29^Si (59.3 or 79.1 MHz), and ^119^Sn (111.8 or 149.1 MHz) NMR spectra were recorded either on a Varian MercuryPlus 300 MHz, a Varian INOVA 300 MHz, a Jeol JNM-ECZL 400 MHz or a Quad 400 MHz either in deuterated solvents or with a D_2_O-cappillary providing an external lock signal. All spectra are referenced to Me_4_Si or Me_4_Sn using the lock frequency signal or solvent signals as reference.^55^ To eliminate the temperature dependence of chemical shifts, spectra were recorded at 25 °C and samples were allowed to equilibrate thermally for 10 min. To compensate for the low isotopic abundance of ^29^Si the INEPT pulse sequence was used for the amplification of the signal.^56,57^ Completeness of conversion was usually controlled by NMR spectroscopy. For X-ray structure analysis crystals were mounted either onto the tips of glass fibers or using loops. Data collection was performed with either a BRUKER-AXS APEX II diffractometer using graphite monochromated MoK_α_ radiation (0.71073 Å) or a Rigaku XtaLAB Synergy Dualflex HyPix-Arc 100 with CuK_α_ radiation (1.54056 Å). Data were reduced to F^2^_o_ and corrected for absorption effects with SAINT^58^ and SADABS,^59,60^ or Crysalis Pro,^61^ respectively. Structures was solved using SHELXT^62^ and refined by full-matrix least-squares methods (SHELXL)^63^ as implemented in OLEX2.^64^ All non-hydrogen atoms were refined with anisotropic displacement parameters. All hydrogen atoms were located in calculated positions to correspond to standard bond lengths and angles.

### Tris­(trimethylsilyl)­stannyl Potassium·18-Crown-6 (Me_3_Si)_3_SnK·18-Crown-6 **1**

To a solution of (Me_3_Si)_4_Sn (100 mg, 0.243 mmol) in benzene (3 mL), 18-crown-6 (67 mg, 0.255 mmol, 1.05 equiv) was added. After addition of ^*t*^BuOK (27 mg, 0.255 mmol, 1.05 equiv) the reaction mixture rapidly turned yellow. Within 30 min complete conversion was detected spectroscopically. NMR data (δ in ppm, rel. to TMS): ^1^H (C_6_D_6_): 3.18 (s, 24H, C*H*_2_O), 0.80 (s, 27H, (C*H*_3_)_3_Si); ^13^C­{H} (C_6_D_6_): 70.1 (*C*H_2_O), 8.8 (*Me*_3_Si); ^29^Si­{H} (C_6_D_6_): −12.5 (*Me*_3_ *Si*, ^1^ *J*_29Si‑117/119Sn_ = 239/250 Hz); ^119^Sn­{H} (C_6_D_6_): −893.2 (Si*Sn*, ^1^_*J*29Si‑119Sn_ = 270 Hz). NMR data in accordance to ref. 22.

### (*tert*-Butyldimethylsilyl)­tris­(trimethylsilyl)­stannane (^t^BuMe_2_Si)­(Me_3_Si)_3_Sn **2**

At room temperature a solution of **1** (2.43 mmol) in benzene (prepared *in situ* from 1.00 g (Me_3_Si)_4_Sn) is added to a solution of *tert*-butyldimethylchlorosilane (385 mg, 0.255 mmol, 1.05 equiv) in benzene at a rate to remain the resulting solution colorless. During the addition a colorless precipitate is formed. After complete addition, the reaction mixture is stirred for 45 min followed by evaporation of all volatiles *in vacuo*. The colorless residue is extracted three times with 10 mL portions of *n*-pentane each. Salts are removed by filtration and the resulting solution is concentrated to incipient crystallization. Storage at −30 °C yields **2** (994 mg, 0.22 mmol, 86% yield) as colorless crystals, mp 265 °C (decomp). E.A.: C: 39.72 H: 9.33 (calcd.) C: 39.56 H: 9.51 (found). NMR data (δ in ppm): ^1^H (C_6_D_6_): 1.00 (s, 9H, (C*H*_3_)_3_C), 0.40 (s, 27H, (C*H*_3_)_3_Si) 0.31 (s, 6H, ^*t*^Bu­(C*H*_3_)_2_Si); ^13^C­{H} (CDCl_3_): 27.8 (*Me*_3_C), 18.4 (Me_3_ *C*), 4.5 (*Me*_3_Si), 0.3 (^*t*^Bu*Me*_2_Si); ^29^Si­{H} (CDCl_3_): 10.3 (^*t*^BuMe_2_ *Si*, ^1^ *J*_29Si‑117/119Sn_ = 327/343 Hz), −9.8 (*Me*_3_ *Si*, ^1^ *J*_29Si‑117/119Sn_ = 323/338 Hz); ^119^Sn­{H} (CDCl_3_): −673.1 ((Me_3_Si)_3_ *Sn* ^1^ *J*_29Si‑119Sn_ = 338 Hz, ^*t*^BuMe_2_Si*Sn* ^1^ *J*_29Si‑119Sn_ = 343 Hz).

### (Triisopropylsilyl)­tris­(trimethylsilyl)­stannane ^i^Pr_3_Si­(Me_3_Si)_3_Sn **3**

To a solution of triisopropylchlorosilane (1000 mg, 5.18 mmol, 1.02 equiv) in benzene, a solution of **1** (5.10 mmol) (prepared *in situ* from 2.10 g (Me_3_Si)_4_Sn) in benzene is added dropwise at ambient temperature to form a pale yellow solution and a colorless precipitate. After complete addition, stirring is continued for an additional 2 h after which all volatiles are removed *in vacuo*. The residue is extracted three times with 10 mL portions of *n*-pentane. Removal of the salts by filtration yields a pale yellow solution which is concentrated to incipient crystallization. Storage at −20 °C yields **3** (1997 mg, 4.03 mmol, 79% yield) as colorless crystals, mp 285 °C (decomp). E.A.: C: 43.62 H: 9.76 (calcd.) C: 43.13 H: 10.01 (found). NMR data (δ in ppm): ^1^H (C_6_D_6_): 1.27 (septet, ^3^ *J*_1H‑1H_ = 5.6 Hz, 3H, (CH_3_)_2_C*H*), 1.15 (d, ^3^ *J*_1H‑1H_ = 5.6 Hz, 18H, (C*H*_3_)_2_CH), 0.42 (s, 27H, (*H*_3_C)_3_Si); ^13^C­{H} (CDCl_3_): 20.7 (*Me*_2_CH), 14.6 (Me_2_ *C*H), 4.9 (*Me*_3_Si); ^29^Si­{H} (C_6_D_6_): 29.3 (iPr_3_ *Si*, ^1^ *J*_29Si‑117/119Sn_ = 322/337 Hz), −11.1 (Me_3_ *Si*, ^1^ *J*_29Si‑117/119Sn_ = 308/323 Hz); ^119^Sn­{H} (C_6_D_6_): −685.0 (iPr_3_Si*Sn*, ^1^ *J*_29Si‑119Sn_ = 337 Hz, Me_3_Si*Sn*, ^1^ *J*_29Si‑119Sn_ = 323 Hz).

### (Triisopropylstannyl)­tris­(trimethylsilyl)­stannane ^i^Pr_3_Sn­(Me_3_Si)_3_Sn **4**

At room temperature a solution of **1** (2.43 mmol) in benzene (10 mL) (prepared *in situ* from 1.00g (Me_3_Si)_4_Sn) is added to a solution of triisopropylchlorostannane (725 mg, 2.45 mmol, 1.01 equiv) in benzene (15 mL) at a rate to remain the reaction mixture colorless. After complete addition stirring is continued for an additional 5 min, followed by removal of all solids formed by filtration. The solvent is removed *in vacuo* and the residue is extracted three times with 10 mL portions of *n*-pentane. Insoluble material is removed by filtration and the thus obtained solution of **4** in *n*-pentane is concentrated to incipient crystallization. Storage at −20 °C yields **4** (1076 mg, 0.184 mmol, 72% yield) as colorless crystals, mp 243 °C (decomp). E.A.: C: 36.88 H: 8.25 (calcd.) C: 36.34 H: 8.57 (found). NMR data (δ in ppm): ^1^H (C_6_D_6_): 1.57 (septet, ^3^ *J*1_H_-1_H_ = 6.8 Hz, 3H, (CH_3_)_2_C*H*), 1.38 (d, ^3^ *J*_1H‑1H_ = 6.8 Hz, 18H, (C*H*_3_)_2_CH), 0.42 (s, 27H, (*H*_3_C)_3_Si); ^13^C­{H} (pentane/D_2_O-cap): 23.3 (*Me*_2_CH), 16.0 (Me_2_ *C*H), 4.0 (*Me*_3_Si); ^29^Si­{H} (CDCl_3_): −8.7 (Me_3_ *Si*, ^1^ *J*_29Si‑117/119Sn_ = 309/325 Hz); ^119^Sn­{H} (pentane/D_2_O-cap): −10.7 (iPr_3_ *Sn*, ^1^ *J*_117/119Sn‑119Sn_ = 510/536 Hz), −701.7 (Si*Sn*, ^1^ *J*_29Si‑119Sn_ = 327 Hz, Si*Sn*, ^1^ *J*_117Sn‑119Sn_ = 510/535 Hz).

### Methyltris­(trimethylsilyl)­stannane Me­(Me_3_Si)_3_Sn **5**

At room temperature, a solution of **1** (6.08 mmol) in benzene (prepared *in situ* from 2.50g (Me_3_Si)_4_Sn) is added dropwise to a solution of dimethyl sulfate (865 mg, 0.65 mL, 6.85 mmol, 1.13 equiv) in benzene at a rate to remain the resulting reaction mixture colorless. In the course of addition, a colorless precipitate forms. After complete addition the reaction mixture is stirred for 20 min after which all volatiles are removed *in vacuo*. The colorless residue is extracted 3 times with 10 mL portions of *n*-pentane. After filtration all volatiles are removed *in vacuo* to leave **5** (178 mg, 5.04 mmol, 83 yield) as a colorless oil. E.A.: C: 33.99 H: 8.53 (calcd.) C: 34.26 H: 8.77 (found). NMR data (δ in ppm, rel. to TMS): ^1^H (C_6_H_6_): 0.36 (s, 3H, C*H*_3_Sn), 0.31 (s, 27H, (C*H*_3_)_3_Si); ^13^C­{H} (C_6_D_6_): 4.3 (*Me*Sn), 2.6 (*Me*_3_Si); ^29^Si­{H} (C_6_D_6_): −10.1 (*Me*_3_ *Si*, ^1^ *J*_29Si‑117/119Sn_ = 404/423 Hz); ^119^Sn­{H} (C_6_D_6_): – 457.4 (Si*Sn*, ^1^ *J*_29Si‑119Sn_ = 476 Hz).

### Hexakis­(trimethylsilyl)­distannane (Me_3_Si)_3_SnSn­(SiMe_3_)_3_ **6**

At –40 °C a solution of **1** (2.43 mmol) (prepared *in situ* from 1.00 g (Me_3_Si)_4_Sn) in toluene (15 mL) is added dropwise to a solution of 1,2-dibromoethane (0.23 mL, 500 mg, 2.66 mmol, 1.10 equiv) in diethyl ether (10 mL). After complete addition the reaction mixture is warmed up to room temperature and immediately after all volatiles are removed *in vacuo* to avoid decomposition of the product. The residue is extracted three times with 10 mL portions of *n*-pentane. All solids are thoroughly removed by filtration. The *n*-pentane solution is concentrated to incipient crystallization. Storage at –30 °C yields **6** (610 mg, 0.90 mmol, 74% yield) as colorless crystals, mp 293 °C (decomp) Lit.:^28^ 290 °C (decomp). E.A.: C: 31.95 H: 8.02 (calcd.) C: 31.67 H: 8.21 (found). NMR data (δ in ppm): ^1^H (C_6_D_6_): 0.44 (s, 54H, (H_3_C)_3_Si); ^13^C­{H} (C_6_D_6_): 5.0 (Me_3_Si); ^29^Si­{H} (C_6_D_6_): −10.0 (Me_3_Si, ^1^ *J*_29Si‑117/119Sn_ = 306/320 Hz); ^119^Sn­{H} (C_6_D_6_): −677.8 (SiSn, ^1^ *J*_29Si‑119Sn_ = 320 Hz, ^1^ *J*_117Sn‑119Sn_ = 87 Hz).

### Hexakis­(trimethylsilyl)-2-siladistannane (Me_3_Si)_3_SiSn­(SiMe_3_)_3_ **7**

At ambient temperature a solution of **1** (2.43 mmol) (prepared *in situ* from 1.00 g (Me_3_Si)_4_Sn) in toluene (15 mL) is added to a solution of tris­(trimethylsilyl)­chlorosilane (690 mg, 2.43 mmol) in toluene (10 mL). After complete addition, stirring is continued for 15 min after which all volatiles are removed *in vacuo*. The colorless residue is extracted three times with 15 mL portions of *n*-pentane. Salts are removed thoroughly by filtration. The pale brown solution is concentrated *in vacuo* to incipient crystallization. Storage at –30 °C for 12 h affords **7** (1150 mg, 1.96 mmol, 81% yield) as colorless crystals, mp 278 °C (decomp). E.A.: C: 36.90 H: 9.29 (calcd.) C: 36.58 H: 9.19 (found). NMR data (δ in ppm): ^1^H (C_6_D_6_): 0.44 (s, 27H, ((*H*_3_C)_3_Si)_3_Si), 0.34 (s, 27H, ((*H*_3_C)_3_Si)_3_Sn); ^13^C­{H} (C_6_D_6_): 5.3 (Me_3_Si)_3_Sn, 3.8 (Me_3_Si)_3_Si; ^29^Si­{H} (C_6_D_6_): – 8.9 ((Me_3_ *Si*)_3_Si, ^2^ *J*_29Si‑117/119Sn_ = 43/45 Hz), −10.3 ((Me_3_ *Si*)_3_Sn, ^1^ *J*_29Si‑117/119Sn_ = 309/324 Hz), −135.4 ((Me_3_Si)_3_ *Si*, ^1^ *J*_29Si‑117/119Sn_ = 300/314 Hz); ^119^Sn­{H} (C_6_D_6_): −627.7 ((Me_3_Si)_3_ *Sn*, ^1^ *J*_29Si‑119Sn_ = 333 Hz, ^1^ *J*_29Si‑119Sn_ = 319 Hz, ^2^ *J*_29Si‑119Sn_ = 45 Hz).

### Hexakis­(trimethylsilyl)-2-germadistannane (Me_3_Si)_3_GeSn­(SiMe_3_)_3_ **8**

To a solution of (Me_3_Si)_4_Sn (120 mg, 0.29 mmol) in benzene (2 mL) potassium *tert*-butoxide (33 mg, 0.29 mmol) and 18-crown-6 (77 mg, 0.29 mmol) are added and the solution was stirred for 30 min. The reaction mixture is then dropwise added to a solution of chloro-tris­(trimethylsilyl)­germane (96 mg, 0.29 mmol) in benzene and stirred for another 30 min. Afterward solvent is removed *in vacuo*, followed by extraction of the residue with *n*-pentane. After centrifugation to remove salts and removal of *n*-pentane, **8** (156 mg, 0.24 mmol, 85%) is obtained as a pale yellow solid. Analytically pure **8** is obtained upon recrystallization from *n*-pentane at −30 °C, mp 265 °C (decomp). E.A.: C: 34.29 H: 8.36 (calcd.) C: 34.51 H: 8.67 (found). NMR data (δ in ppm): ^1^H (C_6_D_6_): 0.45 (s, 27H, ((C*H*_3_)_3_Si)_3_Ge), 0.38 (s, 27H, ((C*H*_3_)_3_Si)_3_Sn); ^13^C­{H} (C_6_D_6_): 5.3 (*Me*_3_Si)_3_Sn, 4.3 (*Me*_3_Si)_3_Ge; ^29^Si (C_6_D_6_): – 4.2 ((Me_3_ *Si*)_3_Ge), −10.4 ((Me_3_ *Si*)_3_Sn, ^1^ *J*_29Si‑117/119Sn_ = 307/321 Hz); ^119^Sn­{H} (C_6_D_6_): −609.0 ((Me_3_Si)_3_ *Sn*, ^1^ *J*_29Si‑119Sn_ = 321 Hz). MS (70 eV) *m*/*z* (%): 339 (49) [SnSi_3_Me_9_H^+^]; 249 (4) [SnSi_2_Me_5_H^+^]; 219 (5) [SnSi_2_Me_3_^+^]; 207 (11) [SnSiMe_4_^+^]; 131 (36) [Si_2_Me_5_^+^]; 73 (100) [SiMe_3_^+^].

### Methylbis­(trimethylsilyl)­stannyl Potassium·18-Crown-6 Me­(Me_3_Si)_2_SnK·18-Crown-6 **9**

To a solution of potassium *tert*-butoxide (85 mg, 0.76 mmol) and 18-crown-6 (200 mg, 0.76 mmol) in benzene (5 mL), **5** (250 mg, 0.71 mmol) is added to produce an intensely yellow solution of **9**. Addition of *n*-pentane results in precipitation of **9** as a microcrystalline solid. NMR data (δ in ppm): ^1^H (C_6_D_6_): 3.49 (s, 24H), 0.28 (s, 18H), 0.14 (s, 3H); ^13^C­{H} (C_6_D_6_): 71.0 (*C*H_2_O), 4.0 (*Me*_3_Si, ^2^ *J*_13Si‑117/119Sn_ = 32 Hz), 2.7 (*Me*Sn); ^29^Si­{H} (C_6_D_6_): −5.6 ((Me_3_ *Si*)_3_Sn, ^1^ *J*_29Si‑117/119Sn_ = 330/346 Hz); ^119^Sn­{H} (C_6_D_6_): −439.1 (^1^ *J*_29Si‑119Sn_ = 347 Hz).

### Dimethylbis­(trimethylsilyl)­stannane Me_2_(Me_3_Si)_2_Sn **10**

A solution of **9** (200 mg, 0.34 mmol) in benzene (5 mL) is added to a solution of dimethyl sulfate (45 mg, 35 μL, 0.35 mmol) in diethyl ether. The reaction mixture is stirred for 30 min after which all volatiles are removed *in vacuo*. The residue is extracted three times with 5 mL portions of *n*-pentane. After removal of the insoluble residue by filtration the solvent is removed *in vacuo*. NMR spectroscopic properties are in full agreement with literature data.^31,32^

### Diphenyl­(trimethylsilyl)­stannyl Potassium 18-Crown-6 Ph_2_(Me_3_Si)_2_Sn K·18-Crown-6 **11**

To a solution of (Me_3_Si)_2_SnPh_2_ (250 mg, 0.60 mmol) in diethyl ether (5 mL), 18-crown-6 (165 mg, 0.63 mmol, 1.05 equiv) and potassium *tert*-butoxide (70 mg, 0.63 mmol, 1.05 equiv) are added with mixing to yield a pale yellow solution. After keeping the reaction mixture at room temperature for 30 min, diethyl ether is evaporated to incipient crystallization. Storage at –30 °C yields **11** (356 mg, 0.55 mmol, 92%) as pale yellow, almost colorless crystals, mp 103 °C (intense red melt). An NMR sample of **11** was prepared from the reaction of (Me_3_Si)_2_SnPh_2_ (100 mg), potassium *tert*-butoxide (28 mg) and of 18-crown-6 (66 mg) in C_6_D_6_ (0.6 mL). NMR data (δ in ppm): ^1^H (C_6_H_6_/D_2_O-cap): 8.15–8.12 (m, 4H), 7.16 (m, 6H), 3.11 (s, 24H, CH_2_O), 0.49 (s, 9H, (C*H*_3_)_3_Si)); ^13^C­{H} (C_6_D_6_/DME): 163.1, 139.9, 126.0, 122.8, 70.2 (*C*H_2_O), 3.5 (*Me*_3_Si); ^29^Si­{H} (C_6_D_6_): – 9.1 ((Me_3_ *Si*), ^1^ *J*_29Si‑117/119Sn_ = 347/364 Hz); ^119^Sn­{H} (C_6_D_6_): −228.4 ((Me_3_Si)_2_ *Sn*, ^1^ *J*_29Si‑119Sn_ = 364 Hz), Ph_2_ *Sn*, ^1^ *J*_13C‑119Sn_ = 283 Hz).

### (*tert*-Butyldimethylsilyl)­bis­(trimethylsilyl)­stannyl Potassium 18-Crown-6 (^t^BuMe_2_Si)­(Me_3_Si)_2_Sn K·18-Crown-6 **12**

To a solution of potassium *tert*-butoxide (86 mg, 0.77 mmol) and 18-crown-6 (204 mg, 0.77 mmol) in benzene, **2** (350 mg, 0.77 mmol) is added to immediately form a bright yellow solution. Conversion to **12** is complete within 5 min. Fine yellow crystals of **12** (420 mg, 62 mmol, 80% yield) precipitate from the benzene solution upon addition of *n*-pentane, mp 138 °C (decomp). NMR data (δ in ppm): ^1^H (C_6_D_6_): 3.22 (s, 24H, CH_2_O), 1.38 (s, 9H, (C*H*_3_)_3_C), 0.81 (s, 18H, (C*H*_3_)_3_Si), 0.78 (s, 6H, ^*t*^Bu­(C*H*_3_)_2_Si); ^13^C­{H} (C_6_D_6_): 70.3 (*C*H_2_O), 29.3 (*Me*_3_C), 18.8 (Me_3_ *C*), 8.9 (*Me*_3_Si), 3.7 (^*t*^Bu*Me*_2_Si); ^29^Si­{H} (C_6_D_6_): 5.6 (^*t*^BuMe_2_ *Si*, ^1^ *J*_29Si‑117/119Sn_ = 237/252 Hz), −13.4 (*Me*_3_ *Si*, ^1^ *J*_29Si‑117/119Sn_ = 245/257 Hz); ^119^Sn­{H} (C_6_D_6_): −908.4 ((Me_3_Si)_2_ *Sn* ^1^ *J*_29Si‑119Sn_ = 281 Hz, ^*t*^BuMe_2_Si*Sn* ^1^ *J*_29Si‑119Sn_ = 252 Hz).

### (Triisopropylsilyl)­bis­(trimethylsilyl)­stannyl Potassium·18-Crown-6 (iPr_3_Si)­(Me_3_Si)_2_SnK·18-Crown-6 **13**

To a solution of potassium *tert*-butoxide (56 mg, 0.504 mmol) and 18-crown-6 (133 mg, 0.504 mmol) in benzene (10 mL) **3** (250 mg, 0.505 mmol) is added. Upon addition a bright orange solution is formed. Formation of **13** completes within 15 min as determined by NMR spectroscopy. **13** (282 mg, 0.39 mmol, 77% yield) can be isolated as the crown ether adduct by removal of solvent to incipient crystallization and cooling to −30 °C, mp 157 °C (decomp). NMR data (δ in ppm): ^1^H (C_6_D_6_): 3.53 (s, 24H, C*H*_2_O), 1.74 (d, ^3^ *J*_1H‑1H_ = 5.2 Hz, 18H, (C*H*_3_)_2_CH), 1.19 (septet, ^3^ *J*_1H‑1H_ = 5.2 Hz, 3H, (CH_3_)_2_C*H*), 0.71 (s, 18H, (*H*_3_C)_3_Si); ^13^C­{H} (C_6_D_6_): 70.4 (*C*H_2_O), 22.3 (*Me*_2_CH), 16.4 (Me_2_ *C*H), 9.4 (*Me*_3_Si); ^29^Si­{H} (C_6_D_6_): 26.2 (iPr_3_ *Si*, ^1^ *J*_29Si‑117/119Sn_ = 283/296 Hz), −16.1 (Me_3_ *Si*, ^1^ *J*_29Si‑117/119Sn_ = 268/281 Hz); ^119^Sn­{H} (C_6_D_6_): −908.6 (iPr_3_Si*Sn*, ^1^ *J*_29Si‑119Sn_ = 296 Hz, Me_3_Si*Sn*, ^1^ *J*_29Si‑119Sn_ = 281 Hz).

### Bis­(triisopropylsilyl)­bis­(trimethylsilyl)­stannane (iPr_3_Si)_2_(Me_3_Si)_2_Sn **14**

A solution of **13** (200 mg, 0.275 mmol) in toluene (10 mL) is added to a solution of triisopropylchlorosilane (58 mg, 0.3 mmol) in toluene (15 mL). The reaction mixture is left stirring at room temperature for 36 h after which all volatiles are removed *in vacuo*. The residue is extracted with *n*-pentane. Concentration and cooling to −80 °C yields **14** (91 mg, 0.157 mmol, 57% yield) as colorless crystals, mp 283 °C (decomp). E.A.: C: 49.72 H: 10.43 (calcd.) C: 49.28 H: 10.71 (found). NMR data (δ in ppm): ^1^H (C_6_D_6_): 1.33 (septet, ^3^ *J*1_H_-1_H_ = 5.6 Hz, 6H, (CH_3_)_2_C*H*), 1.18 (d, ^3^ *J*_1H‑1H_ = 5.6 Hz, 36H, (C*H*_3_)_2_CH), 0.47 (s, 18H, (*H*_3_C)_3_Si); ^13^C­{H} (C_6_D_6_): 20.9 (*Me*_2_CH), 16.0 (Me_2_ *C*H), 5.7 (*Me*_3_Si); ^29^Si­{H} (C_6_D_6_): 25.1 (iPr_3_ *Si*, ^1^ *J*_29Si‑117/119Sn_ = 270/295 Hz), −11.6 (Me_3_ *Si*, ^1^ *J*_29Si‑117/119Sn_ = 274/286 Hz); ^119^Sn­{H} (C_6_D_6_): – 673.8 (iPr_3_Si*Sn*, ^1^ *J*_29Si‑119Sn_ = 301 Hz, Me_3_Si*Sn*, ^1^ *J*_29Si‑119Sn_ = 285 Hz).

### Bis­(triisopropylsilyl)­trimethylsilylstannyl Potassium·18-Crown-6 (iPr_3_Si)_2_(Me_3_Si)­SnK·18-Crown-6 **15**

**14** (50 mg, 0.086 mmol) is added to a solution of potassium *tert*-butoxide (10 mg, 0.087 mmol) and 18-crown-6 (23 mg, 0.087 mmol) in toluene (3 mL) to yield a dark orange solution. Orange-yellow crystals of **15** (55 mg, 0.068 mmol, 79% yield) are obtained upon concentration, addition of *n*-pentane and cooling to −30 °C, mp 142 °C (decomp). NMR data (δ in ppm): ^1^H (C_6_D_6_): 3.15 (s, 24H, C*H*_2_O), 1.44 (d, ^3^ *J*_1H‑1H_ = 5.1 Hz, 36H, (C*H*_3_)_2_CH), 1.40 (septet, ^3^ *J*_1H‑1H_ = 5.1 Hz, 6H, (CH_3_)_2_C*H*), 0.65 (s, 9H, (*H*_3_C)_3_Si); ^13^C­{H} (C_6_D_6_): 69.4 (*C*H_2_O), 22.0 (*Me*_2_CH), 16.6 (Me_2_ *C*H), 9.7 (*Me*_3_Si); ^29^Si­{H} (C_6_D_6_): 23.8 (iPr_3_ *Si*, ^1^ *J*_29Si‑117/119Sn_ = 347/363 Hz), – 19.4 (Me_3_ *Si*, ^1^ *J*_29Si‑117/119Sn_ = 328/344 Hz); ^119^Sn­{H} (C_6_D_6_): – 929.8 (iPr_3_Si*Sn*, ^1^ *J*_29Si‑119Sn_ = 363 Hz, Me_3_Si*Sn*, ^1^ *J*_29Si‑119Sn_ = 344 Hz).

### 1,1,2,2,2-Pentakis­(trimethylsilyl)­distannyl Potassium·18-Crown-6 (Me_3_Si)_3_SnSn­(SiMe_3_)_2_K·18-Crown-6 **16**

To a solution of potassium *tert*-butoxide (42 mg, 0.38 mmol) and 18-crown-6 (98 mg, 0.38 mmol) in toluene (10 mL) **6** (250 mg, 0.37 mmol) is added to immediately form a light red solution. Crystalline **16** (278 mg, 0.31 mmol, 83% yield) is obtained upon concentration *in vacuo*, addition of *n*-pentane and cooling to −30 °C, mp 182 °C (decomp). NMR data (δ in ppm): ^1^H (C_6_D_6_): 3.19 (s, 24H, CH_2_O), 0.79 (s, 18H, (*H*_3_C)_3_Si), 0.70 (s, 27H, (*H*_3_C)_3_Si); ^13^C­{H} (C_6_D_6_): 70.2 (*C*H_2_O), 9.6 ((Me_3_Si)_2_Sn), 5.7 ((Me_3_Si)_3_Sn); ^29^Si­{H} (C_6_D_6_): −12.0 ((Me_3_ *Si*)_2_Sn, ^1^ *J*_29Si‑117/119Sn_ = 330/346 Hz), −14.4 ((Me_3_ *Si*)_2_Sn, ^1^ *J*_29Si‑117/119Sn_ = 193/202 Hz); ^119^Sn­{H} (C_6_D_6_): −725.4 ((Me_3_Si)_3_Si*Sn*, ^1^ *J*_29Si‑119Sn_ = 202 Hz, ^1^ *J*_117/119Sn‑119Sn_ = 4594/4807 Hz), −836.2 ((Me_3_Si)_2_Si*Sn*, ^1^ *J*_29Si‑119Sn_ = 346 Hz, ^1^ *J*_117/119Sn‑119Sn_ = 4604/4803 Hz).

### 1,1,2,2,2-Pentakis­(trimethylsilyl)-2-siladistannyl Potassium·18-Crown-6 (Me_3_Si)_3_SiSn­(SiMe_3_)_2_K·18-Crown-6 **17**

To a solution of potassium *tert*-butoxide (48 mg, 0.43 mmol) and 18-crown-6 (115 mg, 0.44 mmol) in toluene (10 mL) **7** (250 mg, 0.43 mmol) is added to immediately form an orange solution. Orange yellow crystals of **17** (242 mg, 0.30 mmol, 69% yield) are obtained upon concentration *in vacuo*, addition of *n*-pentane and cooling to −30 °C, mp 197 °C (decomp). NMR data (δ in ppm): ^1^H (C_6_D_6_): 3.43 (s, 24H, C*H*_2_O), 0.73 (s, 18H, ((*H*_3_C)_3_Si)_3_Sn), 0.58 (s, 27H, ((*H*_3_C)_3_Si)_3_Si); ^13^C­{H} (C_6_D_6_): 70.7 (*C*H_2_O), 9.5 (*Me*_3_Si)_2_Sn, 4.6 (*Me*_3_Si)_3_Si; ^29^Si­{H} (C_6_D_6_): −9.1 ((Me_3_ *Si*)_3_Si, ^2^ *J*_29Si‑117/119Sn_ = 21/22 Hz), −15.1 ((Me_3_ *Si*)_3_Sn, ^1^ *J*_29Si‑117/119Sn_ = 332/348 Hz), – 152.6 ((Me_3_Si)_3_ *Si*, ^1^ *J*_29Si‑117/119Sn_ = 330/345 Hz); ^119^Sn­{H} (C_6_D_6_): −812.9 ((Me_3_Si)_2_ *Sn*, ^1^ *J*_29Si‑119Sn_ = 348 Hz, ^1^ *J*_29Si‑119Sn_ = 345 Hz, ^2^ *J*_29Si‑119Sn_ = 22 Hz).

### 1,1,2,2-Tetrakis­(trimethylsilyl)­distanna-1,2-diyl Dipotassium·2 × 18-Crown-6 K­(Me_3_Si)_2_SnSn­(SiMe_3_)_2_K·2 × 18-Crown-6 **18**

To a solution of potassium *tert*-butoxide (85 mg, 0.76 mmol, 2.05 equiv) and 18-crown-6 (200 mg, 0.76 mmol, 2.05 equiv) in toluene (15 mL) **6** (250 mg, 0.37 mmol) is added to immediately form a light red solution. Upon standing at room temperature the reaction to form **18** completes within 6 h. Upon standing for 24 h at room temperature, crystals of **18** precipitate from solution. A second crop of product is obtained upon concentration and layering the toluene solution with *n*-pentane. Overall yield: 375 mg, 0.33 mmol, 89% yield, mp 164 °C (decomp). NMR data (δ in ppm): ^1^H (C_6_D_6_): 3.36 (s, 48H, CH_2_O), 0.95 (s, 36H, (*H*_3_C)_3_Si); ^13^C­{H} (C_6_D_6_): 70.0 (*C*H_2_O), 9.9 (Me_3_Si)_2_Sn; ^29^Si­{H} (C_6_D_6_): −17.6 ((Me_3_ *Si*)_2_Sn, ^1^ *J*_29Si‑117/119Sn_ = 400/419 Hz); ^119^Sn­{H} (C_6_D_6_): −899.6 ((Me_3_Si)_2_Si*Sn*, ^1^ *J*_29Si‑119Sn_ = 419 Hz, ^1^ *J*_117Sn‑119Sn_ = 986 Hz).

### 1,1,2,2-Tetrakis­(trimethylsilyl)-2-siladistanna-1,2-diyl Dipotassium·2 × 18-Crown-6 K­(Me_3_Si)_2_SiSn­(SiMe_3_)_2_K·2 × 18-Crown-6 **19**

To a solution of potassium *tert*-butoxide (98 mg, 0.87 mmol) and 18-crown-6 (230 mg, 0.87 mmol) in toluene (15 mL) **7** (250 mg, 0.43 mmol) is added to immediately form a light red solution. Upon standing at room temperature, the reaction to form **19** completes within 18 h. After 24 h at room temperature, crystals of **19** precipitate from solution. A second crop of product is obtained upon concentration and cooling to −30 °C. Overall yield: 378 mg, 0.36 mmol, 84% yield, mp 173 °C (decomp). NMR data (δ in ppm): ^1^H (C_6_D_6_): 3.37 (s, 48H, C*H*_2_O), 0.92 (s, 18H, ((*H*_3_C)_3_Si)_2_Si), 0.83 (s, 18H, ((*H*_3_C)_3_Si)_3_Sn); ^13^C­{H} (C_6_D_6_): 70.0 (*C*H_2_O), 9.5 (*Me*_3_Si)_2_Si, 9.0 (*Me*_3_Si)_2_Sn; ^29^Si­{H} (C_6_D_6_): −2.5 ((Me_3_ *Si*)_2_Si, ^2^ *J*_29Si‑117/119Sn_ = 36/38 Hz), −18.4 ((Me_3_ *Si*)_3_Sn, ^1^ *J*_29Si‑117/119Sn_ = 398/416 Hz), −205.5 ((Me_3_Si)_3_ *Si*, ^1^ *J*_29Si‑117/119Sn_ = 117/122 Hz); ^119^Sn­{H} (C_6_D_6_): −775.1 ((Me_3_Si)_2_ *Sn*, ^1^ *J*_29Si‑119Sn_ = 348 Hz, ^1^ *J*_29Si‑119Sn_ = 122 Hz, ^2^ *J*_29Si‑119Sn_ = 38 Hz).

## Supplementary Material



## Notes

Crystallographic tables for compounds **1**, **11**, **12**, **15**, **16**, **17**, **18**, and **19** and ^1^H, ^13^C, ^29^Si-INEPT, and ^119^Sn NMR spectra for all characterized compounds (PDF)
