# Supplementary material for: Silylated Stannanes and Stannides
Source: Inorg Chem. 2026 Apr 22;65(17):9482–93. doi: 10.1021/acs.inorgchem.6c00724 (PMC13147333; doi:10.1021/acs.inorgchem.6c00724)
Supplement: Supplementary file 1 [file ic6c00724_si_001.pdf]

Supporting Information to

Silylated Stannanes and Stannides

Roland C. Fischer* and Christoph Marschner*

Institut für Anorganische Chemie, Technische Universität Graz, Stremayrgasse 9,
8010 Graz, Austria

e-mail: roland.fischer@tugraz.at, christoph.marschner@tugraz.at

Contents

1. Crystallographic tables of compounds **1**, **11**, **12, 15, 16, 17, 18** and **19** **S2,3**
2. NMR spectra **S4**
   - ^{1}H, ^{13}C, ^{29}Si, and ^{119}Sn spectra **1** **S4**
   - ^{1}H, ^{13}C, ^{29}Si, and ^{119}Sn spectra **2** **S6**
   - ^{1}H, ^{13}C, ^{29}Si, and ^{119}Sn spectra **3** **S8**
   - ^{1}H, ^{13}C, ^{29}Si, and ^{119}Sn spectra **4** **S10**
   - ^{1}H, ^{13}C, ^{29}Si, and ^{119}Sn spectra **5** **S12**
   - ^{1}H, ^{13}C, ^{29}Si, and ^{119}Sn spectra **6** **S14**
   - ^{1}H, ^{13}C, ^{29}Si, and ^{119}Sn spectra **7** **S16**
   - ^{1}H, ^{13}C, ^{29}Si, and ^{119}Sn spectra **8** **S18**
   - ^{1}H, ^{13}C, ^{29}Si, and ^{119}Sn spectra **11** **S20**
   - ^{1}H, ^{13}C, ^{29}Si, and ^{119}Sn spectra **12** **S22**
   - ^{1}H, ^{13}C, ^{29}Si, and ^{119}Sn spectra **13** **S24**
   - ^{1}H, ^{13}C, ^{29}Si, and ^{119}Sn spectra **14** **S26**
   - ^{1}H, ^{13}C, ^{29}Si, and ^{119}Sn spectra **15** **S28**
   - ^{1}H, ^{13}C, ^{29}Si, and ^{119}Sn spectra **16** **S30**
   - ^{1}H, ^{13}C, ^{29}Si, and ^{119}Sn spectra **17** **S32**
   - ^{1}H, ^{13}C, ^{29}Si, and ^{119}Sn spectra **18** **S34**
   - ^{1}H, ^{13}C, ^{29}Si, ^{1}H-^{29}Si HMBC and ^{119}Sn spectra **19** **S36**
3. Molecular structure of **19** in the solid state **S39**

1. Crystallographic Tables

Table S1. Crystallographic data for compounds **1**, **11**, **12,** and **15**.

Compound	**1**	**11**	**12**	**15**
Empirical formula	$C_{21}H_{51}O_6Si_3KSn$	$C_{27}H_{43}O_6SiKSn$	$C_{24}H_{57}KO_6Si_3Sn$	$C_{33}H_{75}KO_6Si_3Sn$
Formula weight	641.67	649.49	683.75	809.99
Temperature/K	140	100.01	99.55	99.98
Crystal system	Triclinic	monoclinic	monoclinic	orthorhombic
Space group	P-1	Pn	$P2_1/c$	$P2_12_12_1$
a/Å	19.93000(10)	10.9199(10)	15.8383(13)	14.2117(12)
b/Å	20.0755(2)	10.3573(9)	14.0976(12)	17.0677(15)
c/Å	21.28460(10)	14.0071(13)	16.2047(16)	17.9078(15)
α/°	102.1580(10)	90	90	90
β/°	103.8580(10)	92.131(6)	97.190(3)	90
γ/°	117.2630(10)	90	90	90
Volume/Å^3	6824.70(11)	1583.1(2)	3589.8(6)	4343.7(6)
Z	8	2	4	4
ρ_{calc}g/cm^3	1.249	1.363	1.265	1.239
μ/mm^{-1}	8.282	1.011	0.958	0.802
F(000)	2688	672	1440	1728
Crystal size/mm^3	0.23 × 0.19 × 0.16	0.42 × 0.33 × 0.12	0.22 × 0.18 × 0.13	0.19 × 0.17 × 0.04
Radiation	CuKα (λ = 1.54184)	MoKα (λ = 0.71073)	MoKα (λ = 0.71073)	MoKα (λ = 0.71073)
2Θ range for data collection/°	5.606 to 139.996	4.892 to 59.678	3.842 to 60.112	3.296 to 55.21
Index ranges	$-24 \leq h \leq 24$, $-24 \leq k \leq 24$, $-25 \leq l \leq 25$	$-15 \leq h \leq 15$, $-14 \leq k \leq 14$, $-19 \leq l \leq 19$	$-22 \leq h \leq 20$, $-19 \leq k \leq 19$, $-22 \leq l \leq 22$	$-18 \leq h \leq 18$, $-21 \leq k \leq 22$, $-23 \leq l \leq 23$
Reflections collected	333460	24460	222931	181590
Independent reflections	25861 [R_{int} = 0.1319, R_{sigma} = 0.0435]	8020 [R_{int} = 0.0330, R_{sigma} = 0.0417]	10490 [R_{int} = 0.0828, R_{sigma} = 0.0358]	9955 [R_{int} = 0.1171, R_{sigma} = 0.0645]
Data/restraints/parameters	25861/0/1189	8020/2/328	10490/108/490	9955/0/412
Goodness-of-fit on F^2	1.055	1.039	1.162	1.083
Final R indexes [I>=2σ (I)]	R_1 = 0.0644, wR_2 = 0.1749	R_1 = 0.0302, wR_2 = 0.0665	R_1 = 0.0427, wR_2 = 0.0635	R_1 = 0.0597, wR_2 = 0.1113
Final R indexes [all data]	R_1 = 0.0703, wR_2 = 0.1802	R_1 = 0.0388, wR_2 = 0.0700	R_1 = 0.0602, wR_2 = 0.0692	R_1 = 0.0808, wR_2 = 0.1185
Largest diff. peak/hole / e Å^{-3}	2.85/-1.50	1.11/-0.63	1.14/-0.84	1.32/-0.97

Table S2. Crystallographic data for compounds **16**, **17**, **18**, and **19**.

Compound	**16**	**17**	**18**	**19**
Empirical formula	$C_{27}H_{69}KO_6Si_5Sn_2$	$C_{31}H_{77}KO_7Si_6Sn$	$C_{60}H_{132}K_3O_{18}Si_6Sn_3$	$C_{60}H_{132}K_3O_{18}Si_{7.5}Sn_{1.5}$
Formula weight	906.75	888.25	1783.56	1647.66
Temperature/K	99.68	100.0(4)	100(2)	100.0(3)
Crystal system	monoclinic	monoclinic	triclinic	triclinic
Space group	$P2_1/n$	$P2_1/c$	P-1	P-1
a/Å	21.3121(14)	10.07134(7)	12.0626(2)	11.99440(10)
b/Å	21.5359(14)	17.13062(12)	20.0301(4)	19.9376(2)
c/Å	50.909(3)	28.41826(19)	20.5286(4)	20.4343(2)
α/°	90	90	114.9330(10)	114.5570(10)
β/°	97.504(3)	90.4883(6)	92.3570(10)	92.1870(10)
γ/°	90	90	101.1640(10)	101.4680(10)
Volume/$Å^3$	23166(3)	4902.78(6)	4370.44(15)	4315.73(8)
Z	20	4	2	2
$\rho_{calc}g/cm^3$	1.3	1.203	1.355	1.268
μ/mm^{-1}	1.327	6.591	1.13	6.225
F(000)	9360	1888	1854	1746
Crystal size/mm^3	0.19 × 0.16 × 0.13	0.17 × 0.04 × 0.02	0.21 × 0.18 × 0.15	0.09 × 0.06 × 0.06
Radiation	MoKα (λ = 0.71073)	CuKα (λ = 1.54184)	MoKα (λ = 0.71073)	Cu Kα (λ = 1.54184)
2Θ range for data collection/°	1.614 to 53	6.024 to 161.37	3.84 to 58	7.59 to 159.33
Index ranges	$-26 \le h \le 26$, $-27 \le k \le 27$, $-63 \le l \le 63$	$-12 \le h \le 12$, $-17 \le k \le 21$, $-36 \le l \le 35$	$-16 \le h \le 15$, $-27 \le k \le 27$, $-27 \le l \le 27$	$-15 \le h \le 15$, $-25 \le k \le 25$, $-25 \le l \le 26$
Reflections collected	658069	70032	86524	220977
Independent reflections	47979 [R_{int} = 0.0807, R_{sigma} = 0.0363]	10478 [R_{int} = 0.0483, R_{sigma} = 0.0288]	23084 [R_{int} = 0.0431, R_{sigma} = 0.0364]	18503 [R_{int} = 0.1133, R_{sigma} = 0.0413]
Data/restraints/parameters	47979/24/1952	10478/165/477	23084/426/1056	18503/768/1066
Goodness-of-fit on F^2	1.109	1.078	1.075	0.995
Final R indexes [I>=2σ (I)]	R_1 = 0.0558, wR_2 = 0.1106	R_1 = 0.0330, wR_2 = 0.0843	R_1 = 0.0226, wR_2 = 0.0494	R_1 = 0.0610, wR_2 = 0.1725
Final R indexes [all data]	R_1 = 0.0893, wR_2 = 0.1388	R_1 = 0.0363, wR_2 = 0.0859	R_1 = 0.0349, wR_2 = 0.0569	R_1 = 0.0639, wR_2 = 0.1757
Largest diff. peak/hole / e $Å^{-3}$	2.67/-5.21	0.91/-0.97	0.68/-0.63	2.24/-1.95

2. ^{1}H, ^{13}C, ^{29}Si, and ^{119}Sn NMR spectra

Compound **1:**

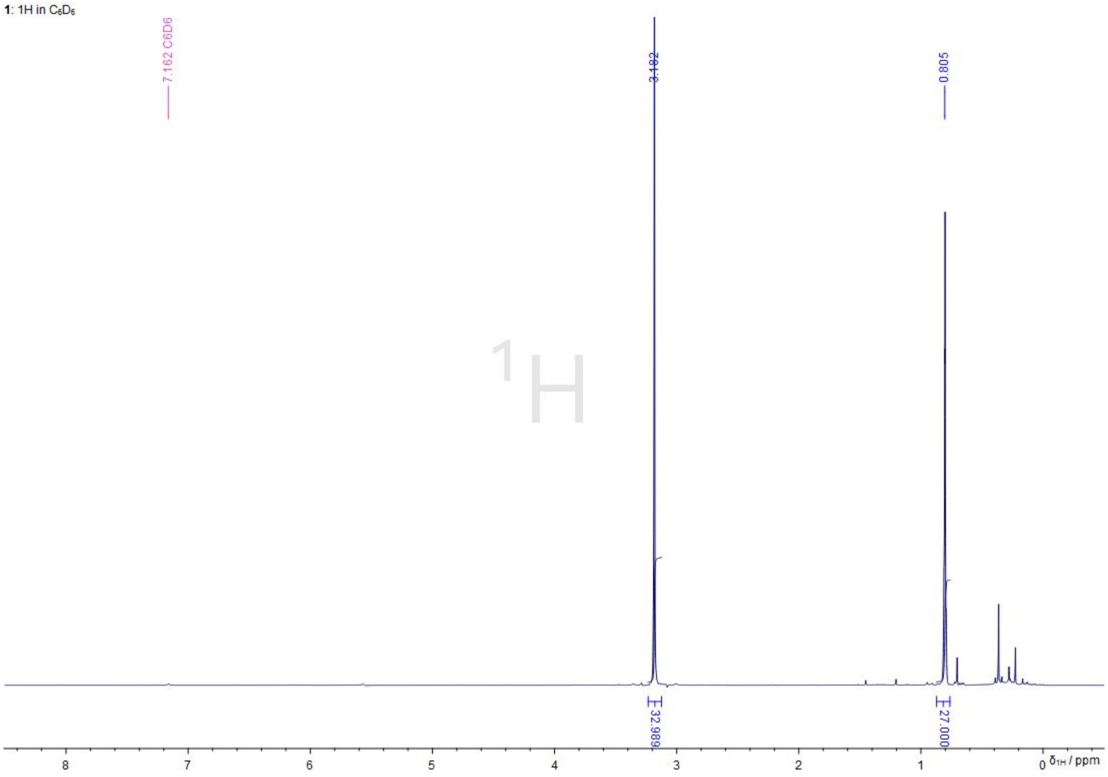

Figure S1. ^{1}H-NMR spectrum of **1** in C_6D_6

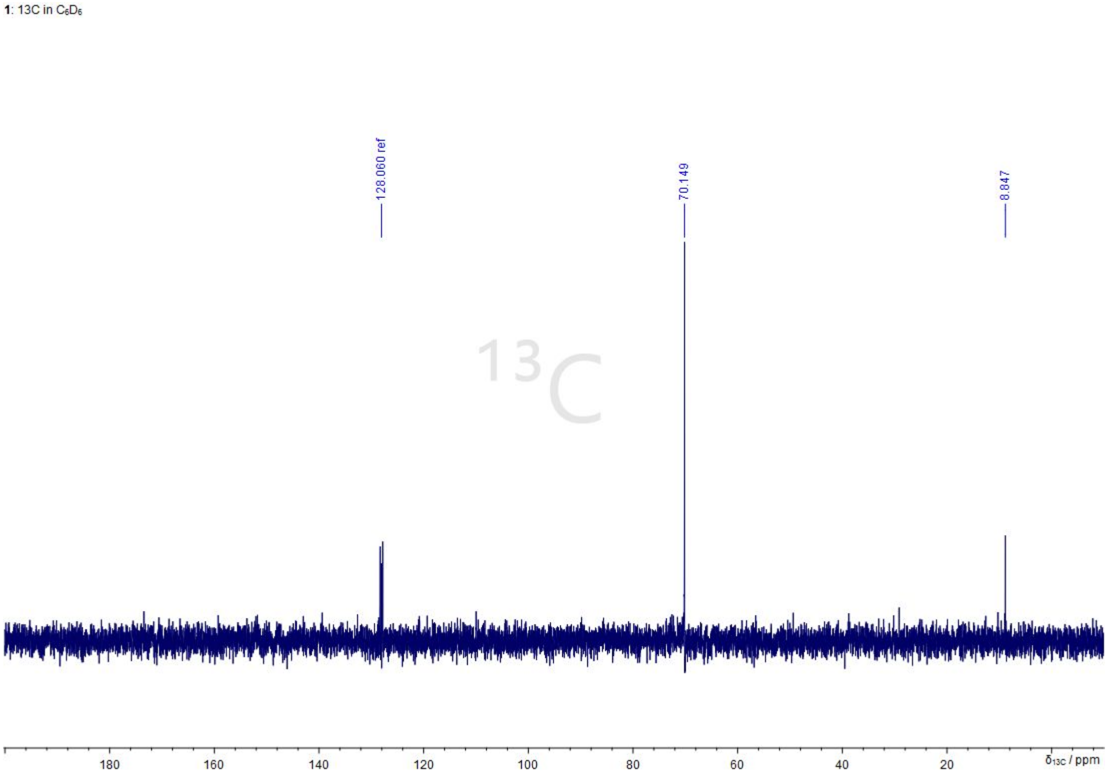

Figure S2. ^{13}C{H}-NMR spectrum of **1** in C_6D_6

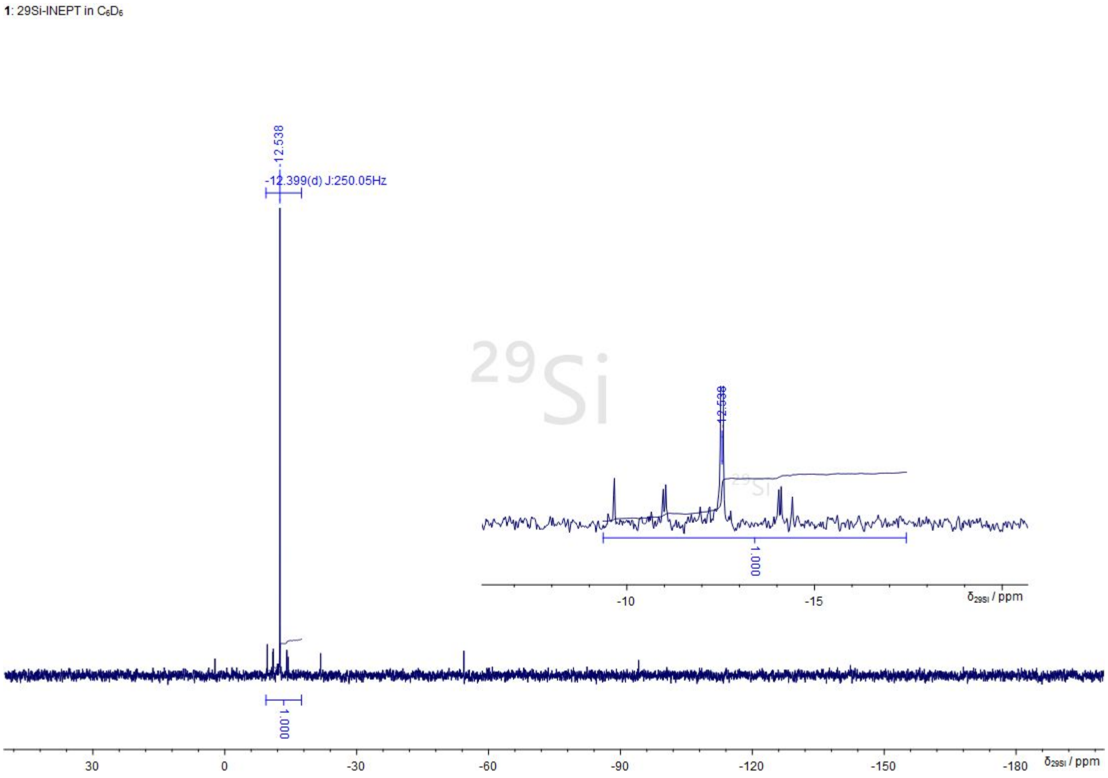

Figure S3. ^{29}Si{H}-INEPT-NMR spectrum of **1** in C_6D_6

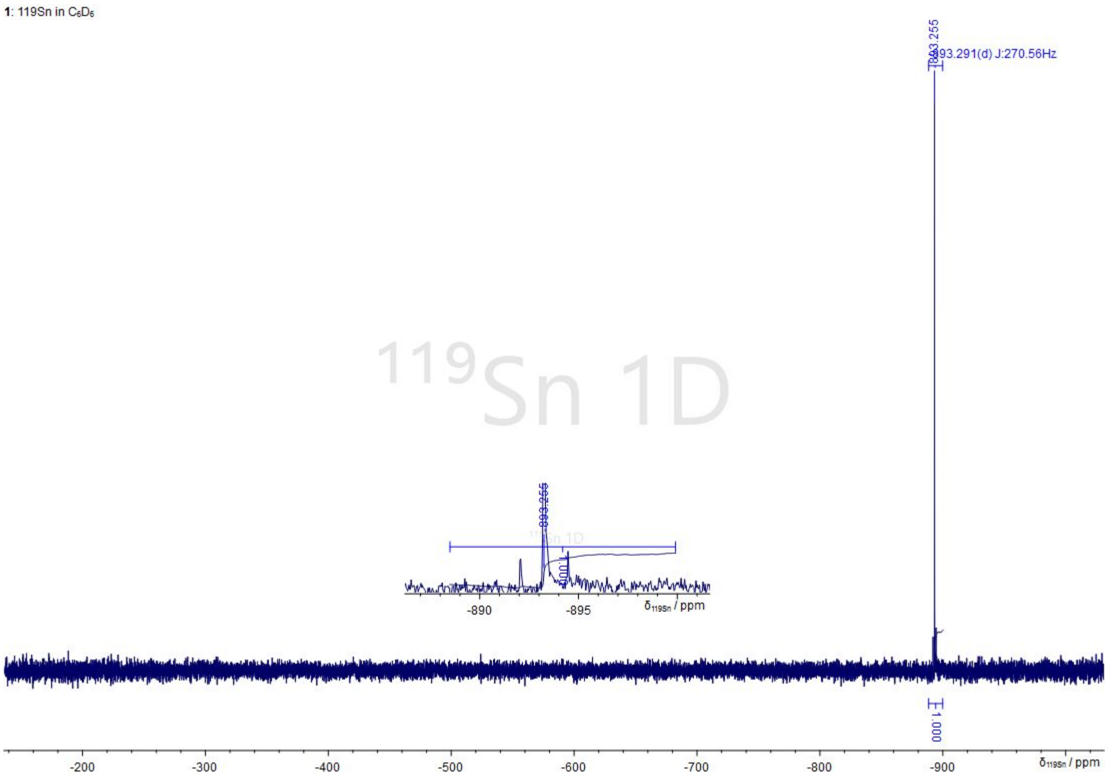

Figure S4. ^{119}Sn{H}-NMR spectrum of **1** in C_6D_6

Compound **2**:

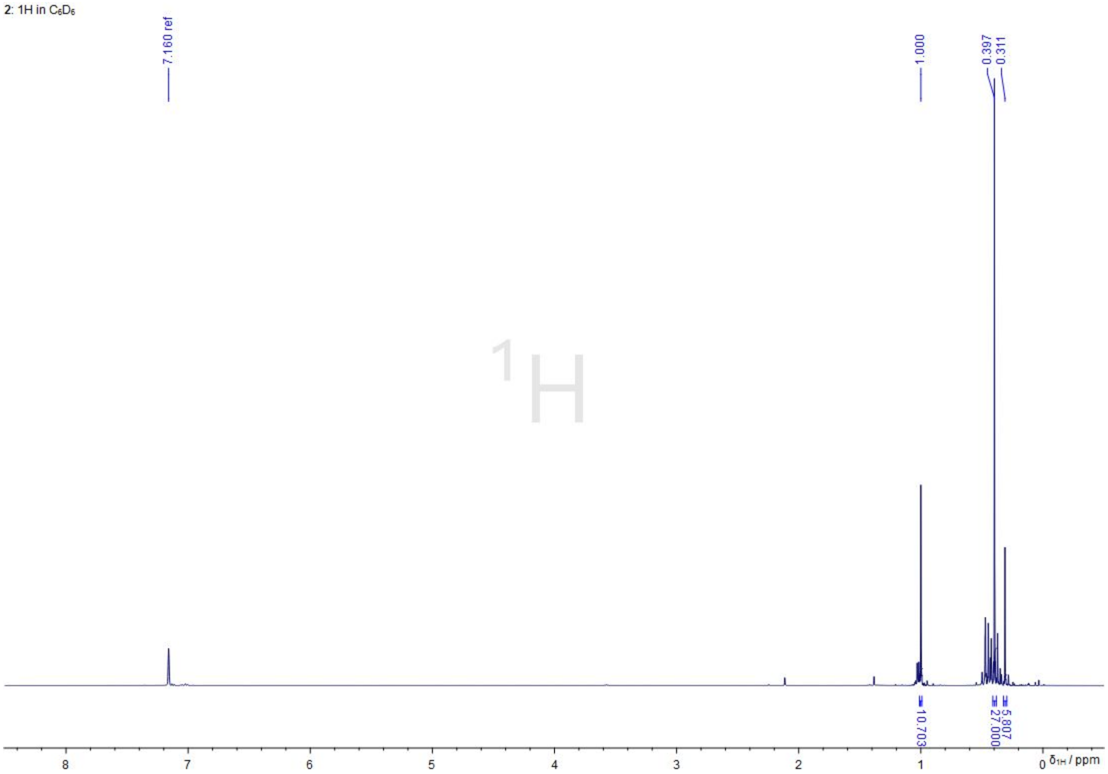

Figure S5. ^{1}H-NMR spectrum of **2** in C_6D_6

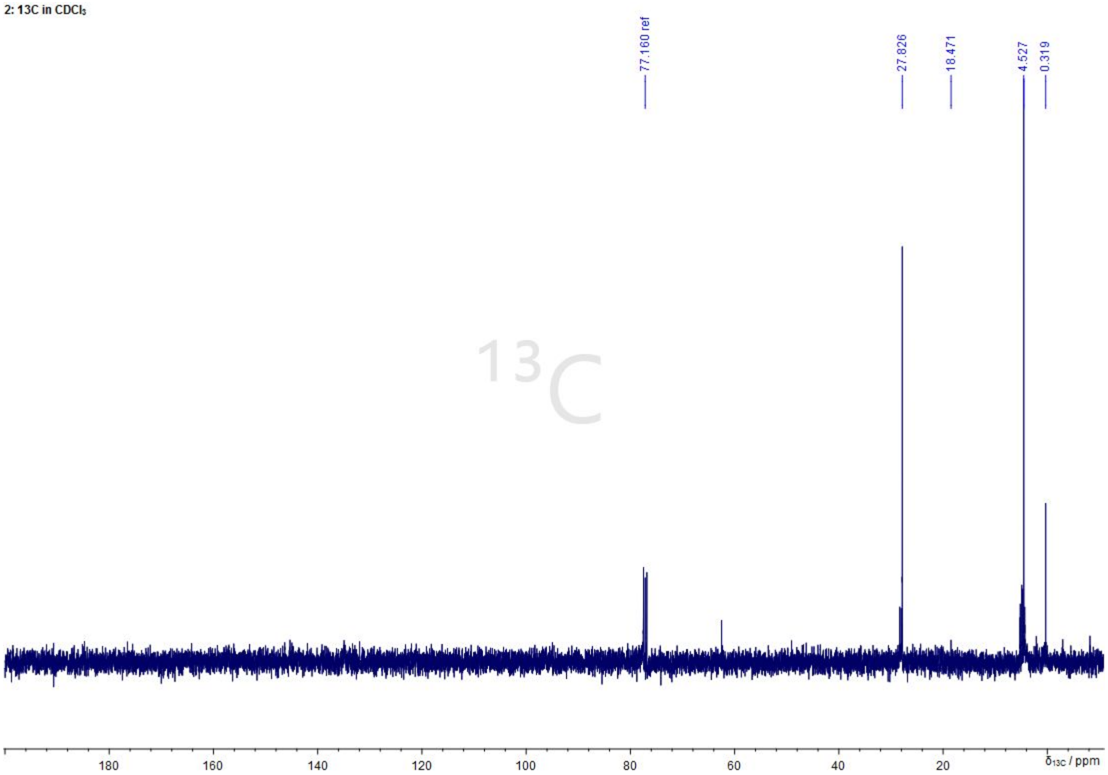

Figure S6. $^{13}C\{H\}$-NMR spectrum of **2** in $CDCl_3$

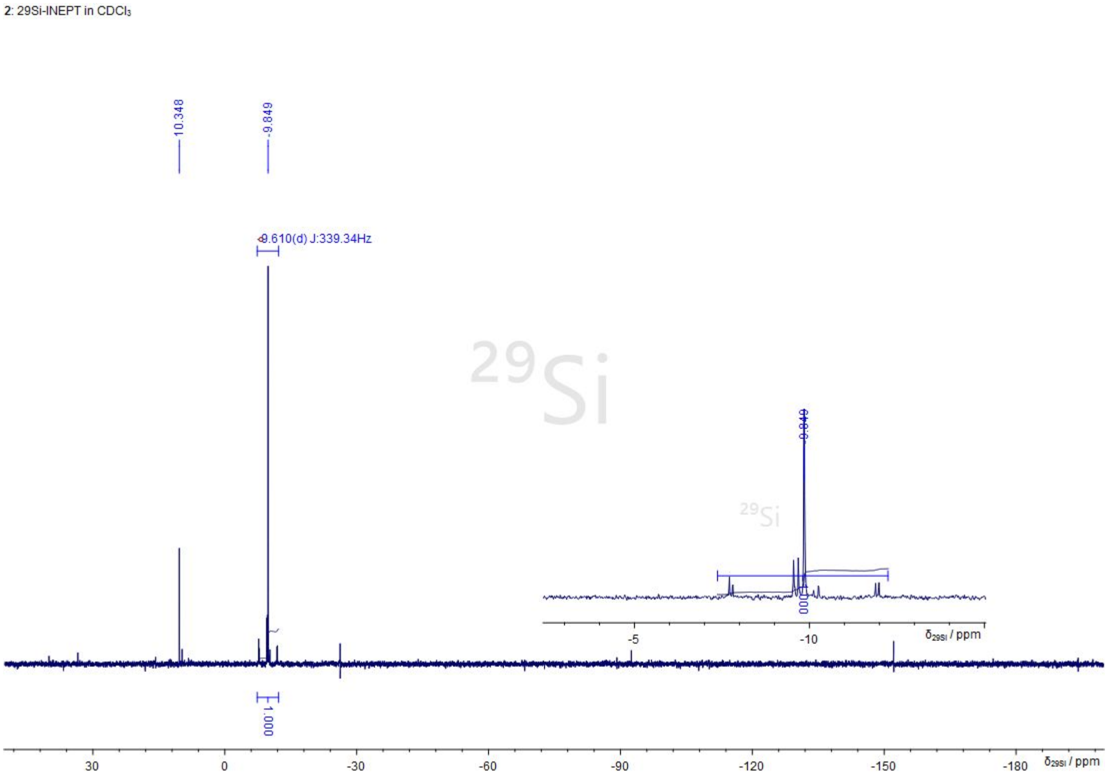

Figure S7. $^{29}Si\{H\}$-INEPT-NMR spectrum of **2** in $CDCl_3$

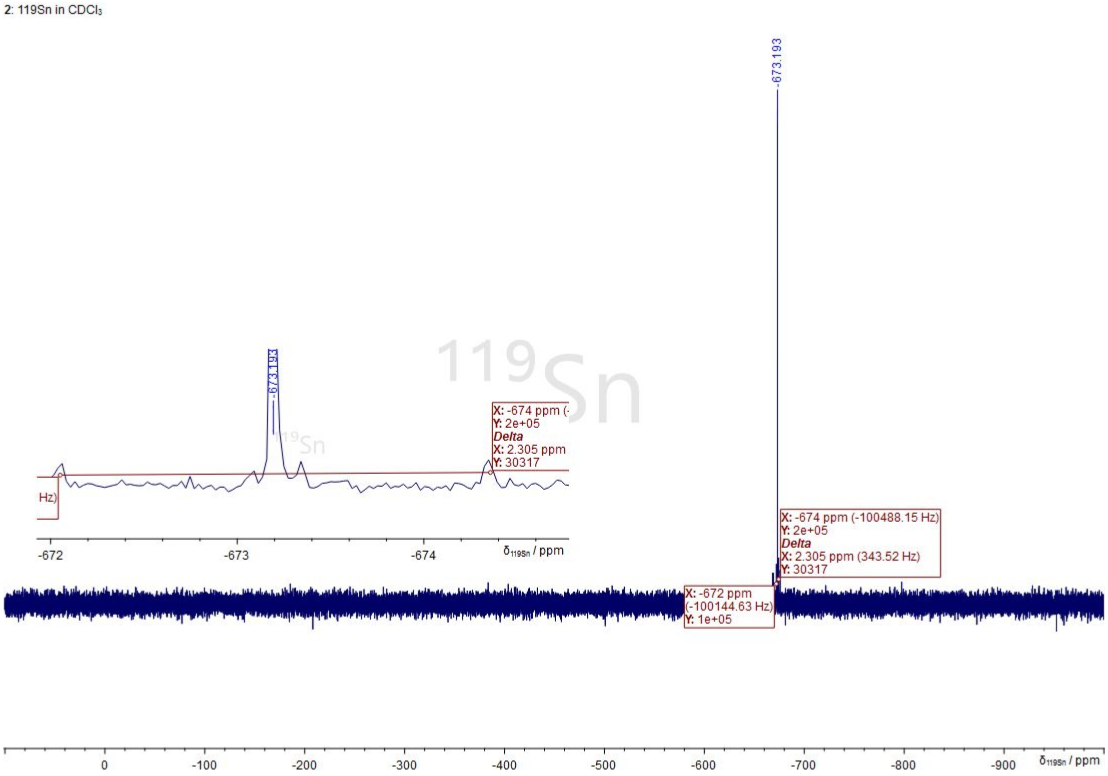

Figure S8. $^{119}Sn\{H\}$-NMR spectrum of **2** in C_6D_6

Compound **3**:

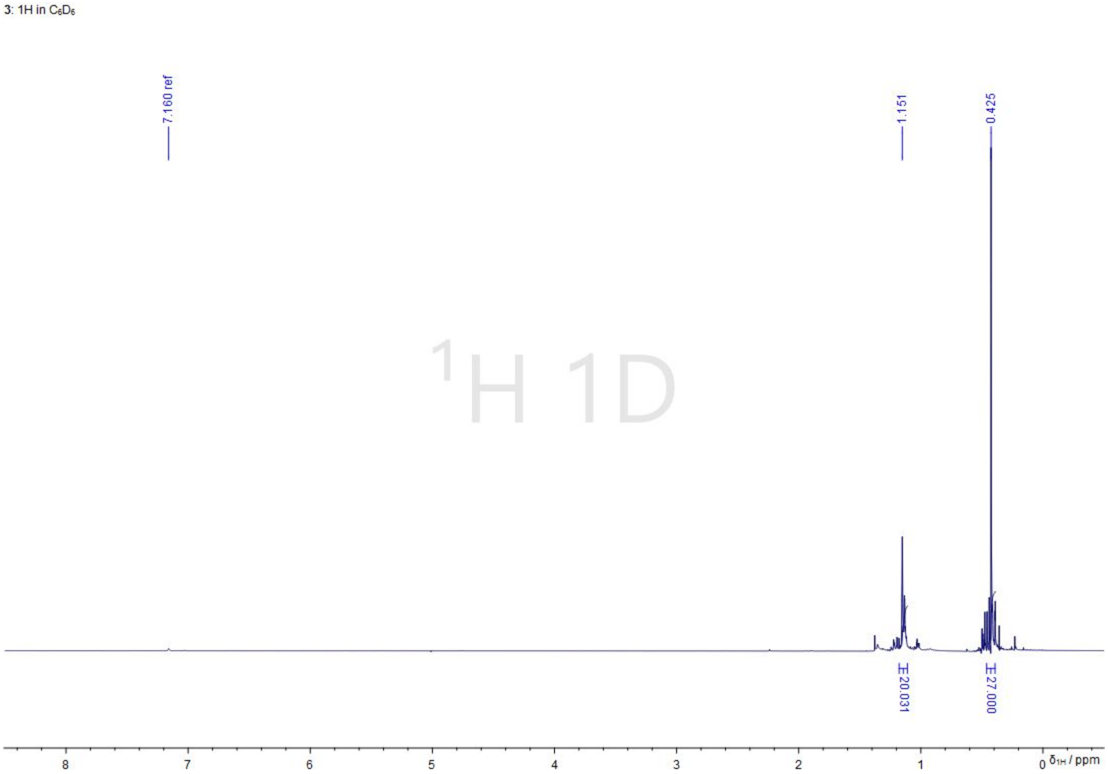

Figure S9. ^{1}H-NMR spectrum of **3** in C_6D_6

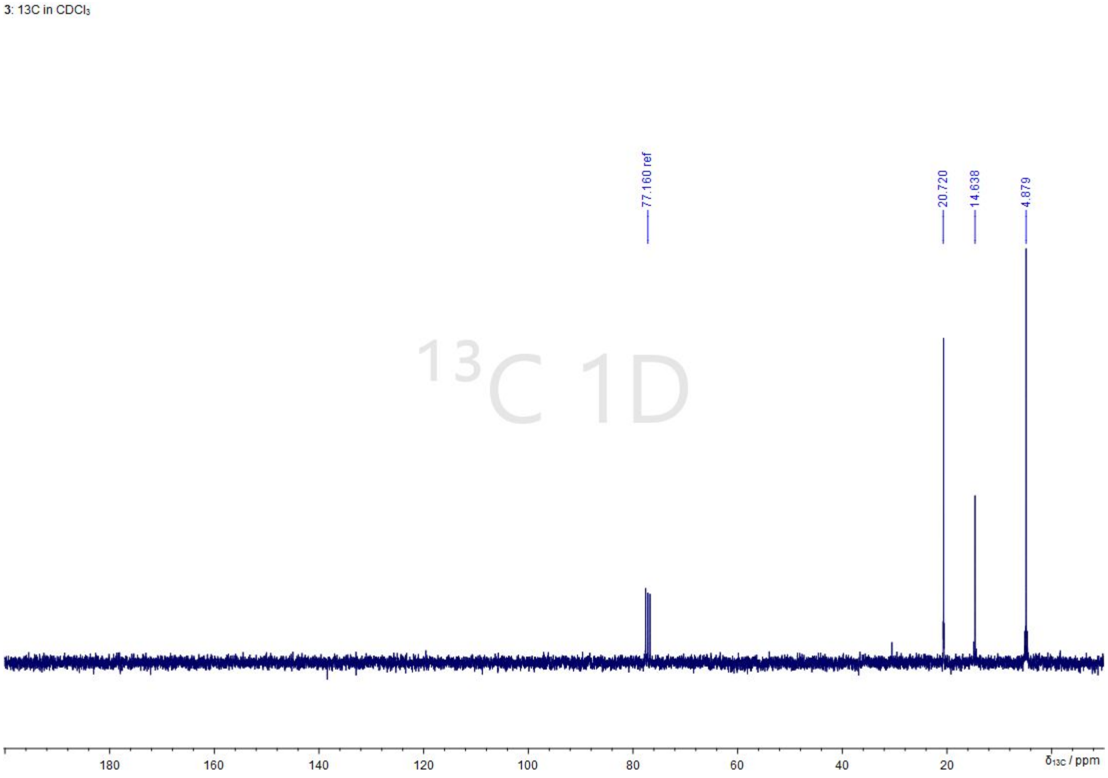

Figure S10. ^{13}C{H}-NMR spectrum of **3** in $CDCl_3$

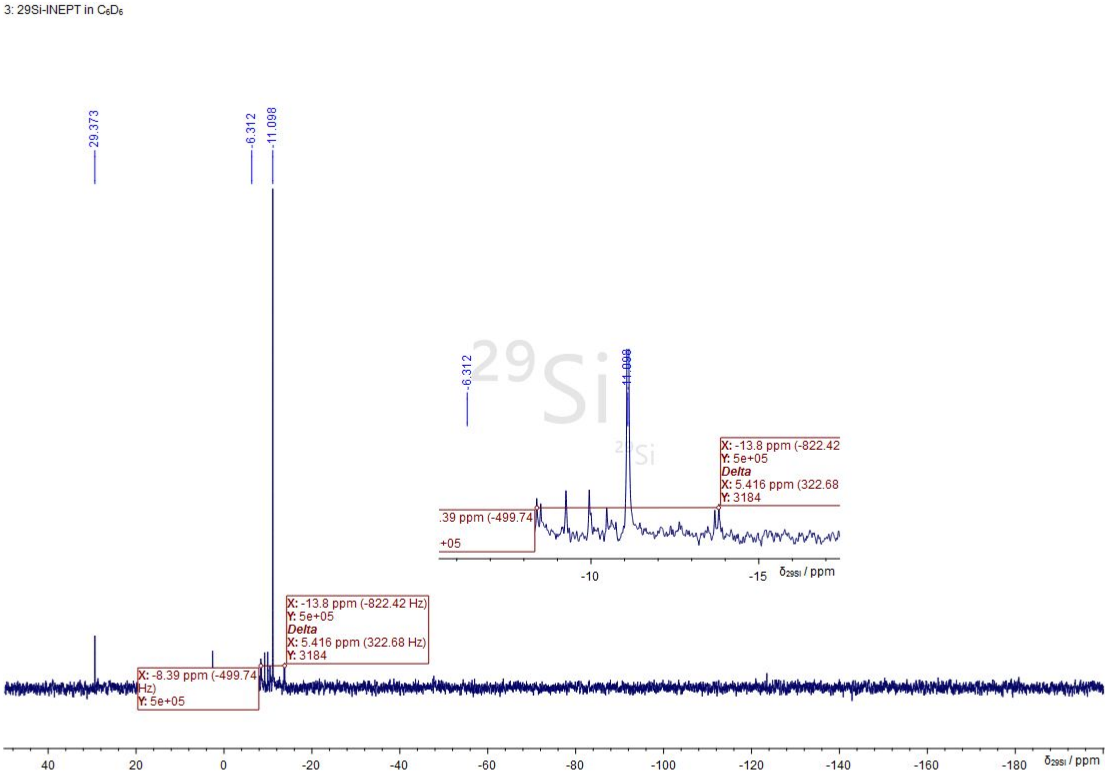

Figure S11. ^{29}Si{H}-INEPT-NMR spectrum of **3** in C_6D_6

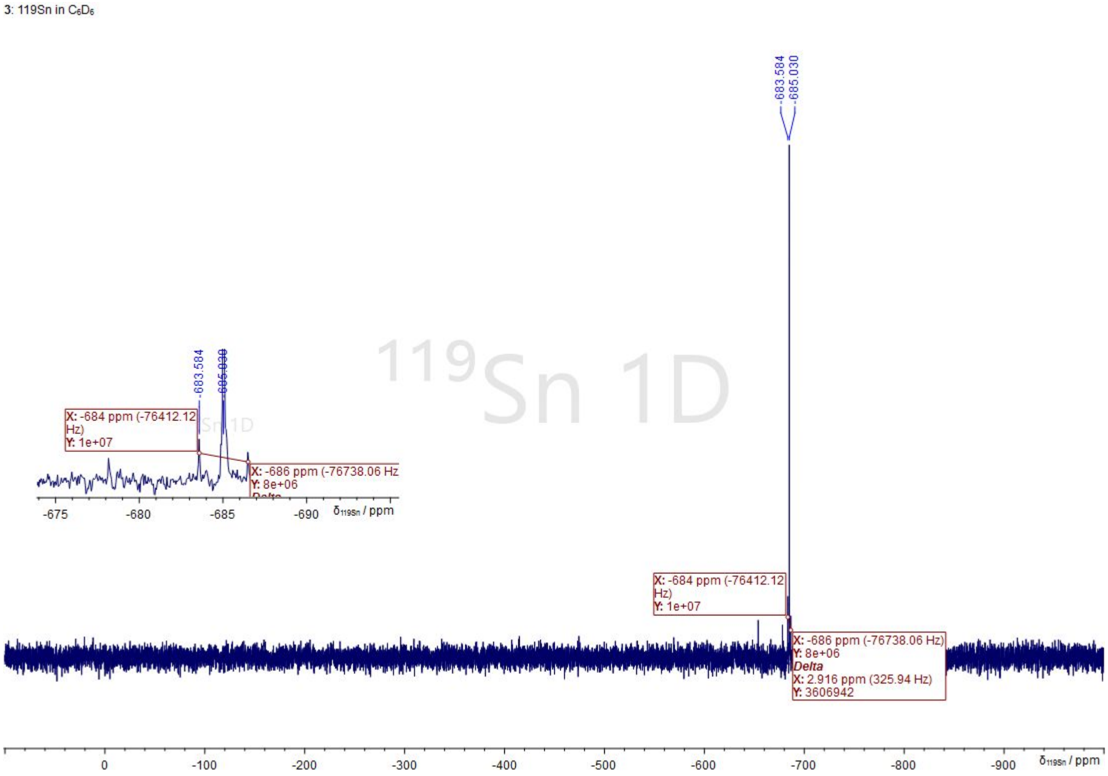

Figure S12. ^{119}Sn{H}-NMR spectrum of **3** in $CDCl_3$

Compound **4**:

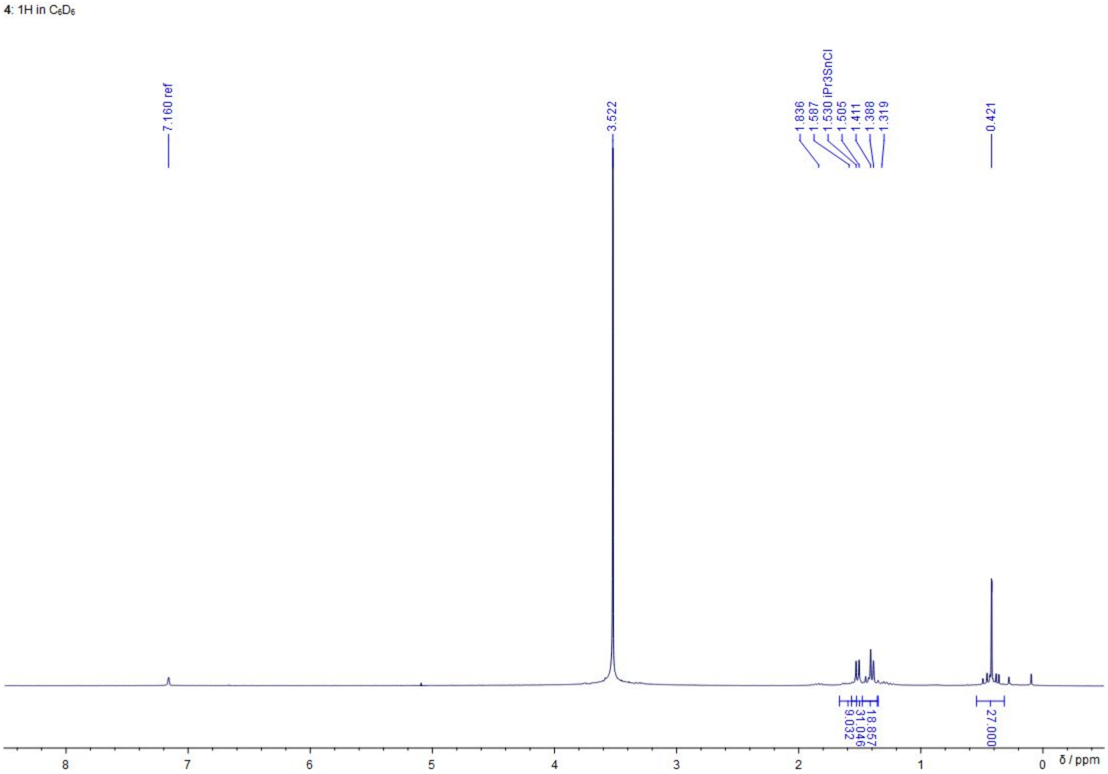

Figure S13. ^{1}H-NMR spectrum of **4** in C_6D_6

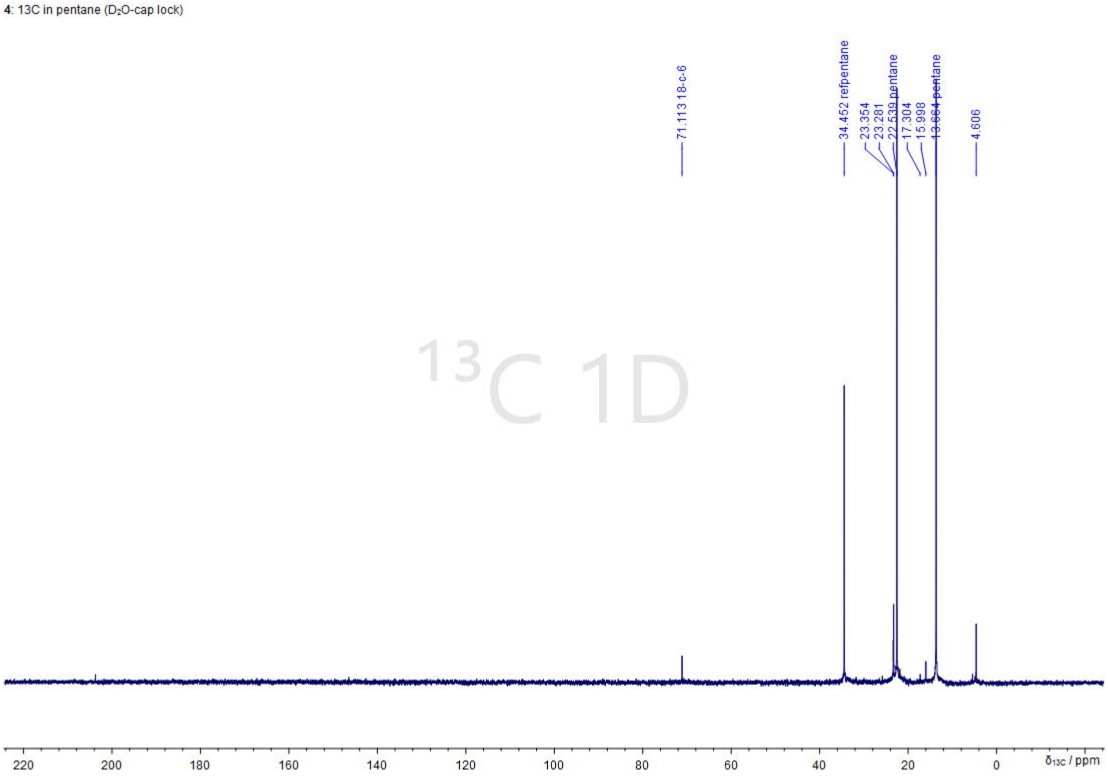

Figure S14. $^{13}C\{H\}$-NMR spectrum of **4** in pentane (D_2O-cap lock)

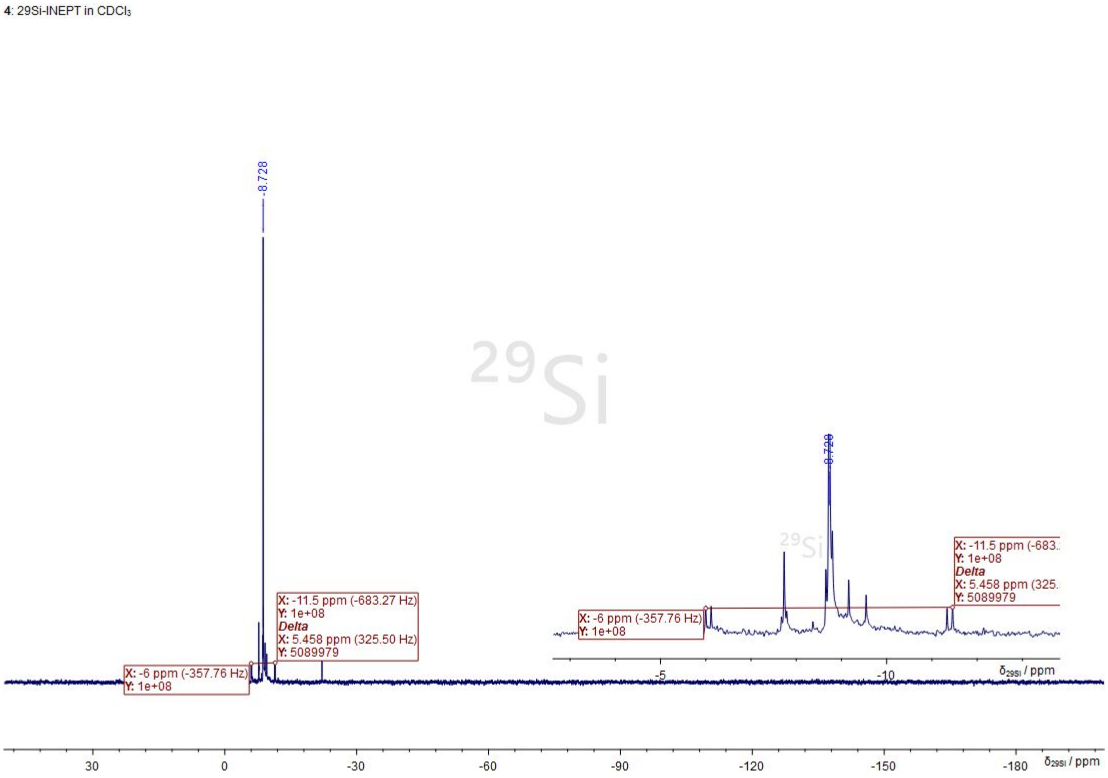

Figure S15. $^{29}Si\{H\}$-INEPT-NMR spectrum of **4** in $CDCl_3$

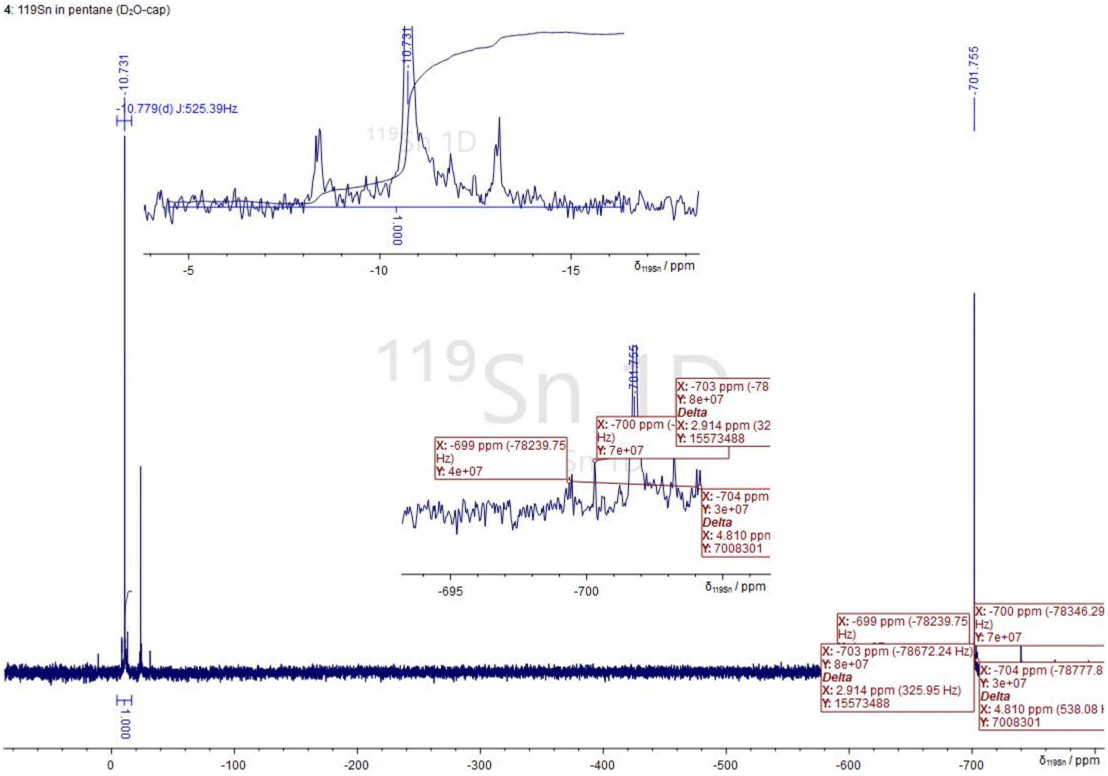

Figure S16. $^{119}Sn\{H\}$-NMR spectrum of **4** in pentane (D_2O-cap lock)

Compound **5**:

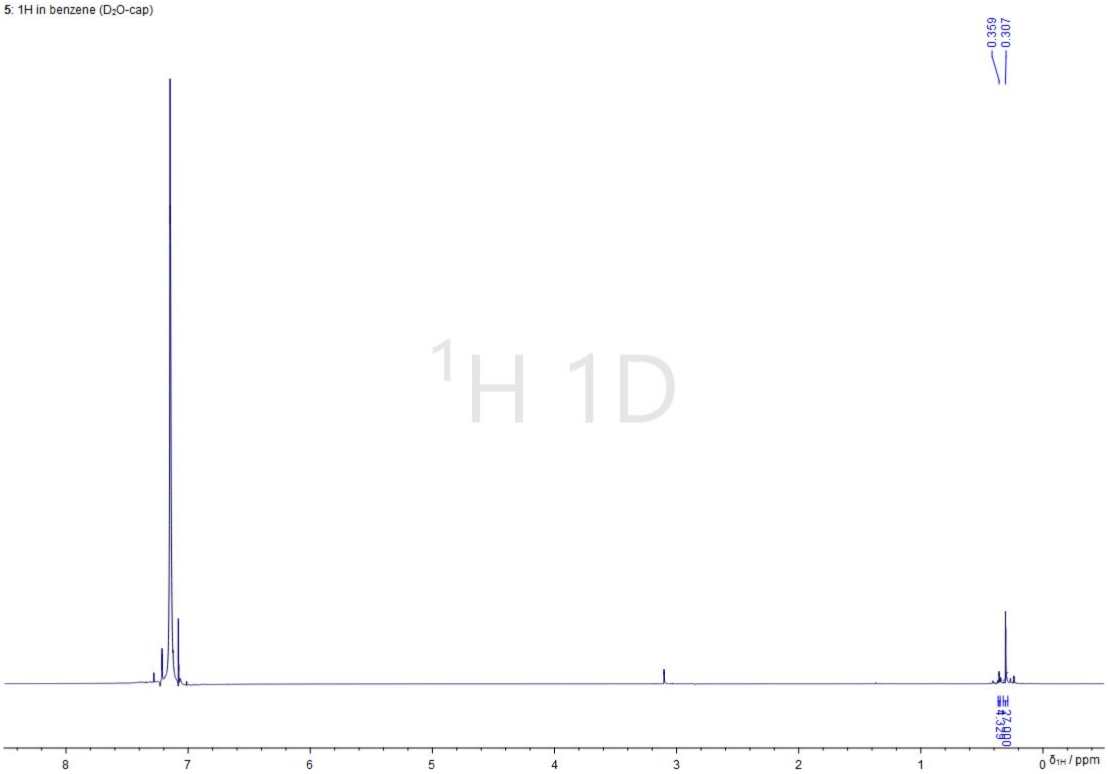

Figure S17. ^{1}H-NMR spectrum of **5** in benzene (D_2O cap lock)

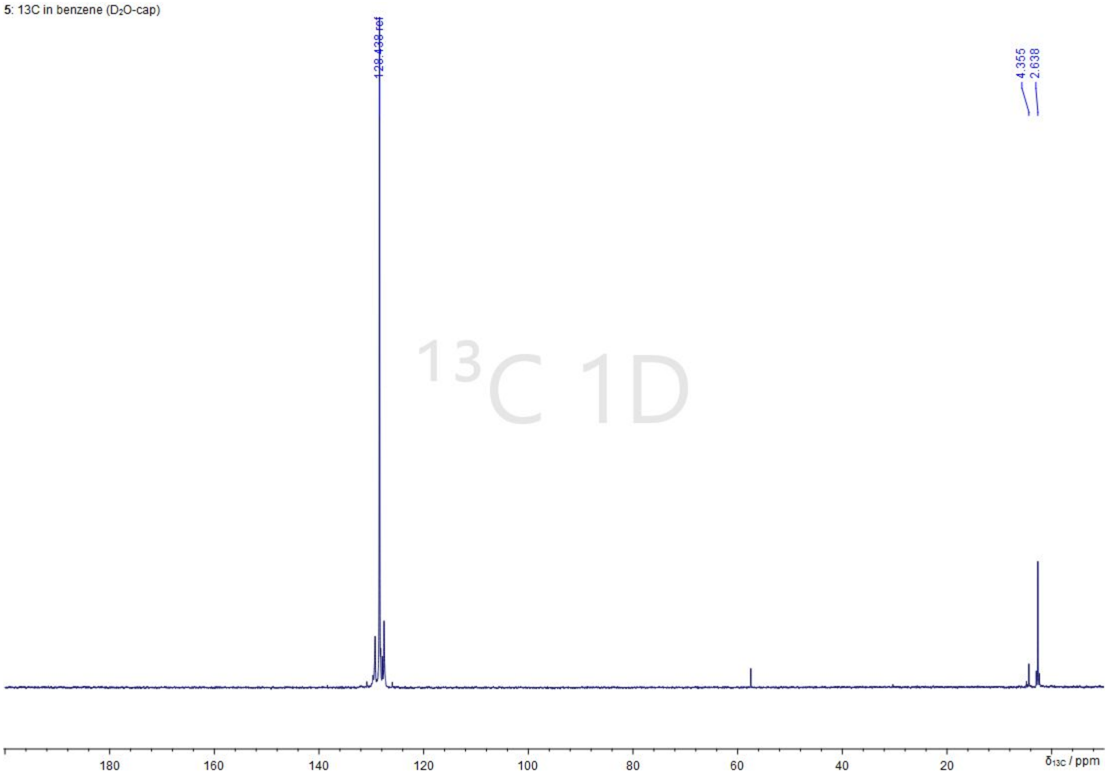

Figure S18. ^{13}C{H}-NMR spectrum of **5** benzene (D_2O cap lock)

5: 29Si-INEPT in pentane (D_2O-cap)

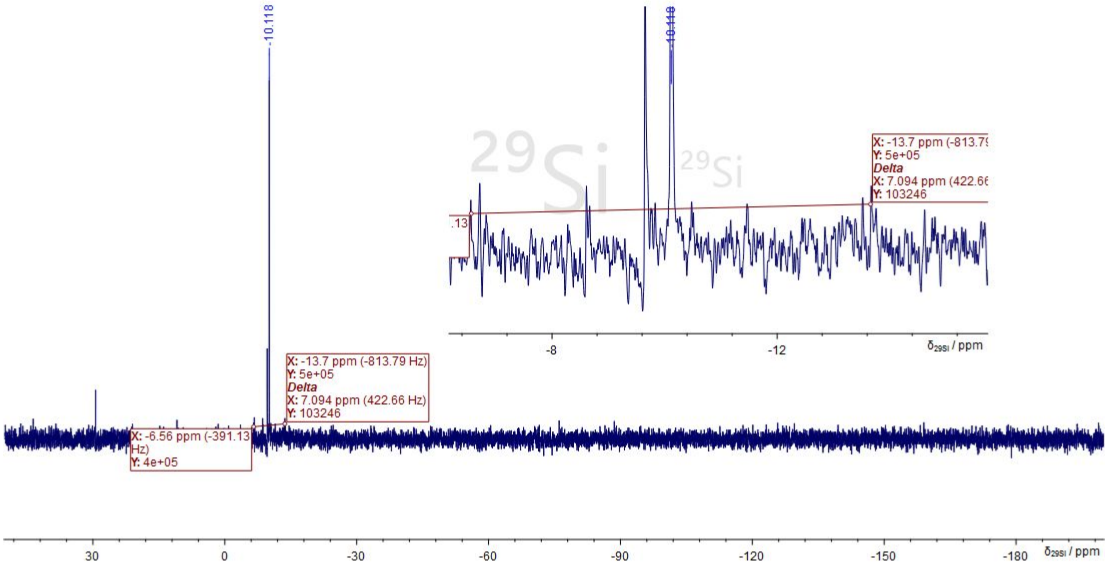

Figure S19. ^{29}Si{H}-INEPT-NMR spectrum of **5** in pentane (D_2O-cap lock)

5: 119Sn in pentane (D_2O-cap)

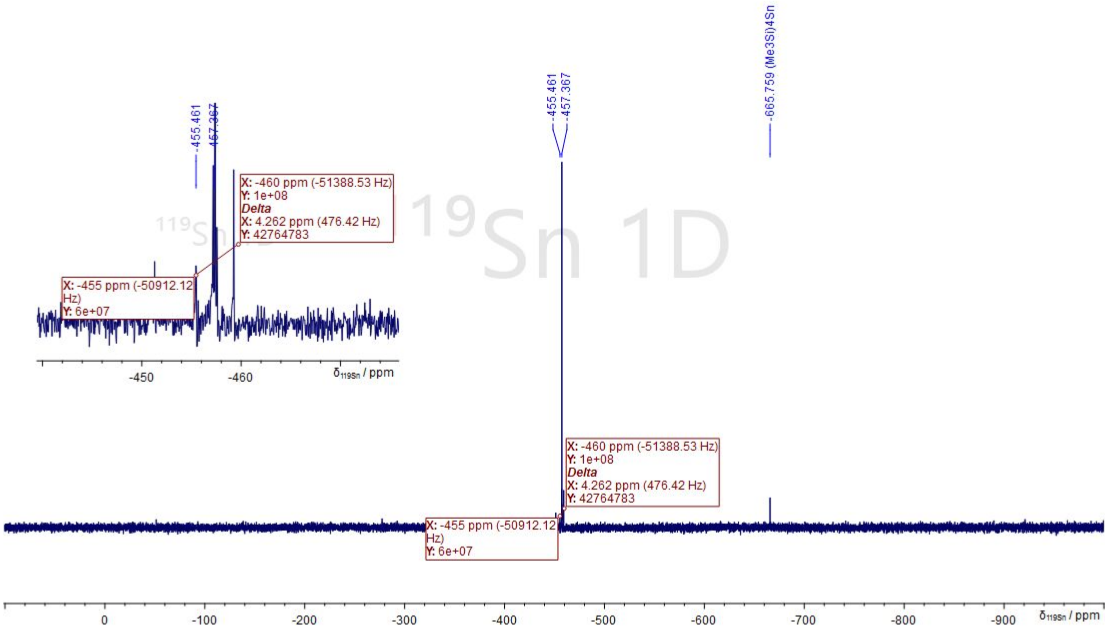

Figure S20. ^{119}Sn{H}-NMR spectrum of **5** in pentane (D_2O-cap lock)

Compound **6**:

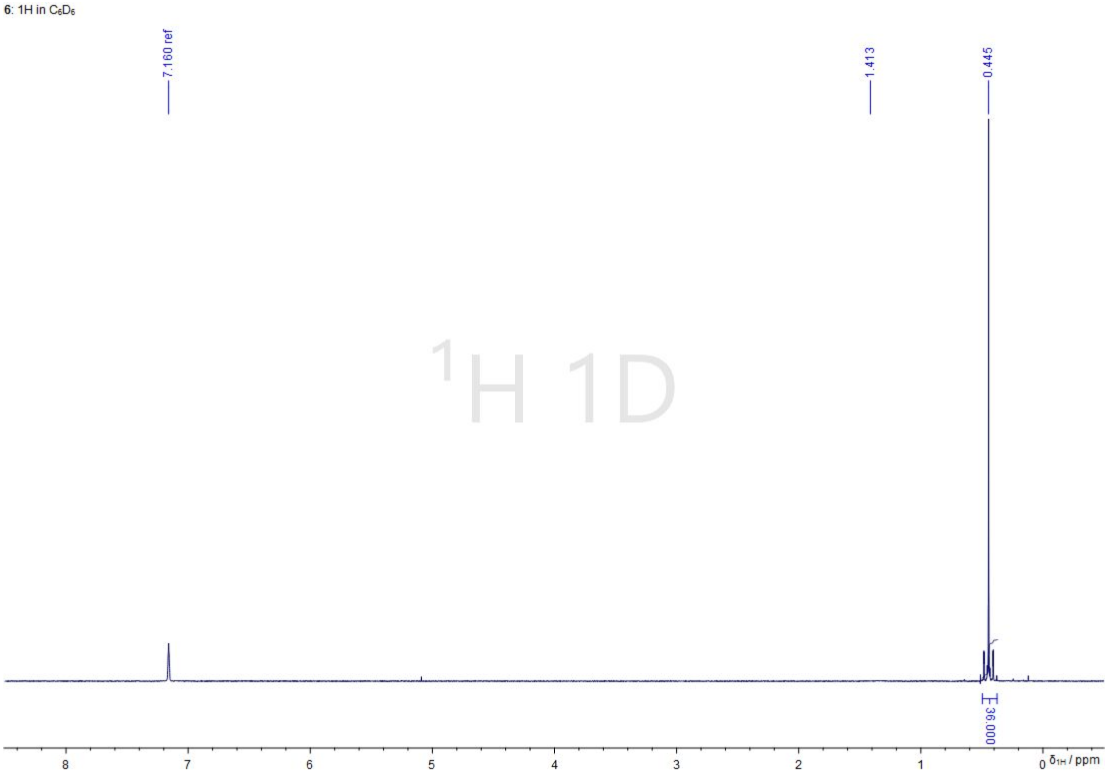

Figure S21. ^{1}H-NMR spectrum of **6** in C_6D_6

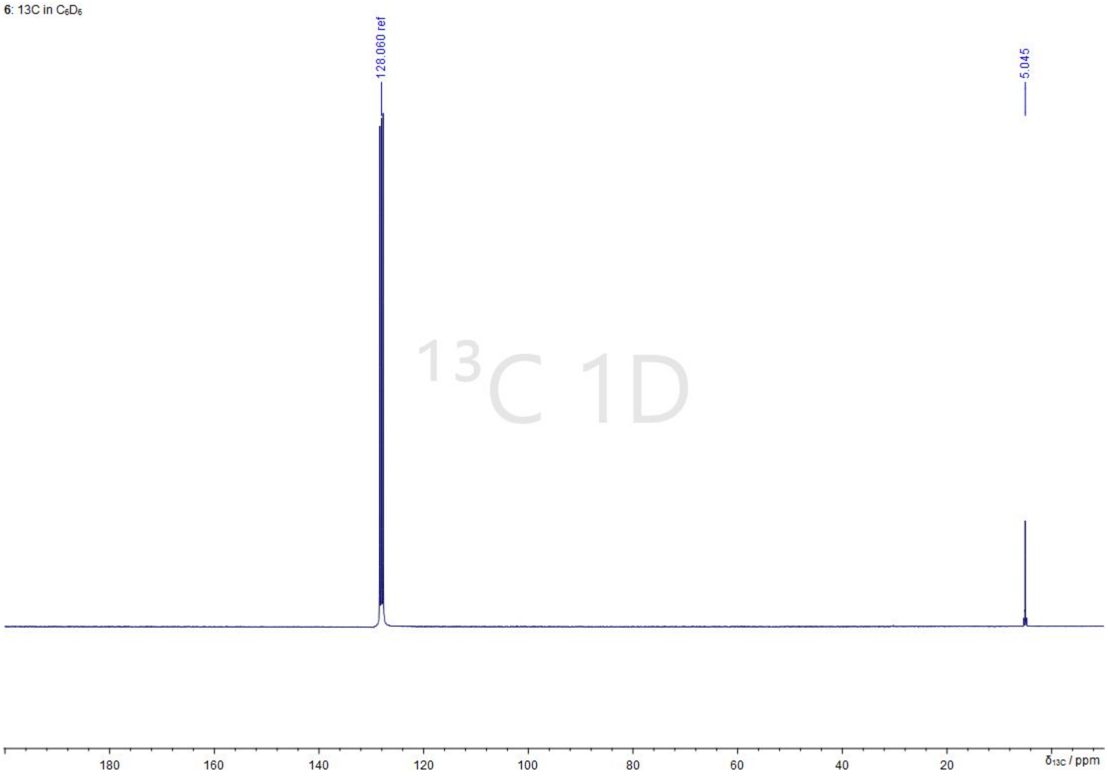

Figure S22. ^{13}C{H}-NMR spectrum of **6** in C_6D_6

6: 29Si-INEPT in C_6D_6

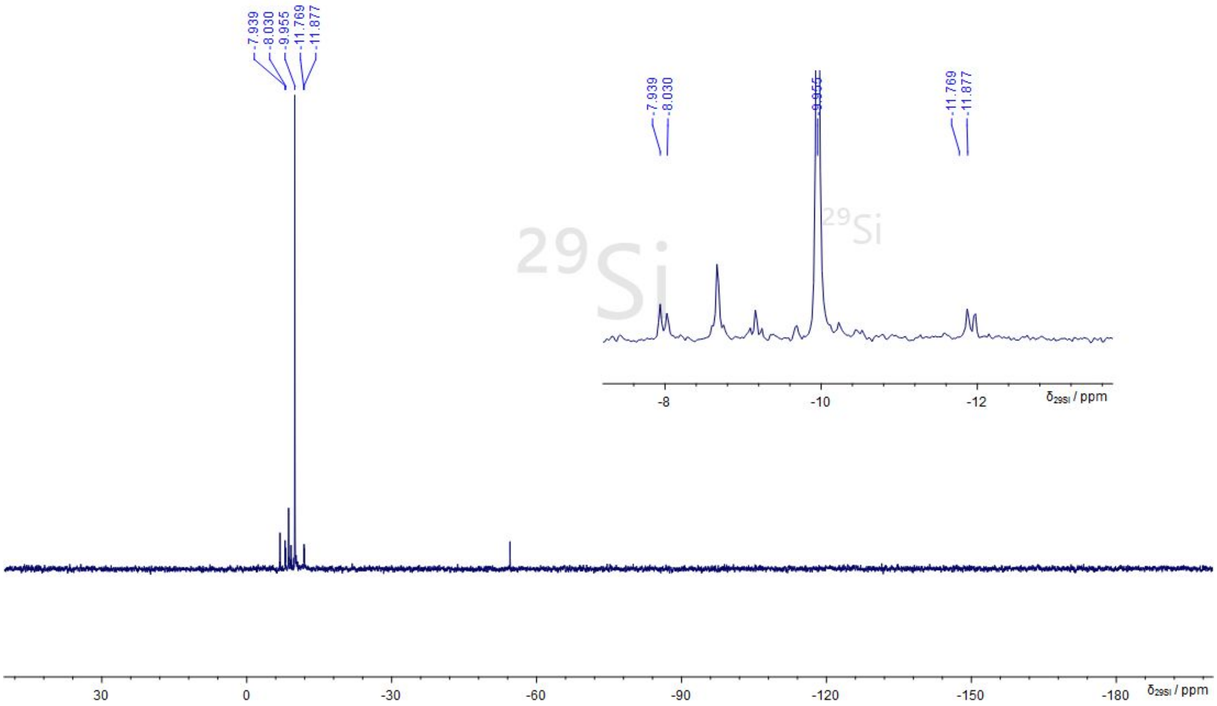

Figure S23. $^{29}Si\{H\}$-INEPT-NMR spectrum of **6** in C_6D_6

6: 119Sn in C6>D6

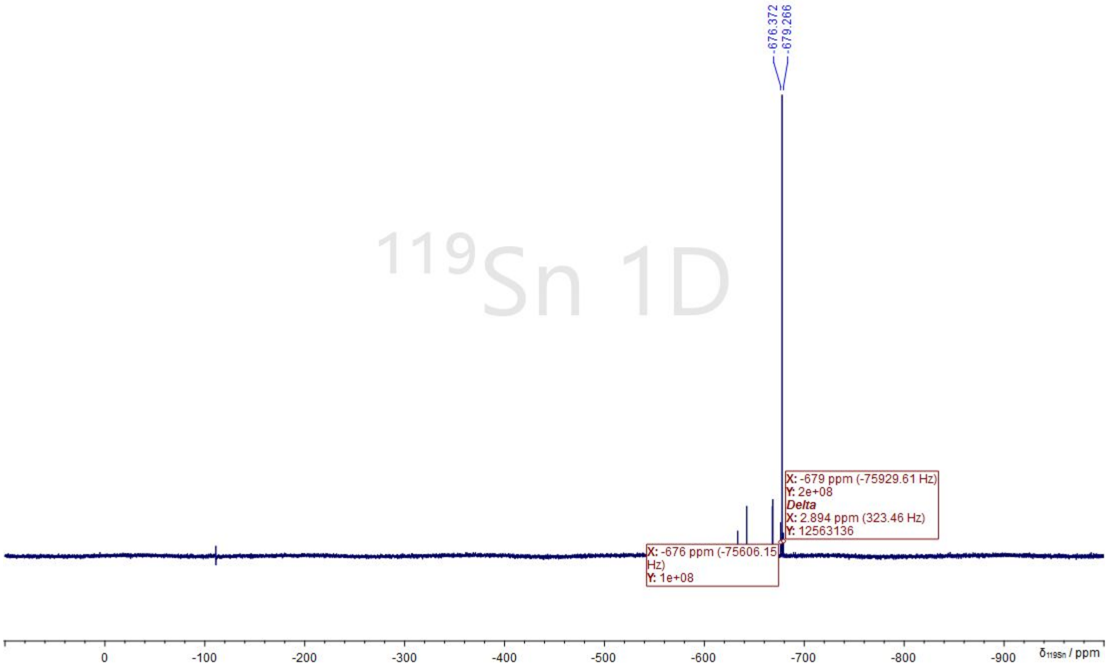

Figure S24. $^{119}Sn\{H\}$-NMR spectrum of **6** in C_6D_6

Compound **7**:

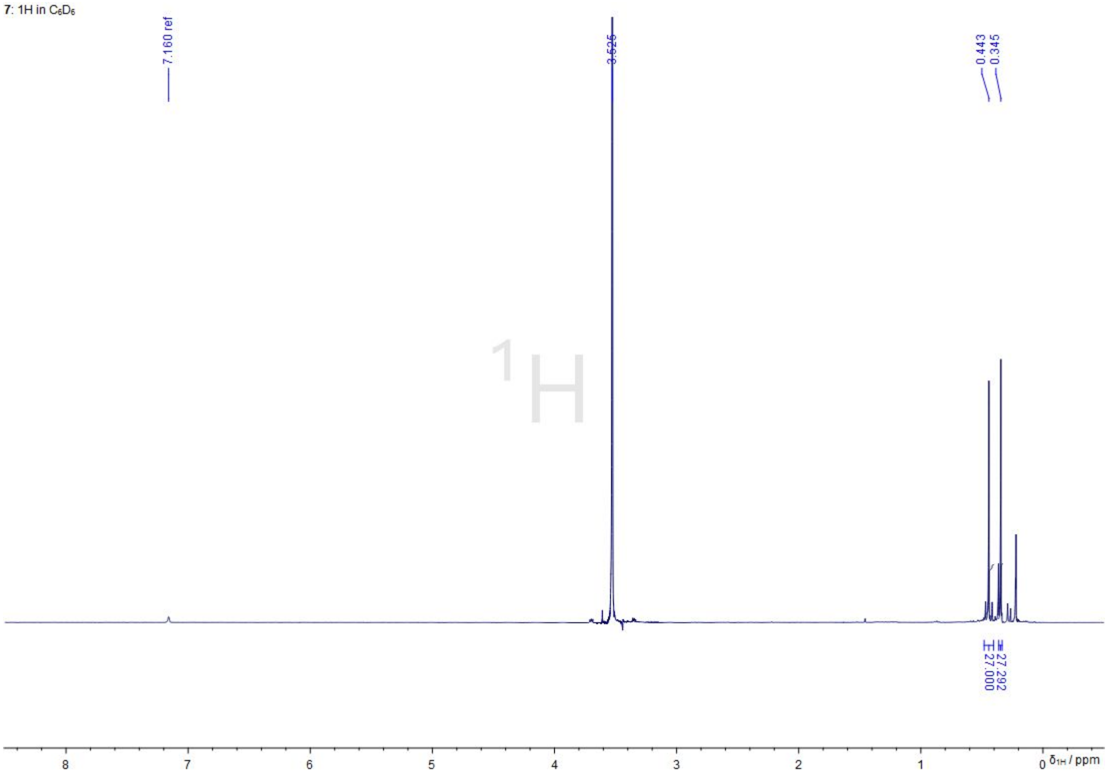

Figure S25. ^{1}H-NMR spectrum of **7** in C_6D_6

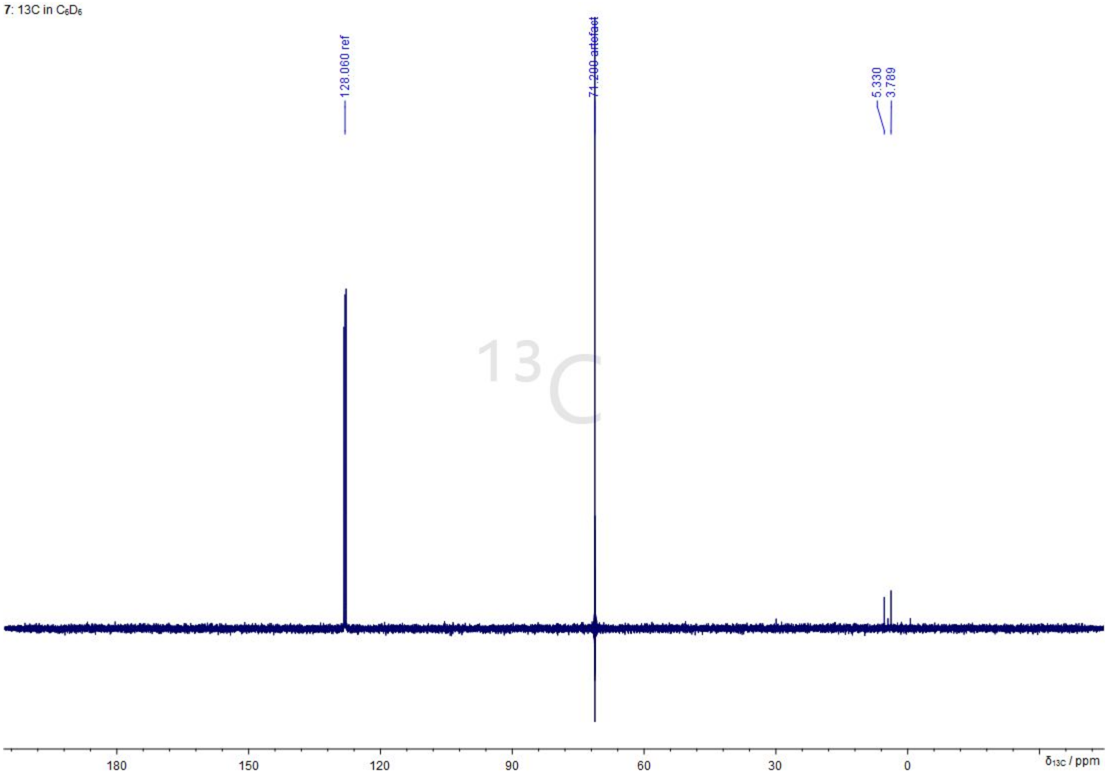

Figure S26. ^{13}C{H}-NMR spectrum of **7** in C_6D_6

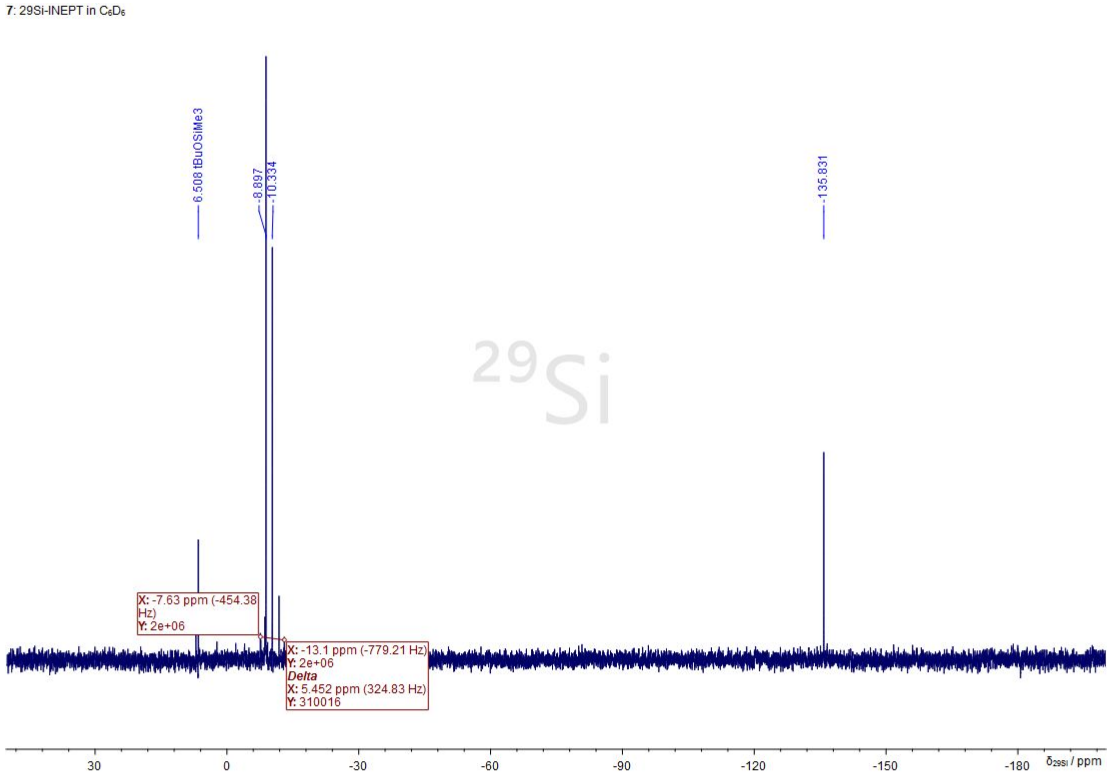

Figure S27. $^{29}Si\{H\}$-INEPT-NMR spectrum of **7** in C_6D_6

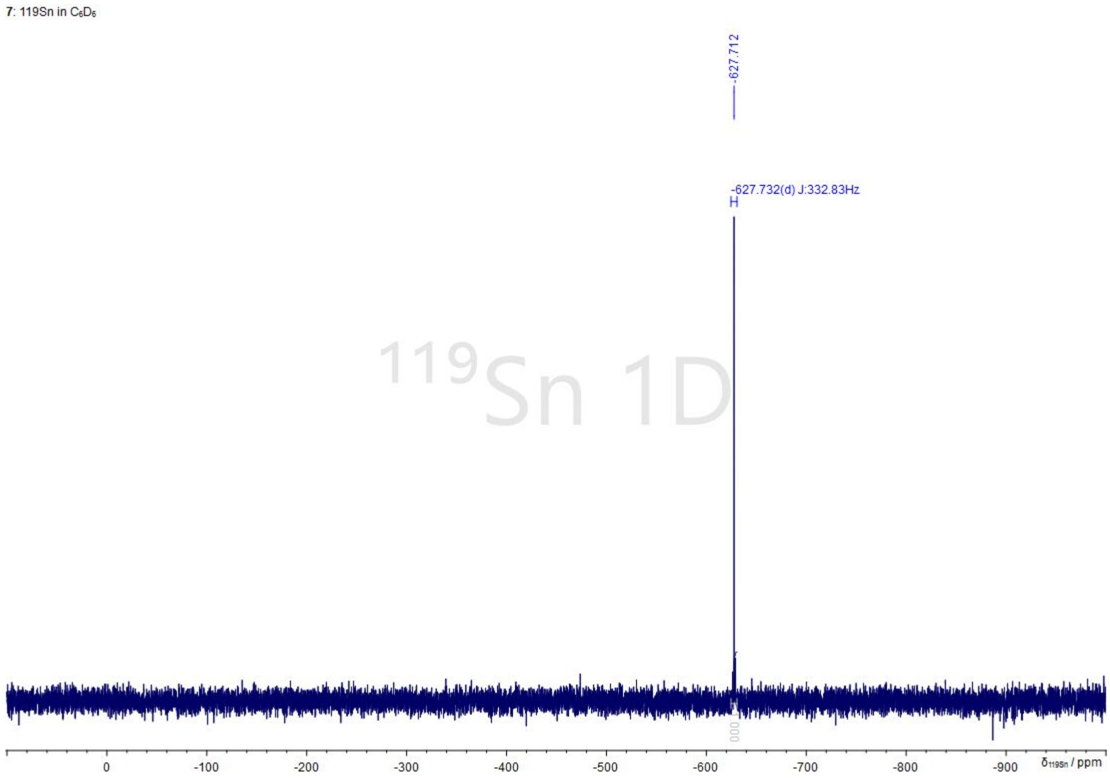

Figure S28. $^{119}Sn\{H\}$-NMR spectrum of **7** in C_6D_6

Compound **8**: Ge

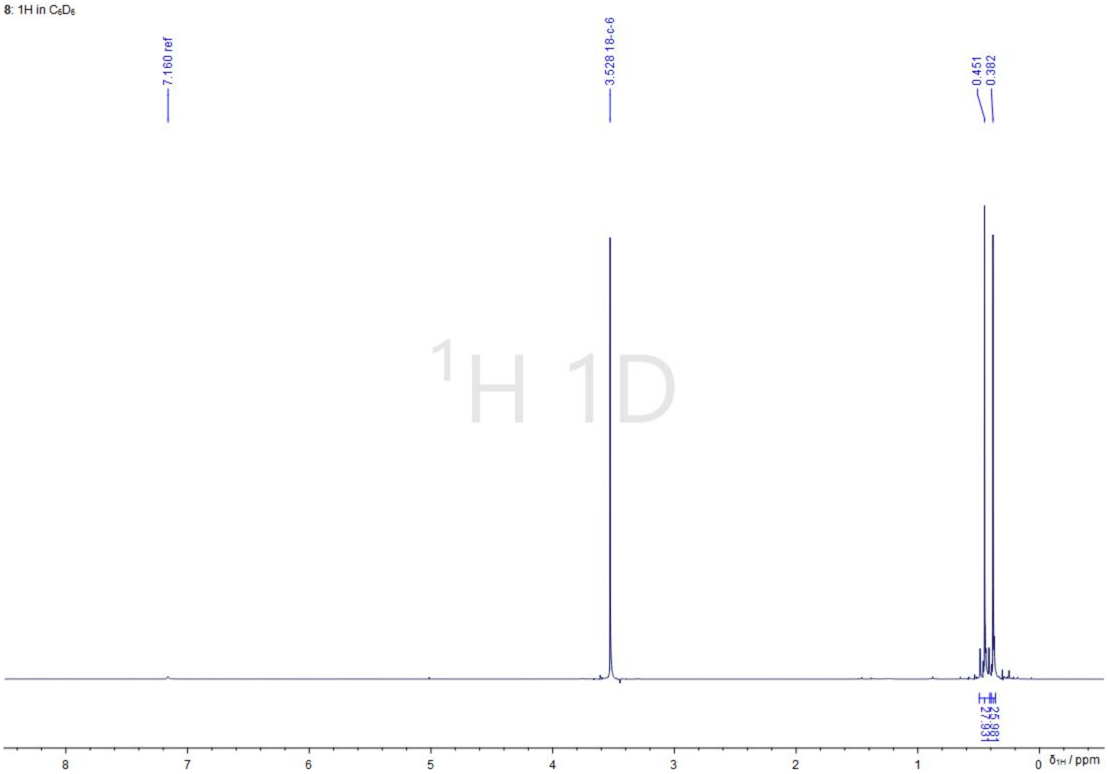

Figure S29. ^{1}H-NMR spectrum of **8** in C_6D_6

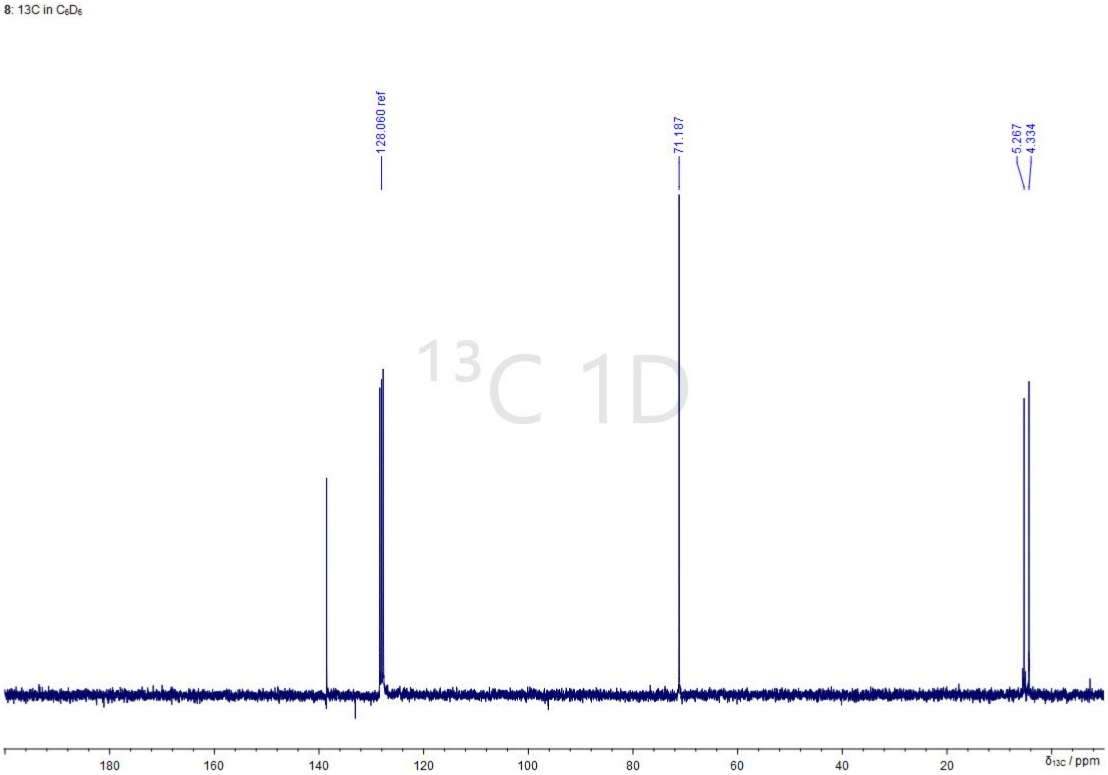

Figure S30. ^{13}C{H}-NMR spectrum of **8** in C_6D_6

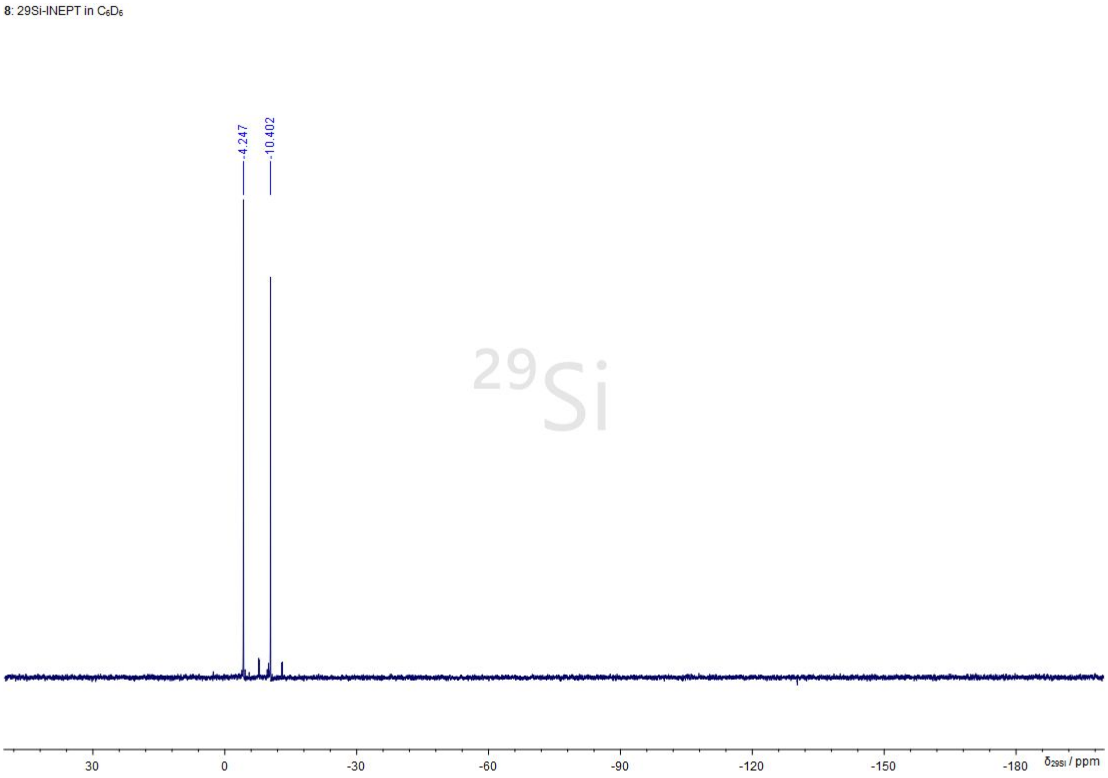

Figure S31. $^{29}Si\{H\}$-INEPT-NMR spectrum of **8** in C_6D_6

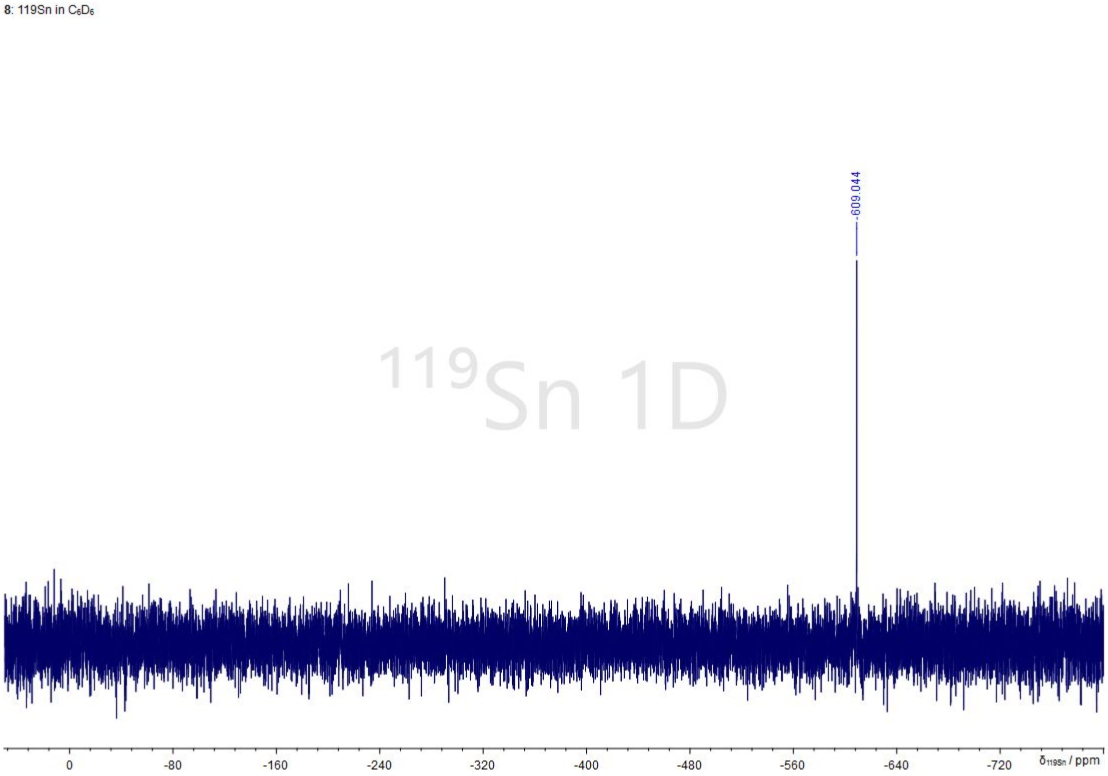

Figure S32. $^{119}Sn\{H\}$-NMR spectrum of **8** in C_6D_6

Compound **11**:

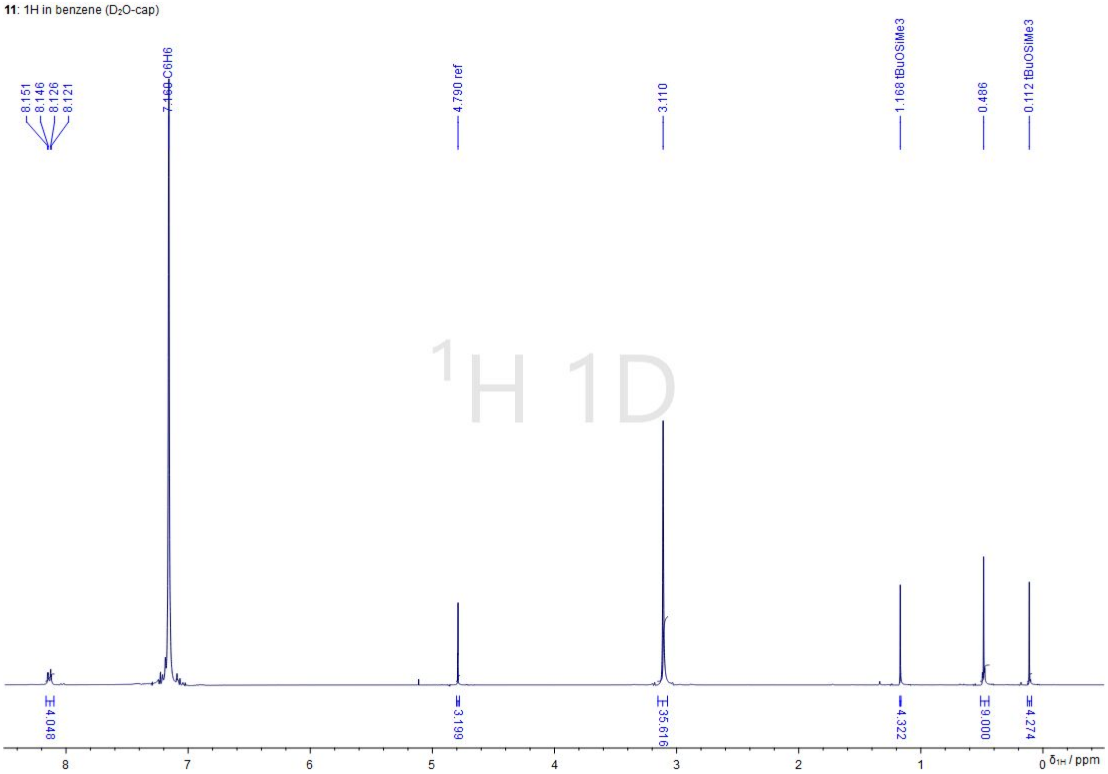

Figure S33. ^{1}H-NMR spectrum of **11** in benzene (D_2O-cap lock)

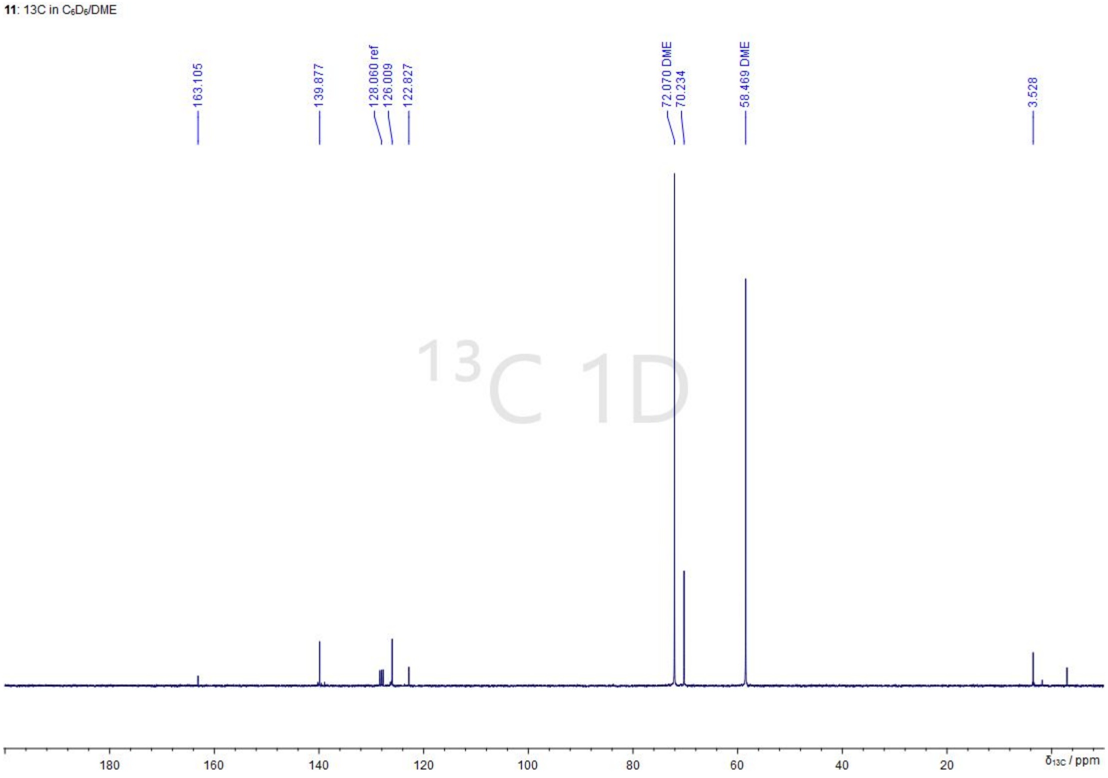

Figure S34. ^{13}C{H}-NMR spectrum of **11** in C_6D_6/DME

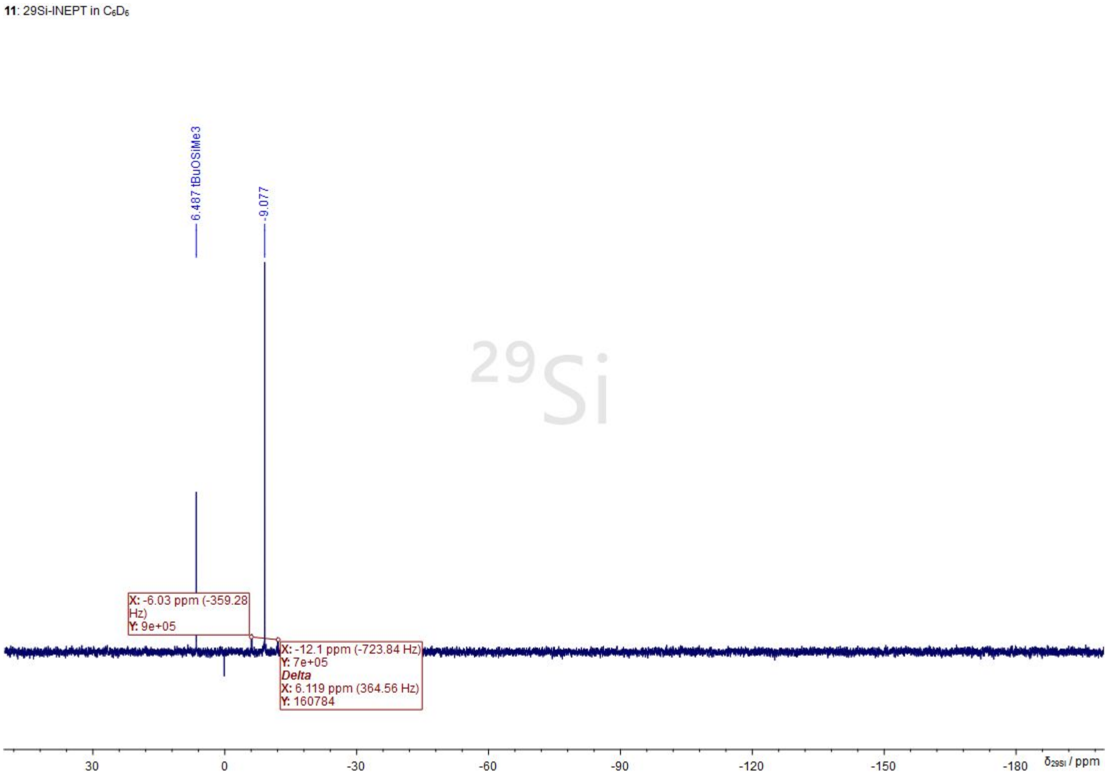

Figure S35. $^{29}Si\{H\}$-INEPT-NMR spectrum of **11** in C_6D_6

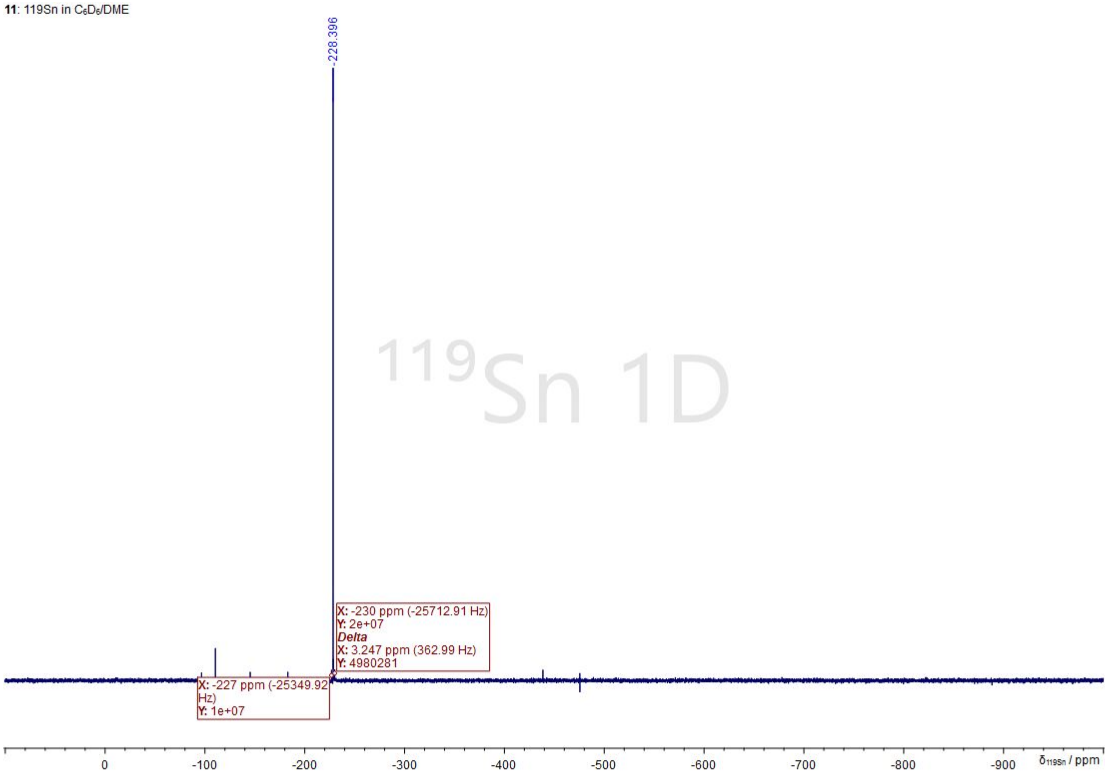

Figure S36. $^{119}Sn\{H\}$-NMR spectrum of **11** in C_6D_6/DME

Compound **12**:

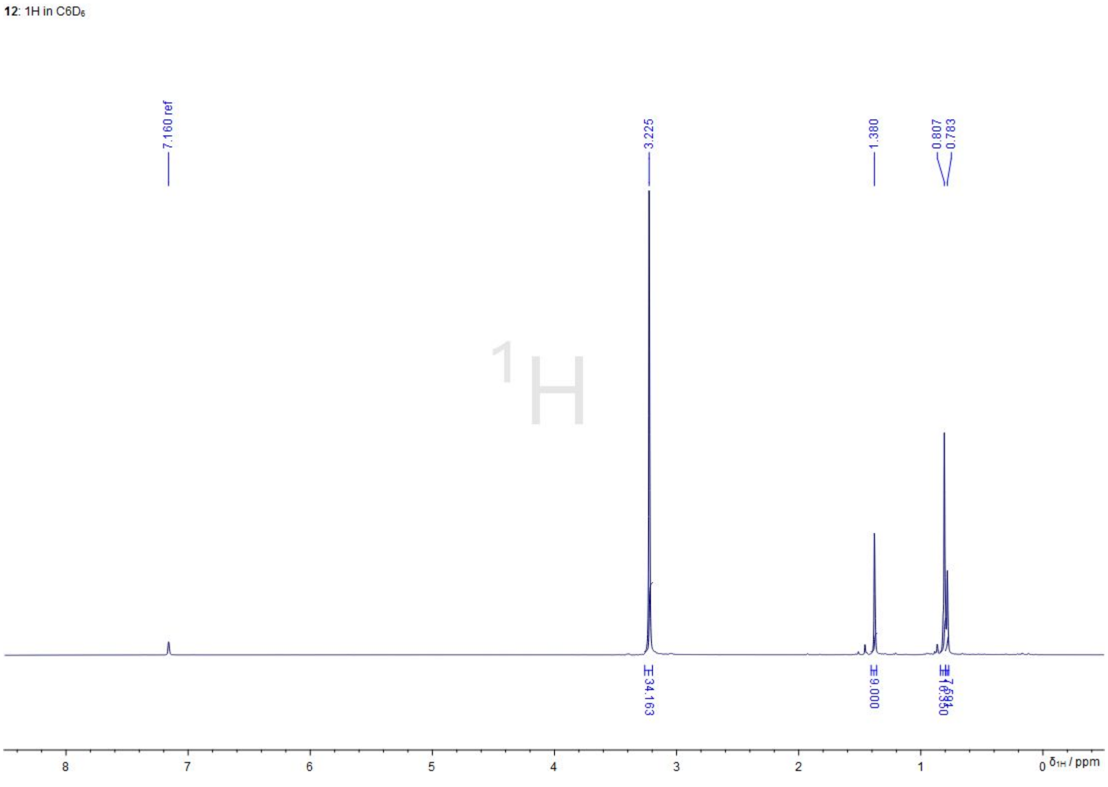

Figure S37. ^{1}H-NMR spectrum of **12** in C_6D_6

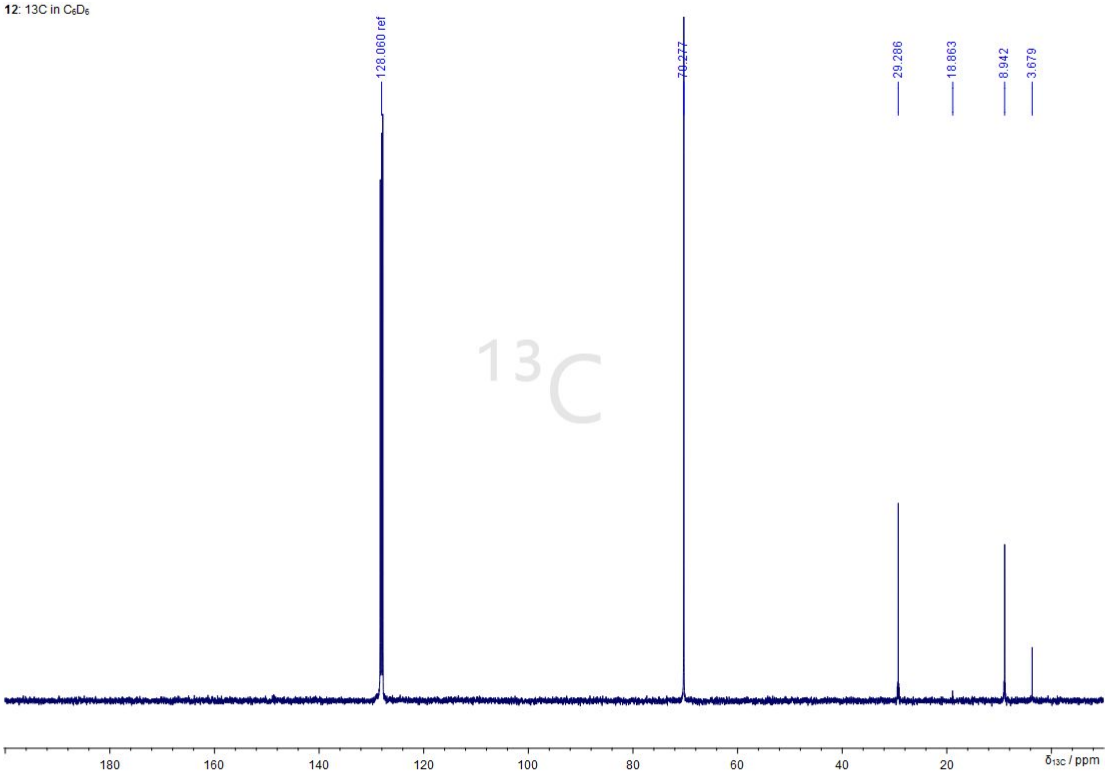

Figure S38. ^{13}C{H}-NMR spectrum of **12** in C_6D_6

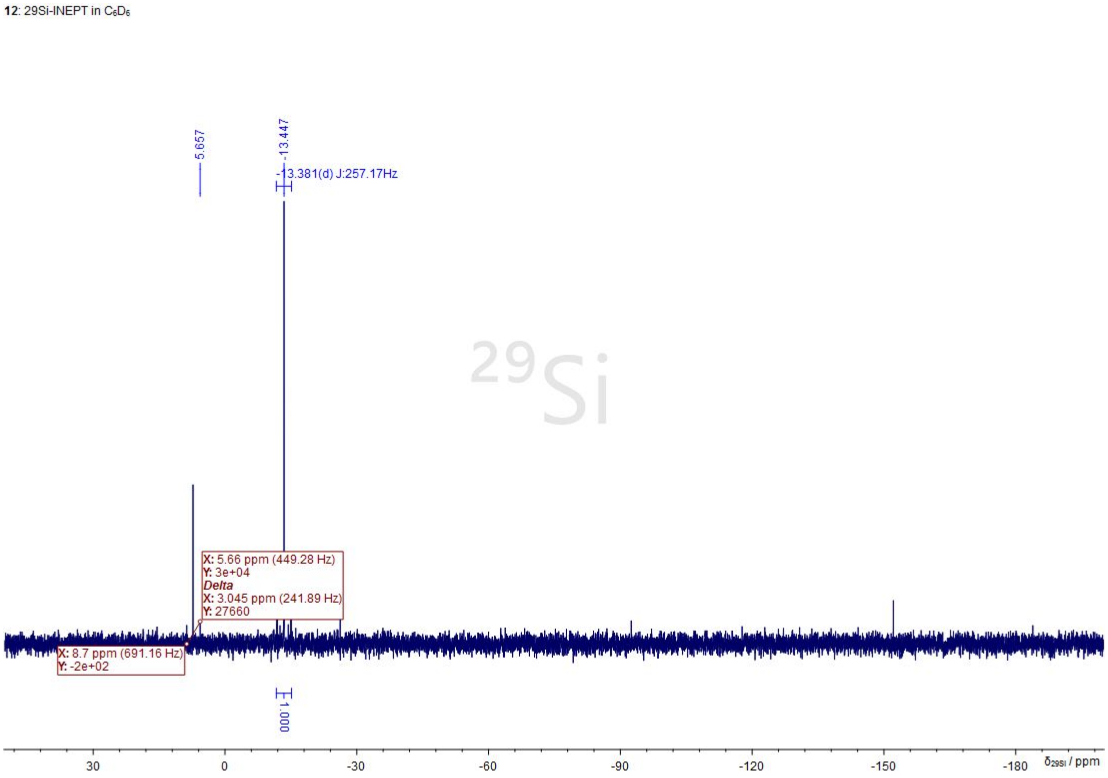

Figure S39. ^{29}Si{H}-INEPT-NMR spectrum of **12** in C_6D_6

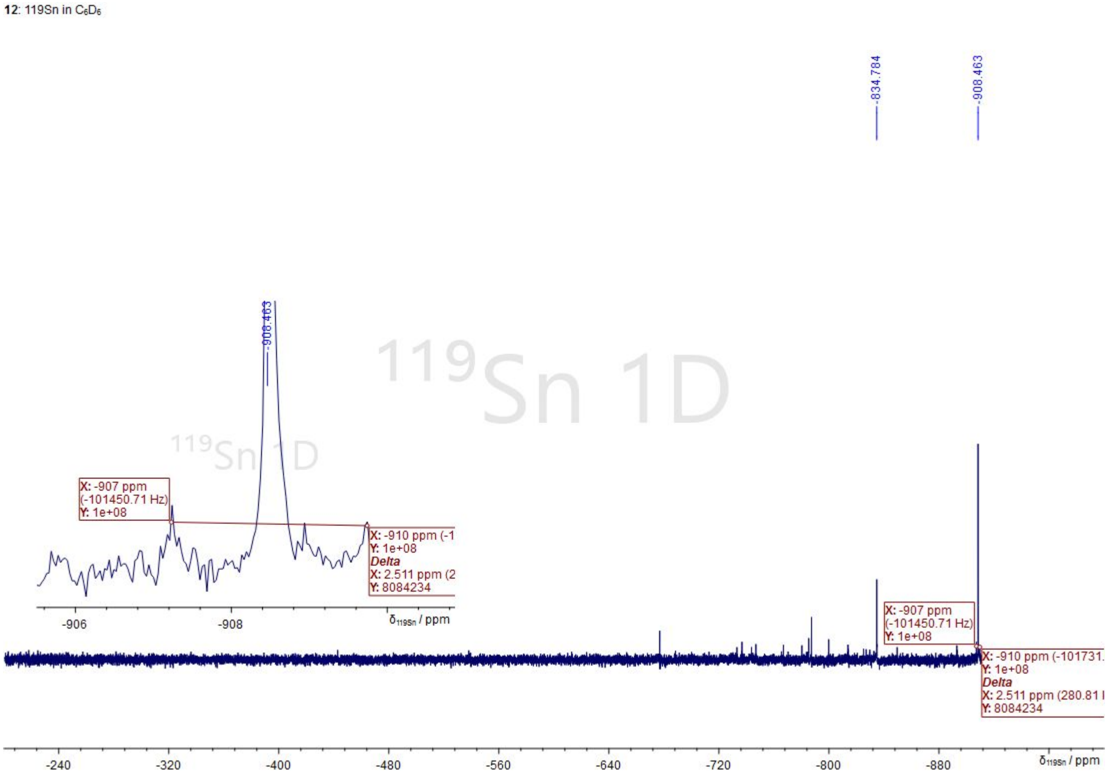

Figure S40. ^{119}Sn{H}-NMR spectrum of **12** in C_6D_6

Compound **13**:

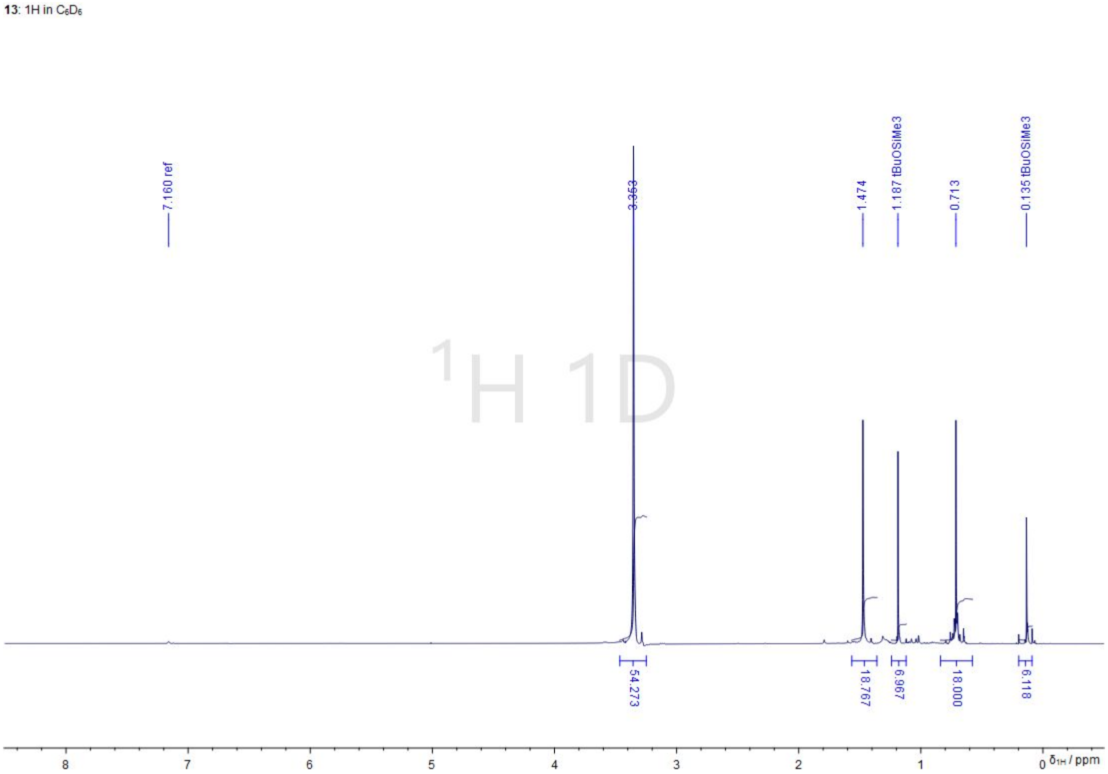

Figure S41. ^{1}H-NMR spectrum of **13** in C_6D_6

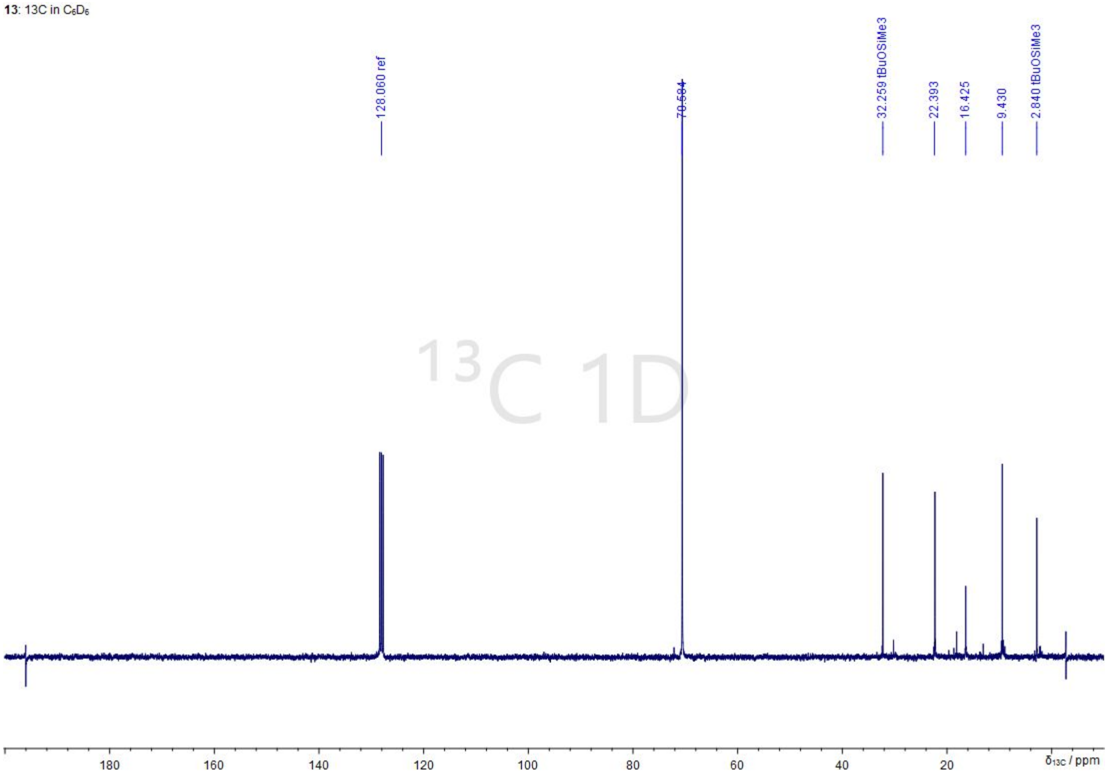

Figure S42. ^{13}C{H}-NMR spectrum of **13** in C_6D_6

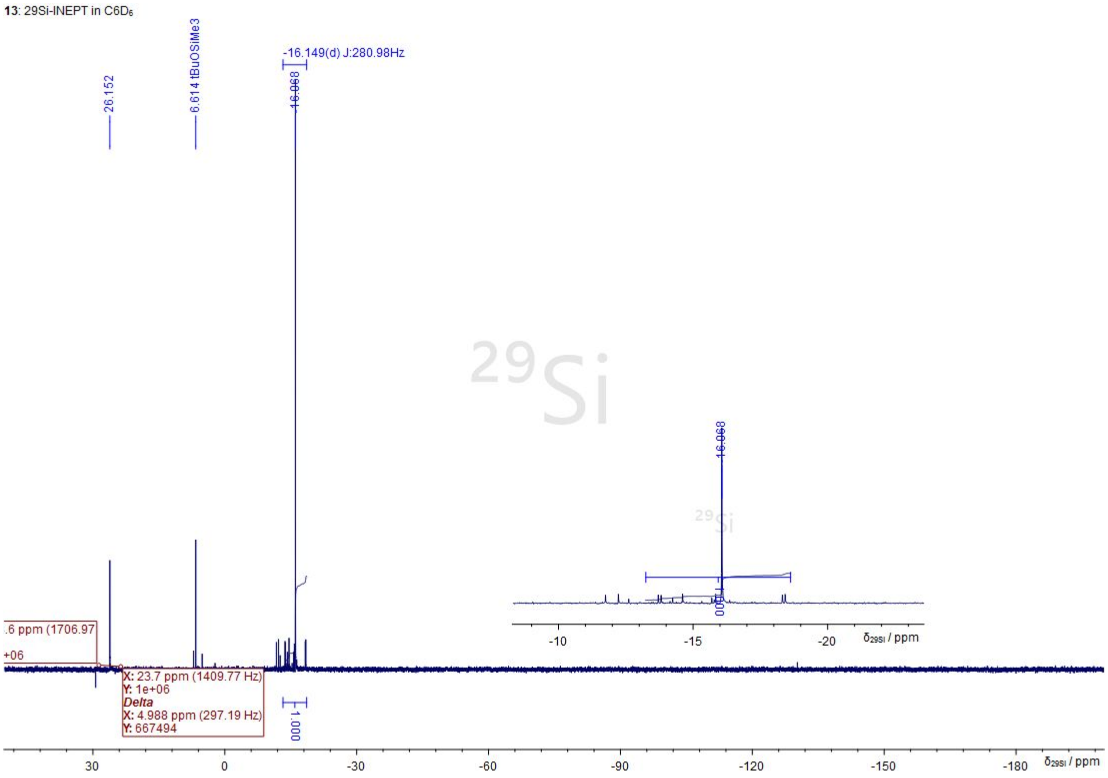

Figure S43. $^{29}Si\{H\}$-INEPT-NMR spectrum of **13** in C_6D_6

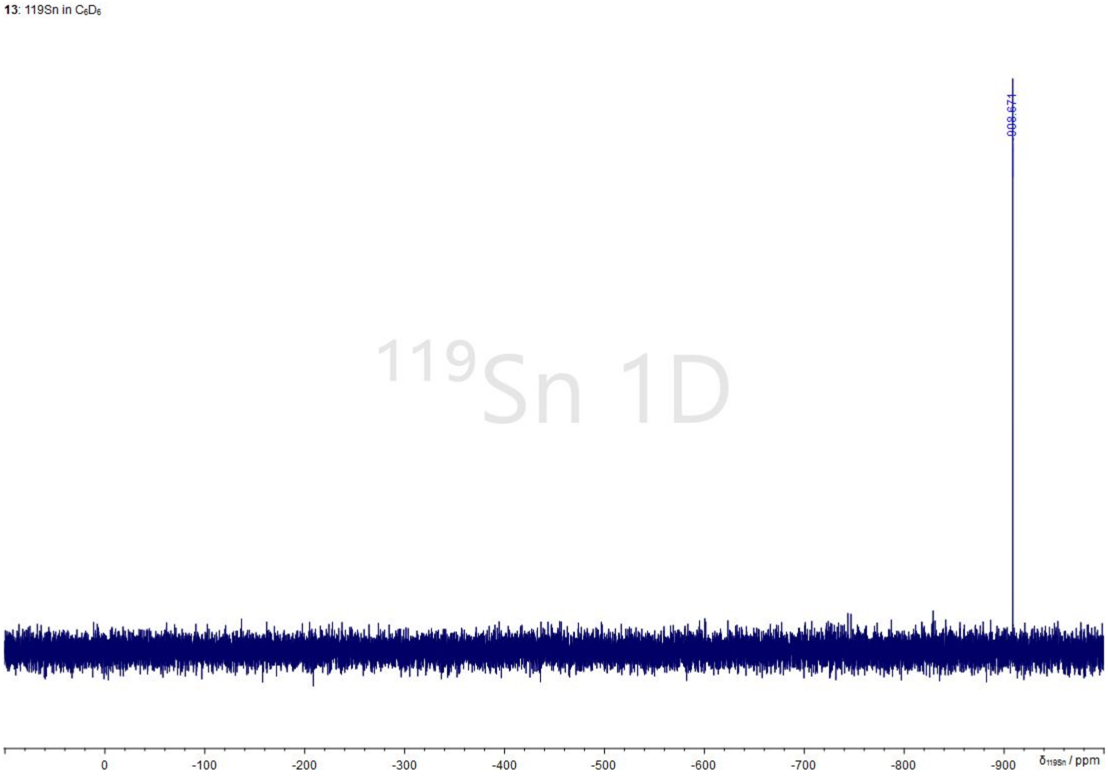

Figure S44. $^{119}Sn\{H\}$-NMR spectrum of **13** in C_6D_6

Compound **14**:

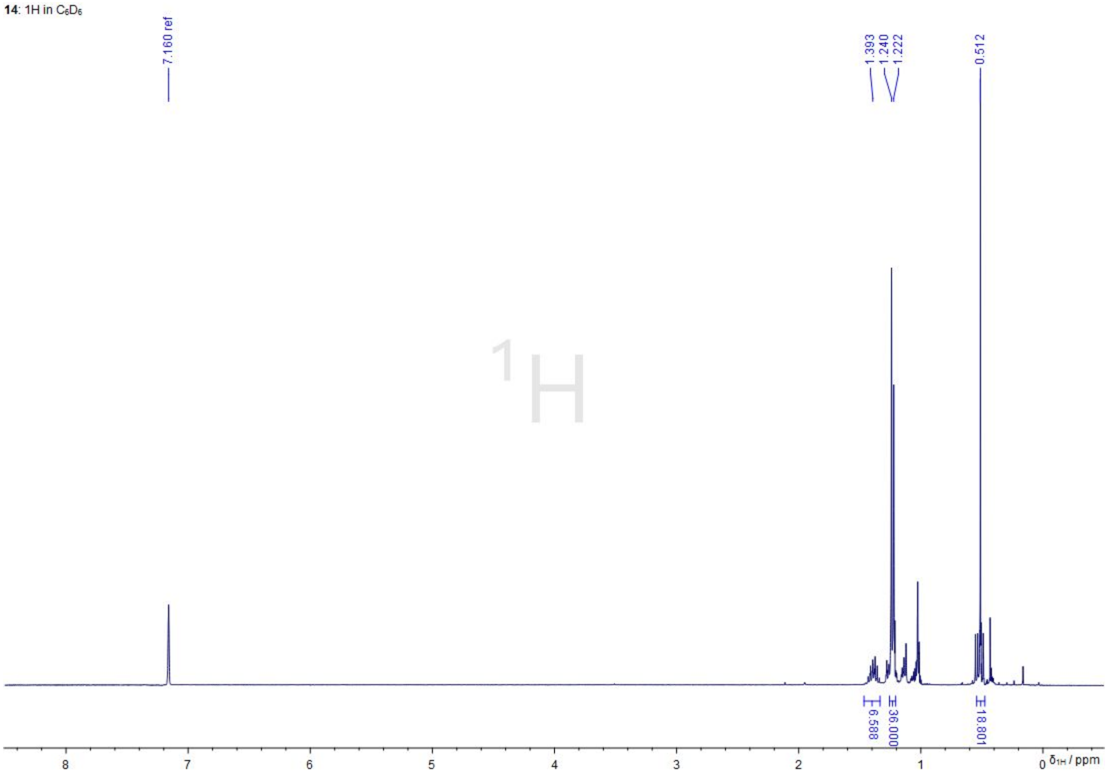

Figure S45. ^{1}H-NMR spectrum of **14** in C_6D_6

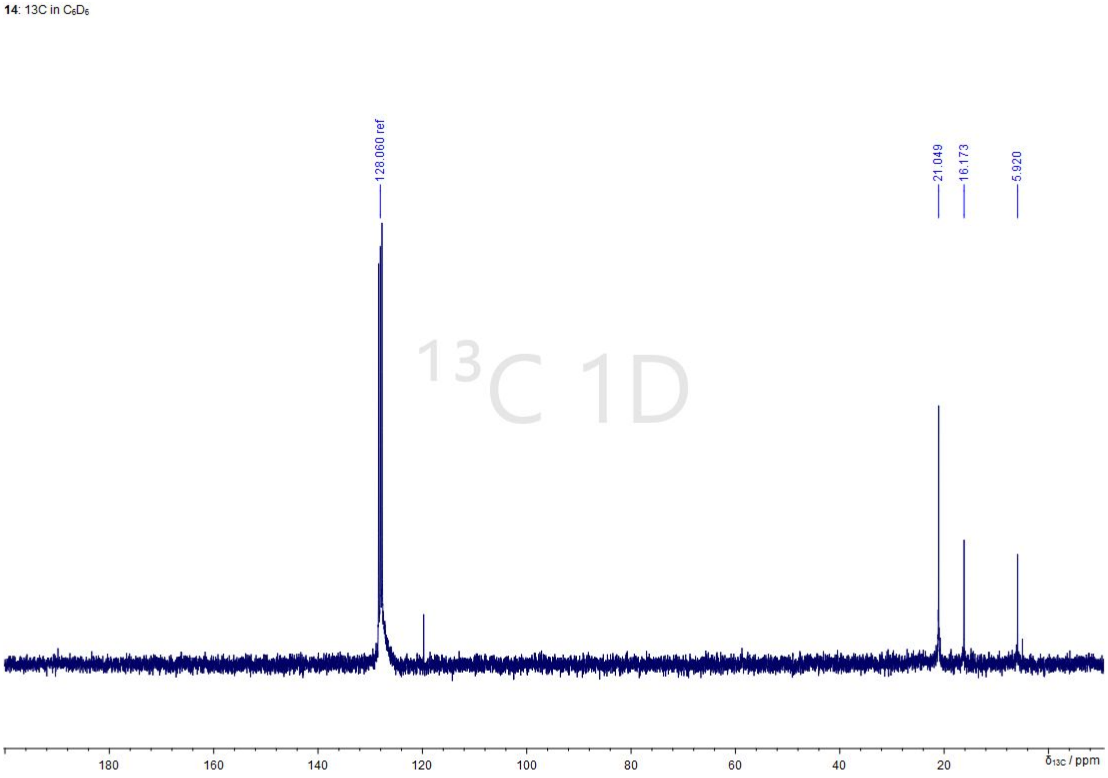

Figure S46. $^{13}C\{H\}$-NMR spectrum of **14** in C_6D_6

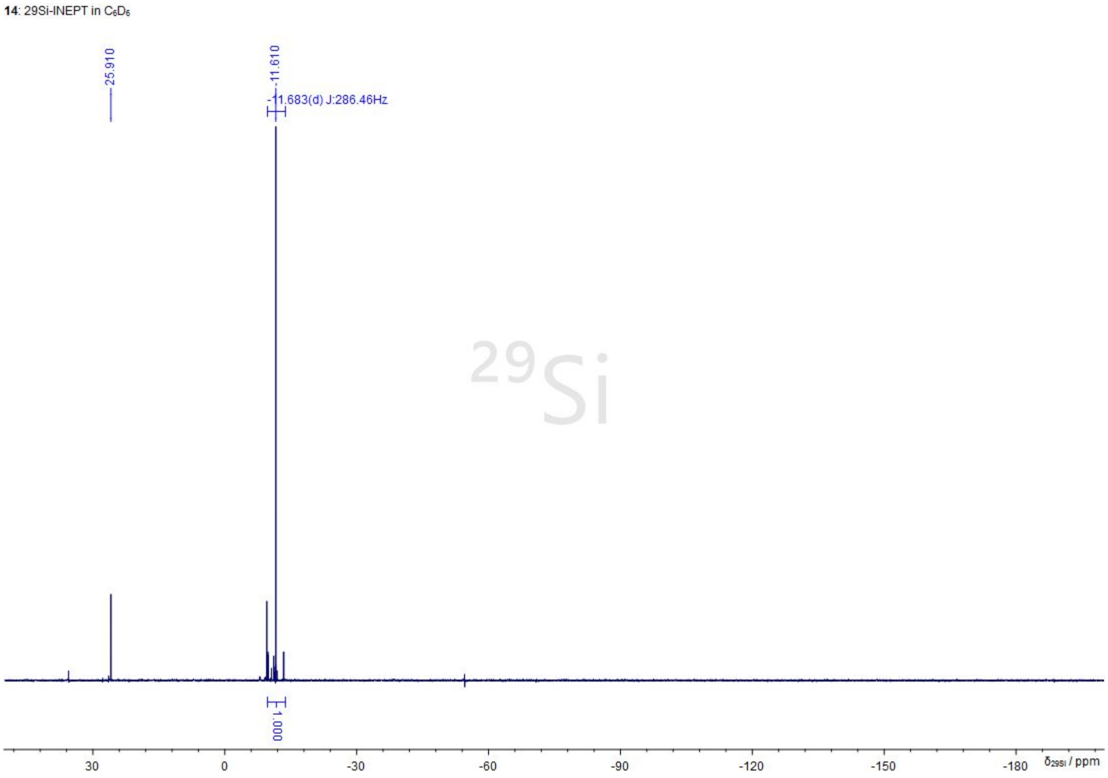

Figure S47. $^{29}Si\{H\}$-INEPT-NMR spectrum of **14** in C_6D_6

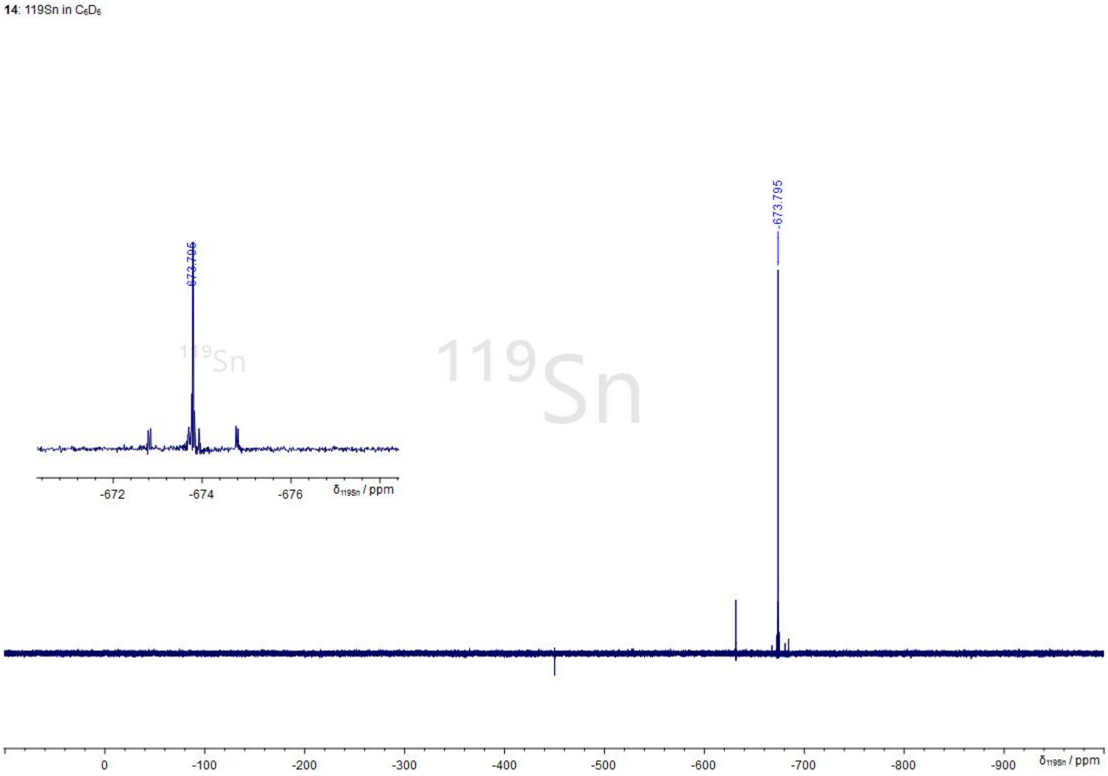

Figure S48. $^{119}Sn\{H\}$-NMR spectrum of **14** in C_6D_6

Compound **15**:

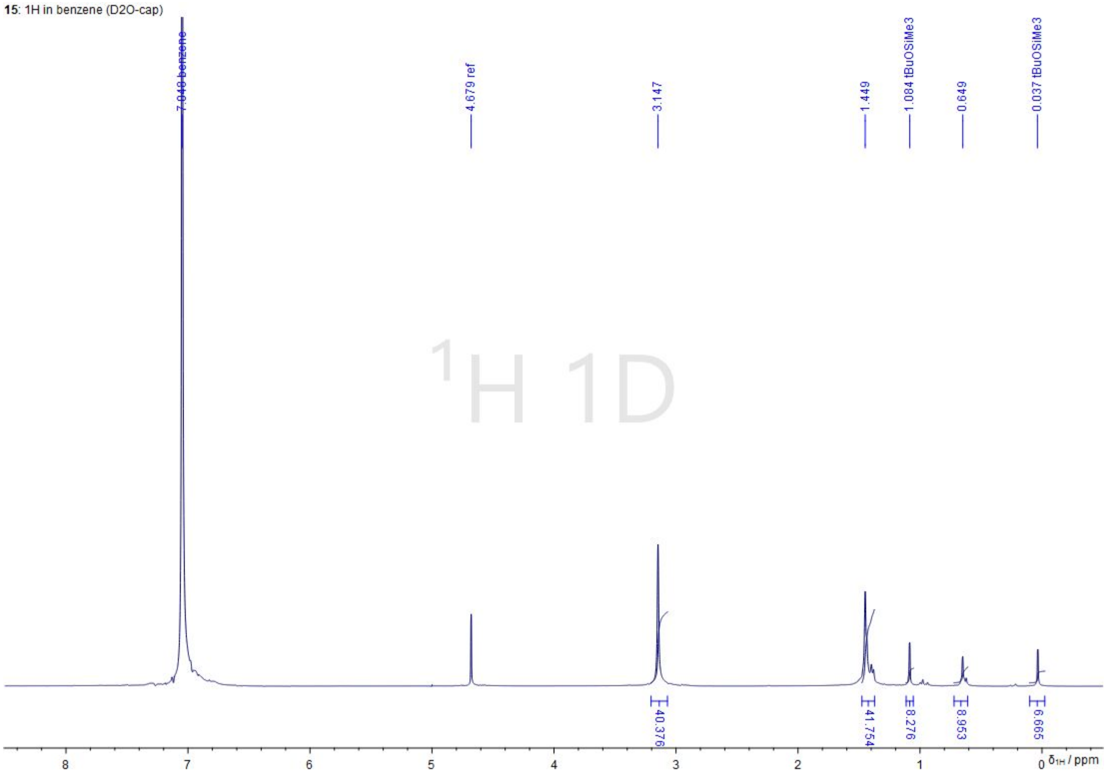

Figure S49. ^{1}H-NMR spectrum of **15** in C_6D_6

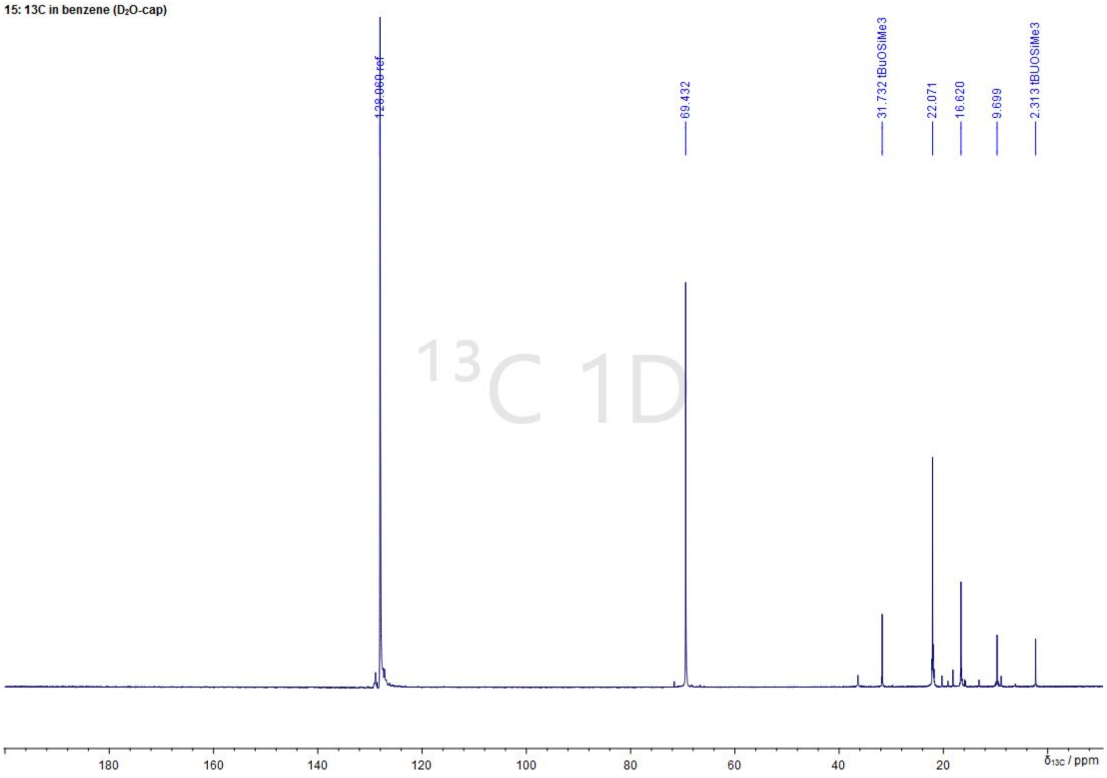

Figure S50. $^{13}C\{H\}$-NMR spectrum of **15** in C_6D_6

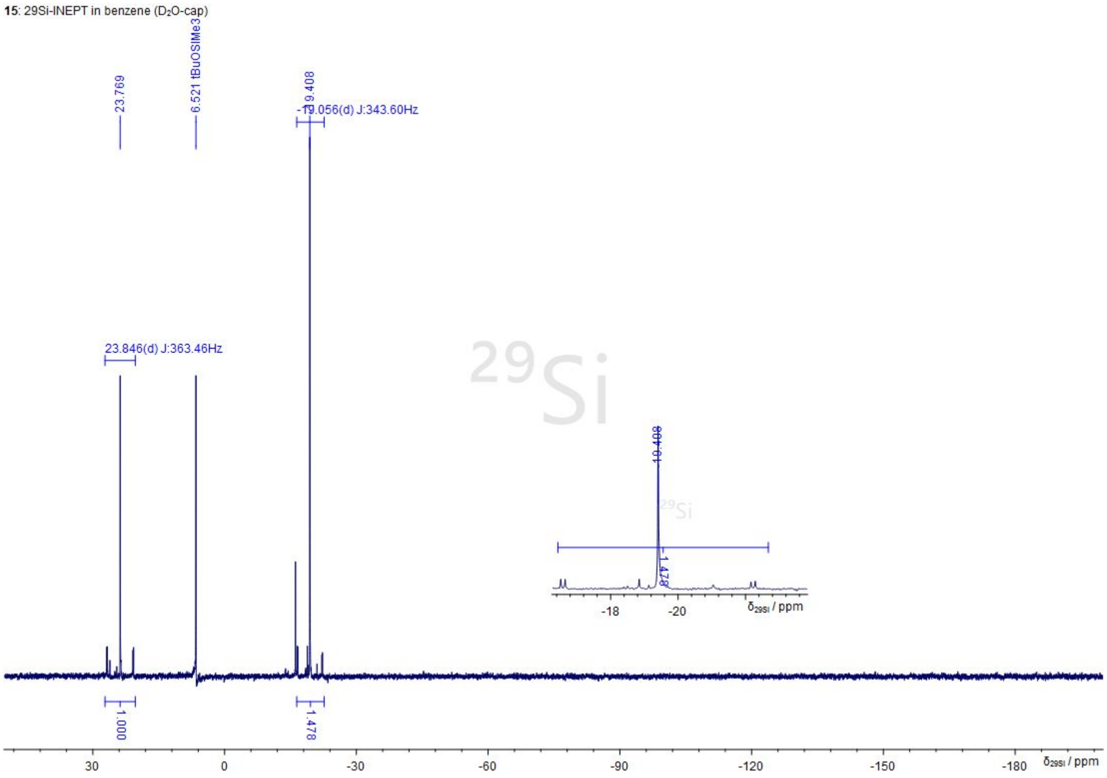

Figure S51. $^{29}Si\{H\}$-INEPT-NMR spectrum of **15** in benzen (D_2O-cap lock)

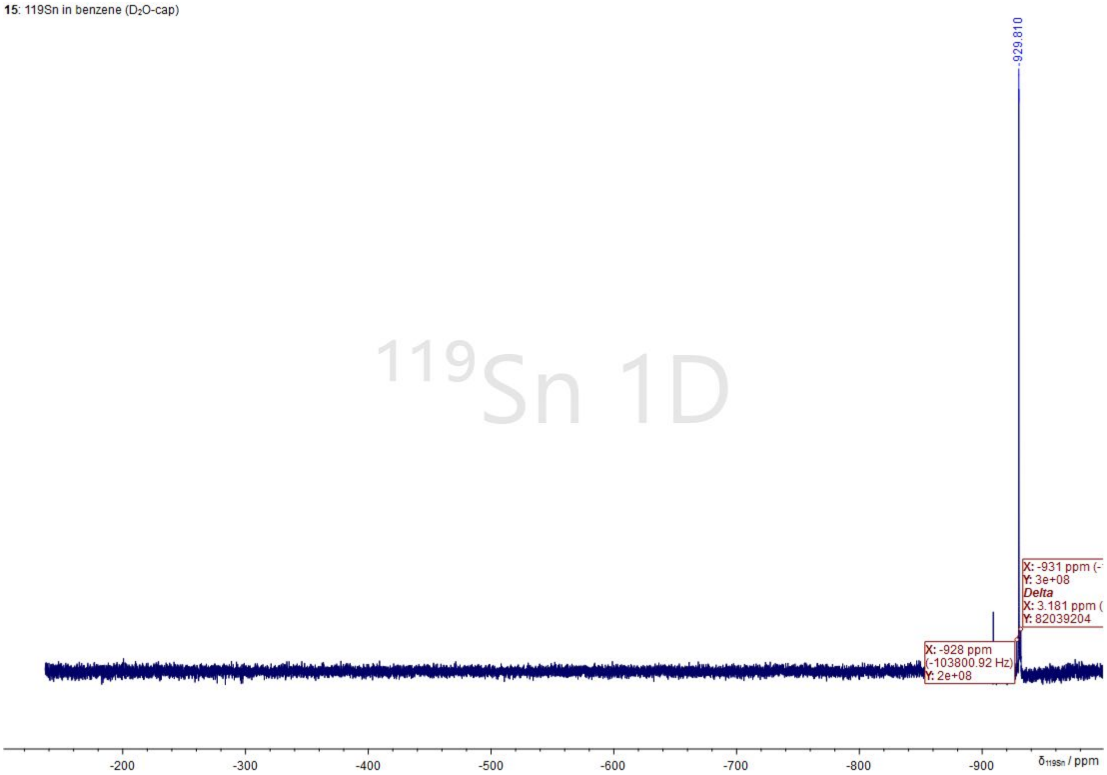

Figure S52. $^{119}Sn\{H\}$-NMR spectrum of **15** in C_6D_6

Compound **16**:

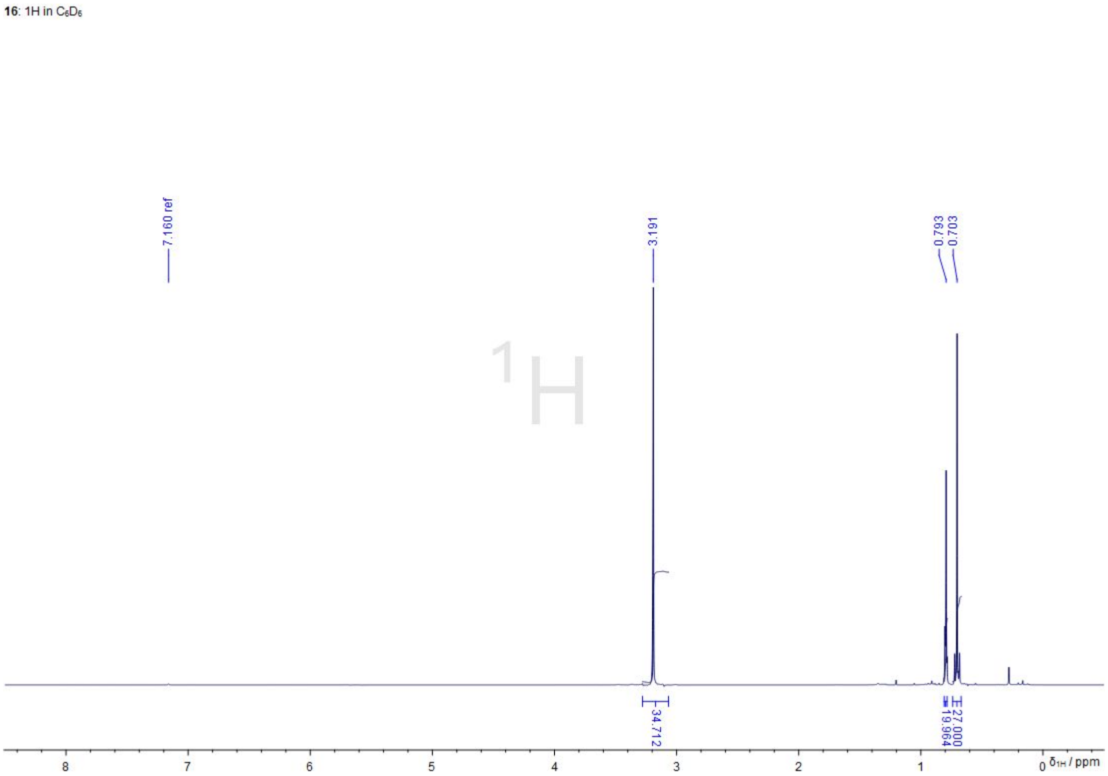

Figure S53. ^{1}H-NMR spectrum of **16** in C_6D_6

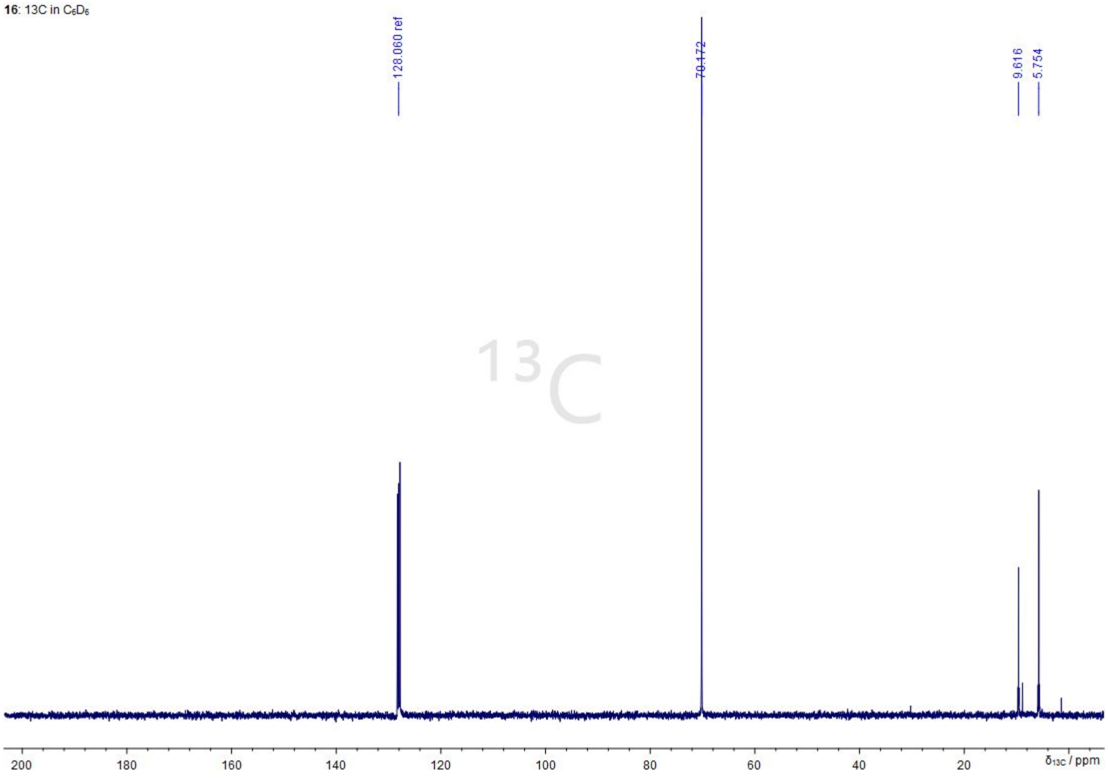

Figure S54. ^{13}C{H}-NMR spectrum of **16** in C_6D_6

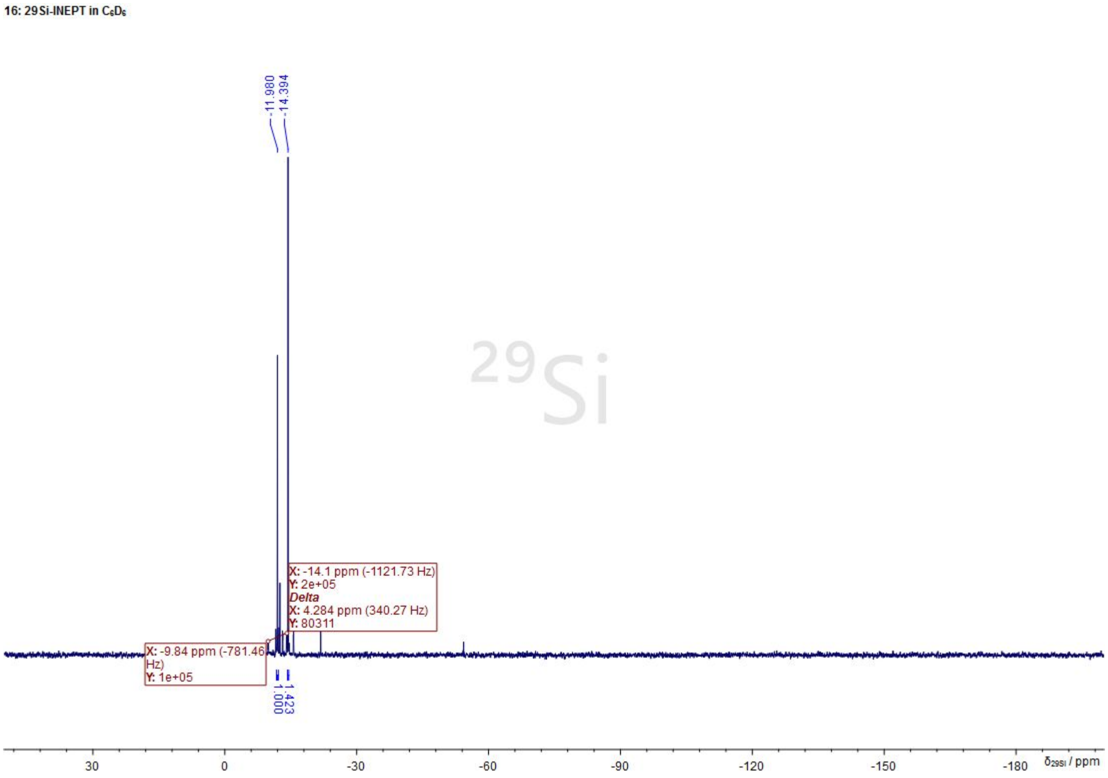

Figure S55. ^{29}Si{H}-INEPT-NMR spectrum of **16** in C_6D_6

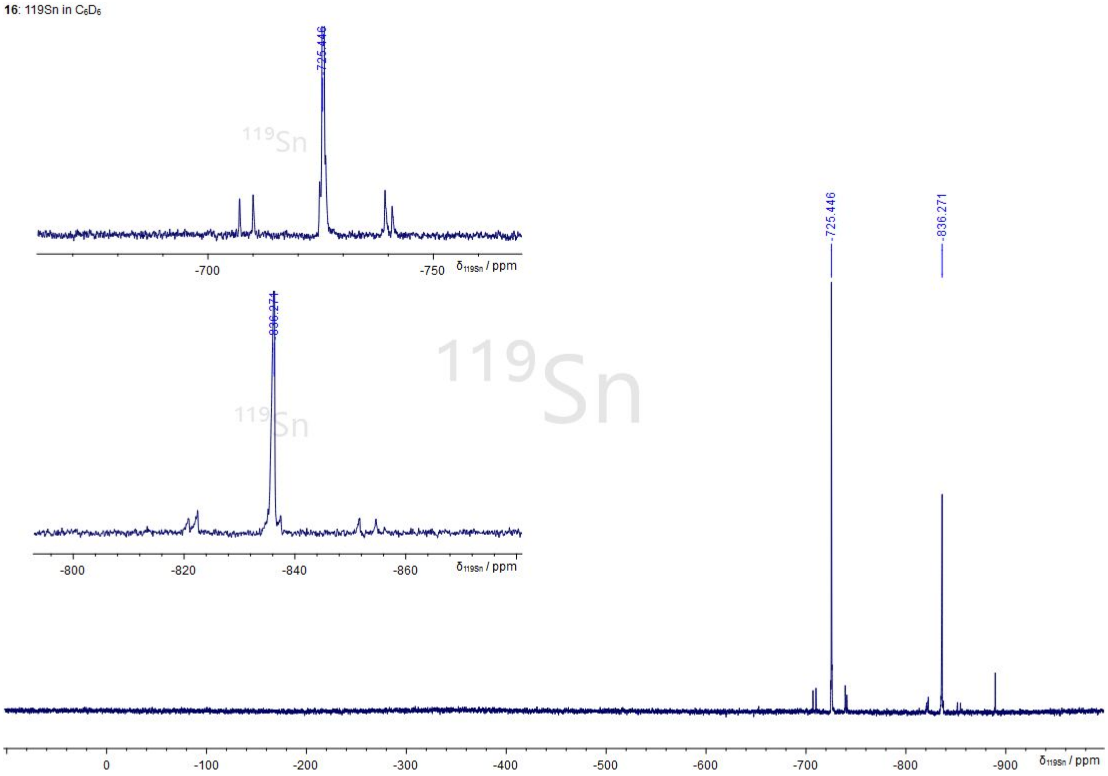

Figure S56. ^{119}Sn{H}-NMR spectrum of **16** in C_6D_6

Compound **17**:

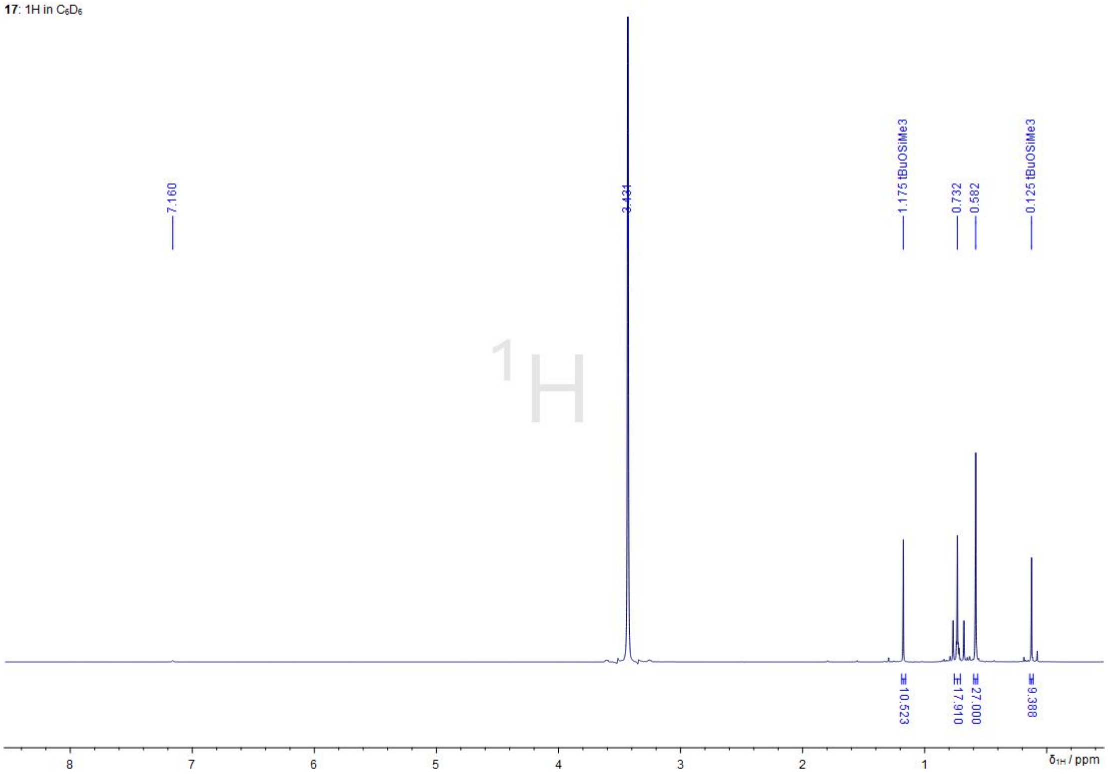

Figure S57. ^{1}H-NMR spectrum of **17** in C_6D_6

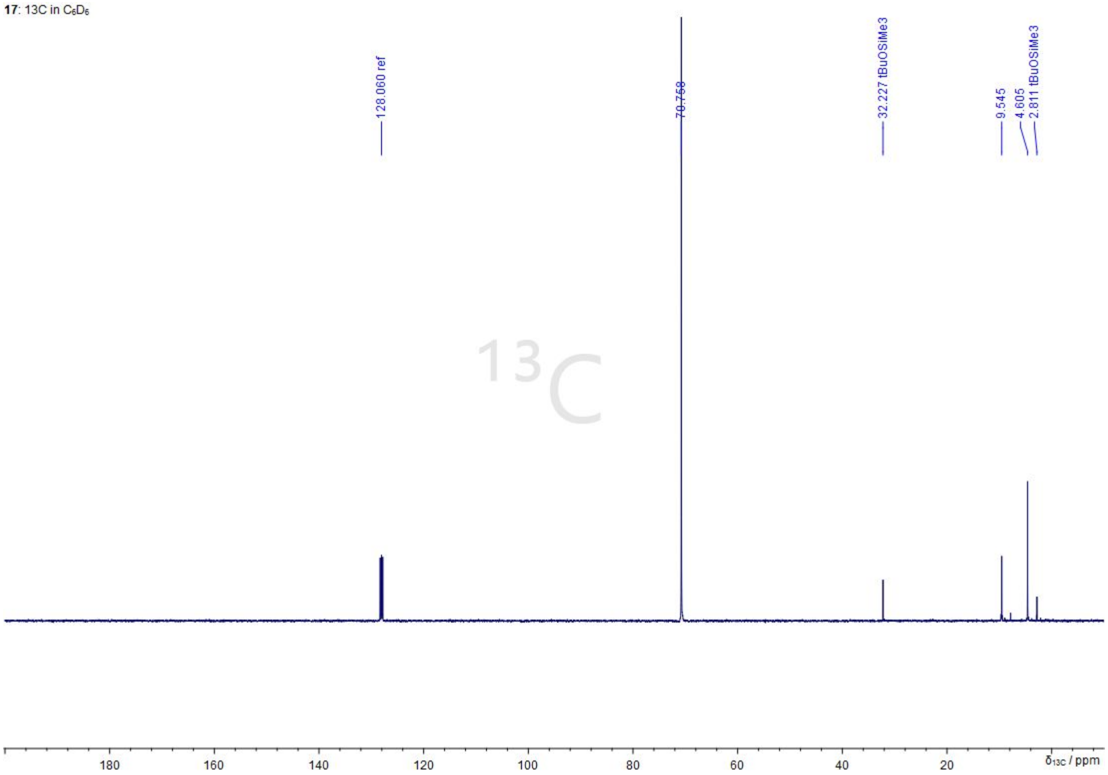

Figure S58. $^{13}C\{H\}$-NMR spectrum of **17** in C_6D_6

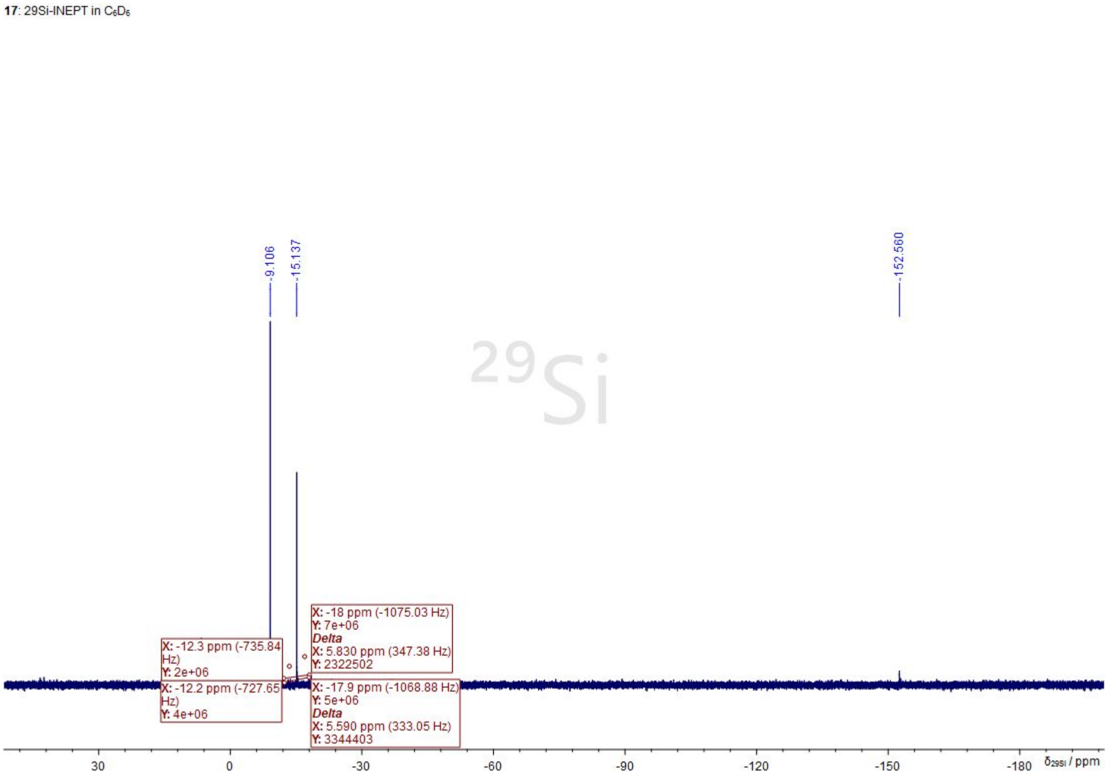

Figure S59. $^{29}Si\{H\}$-INEPT-NMR spectrum of **17** in C_6D_6

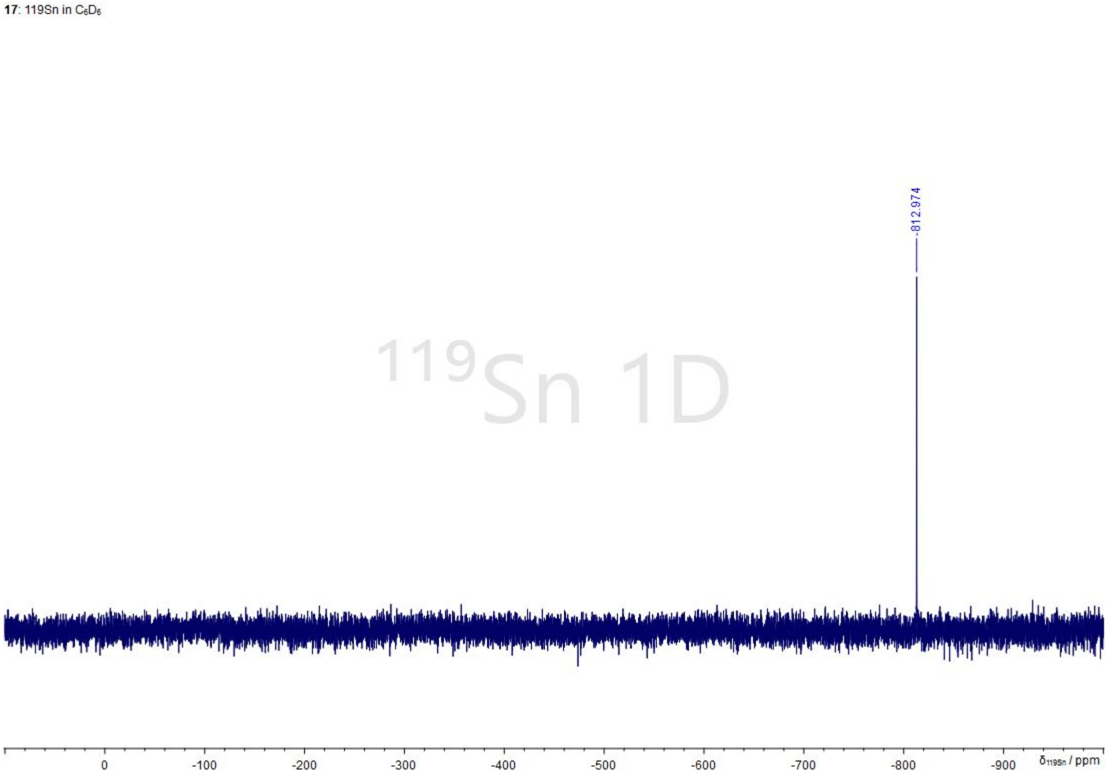

Figure S60. $^{119}Sn\{H\}$-NMR spectrum of **17** in C_6D_6

Compound **18**:

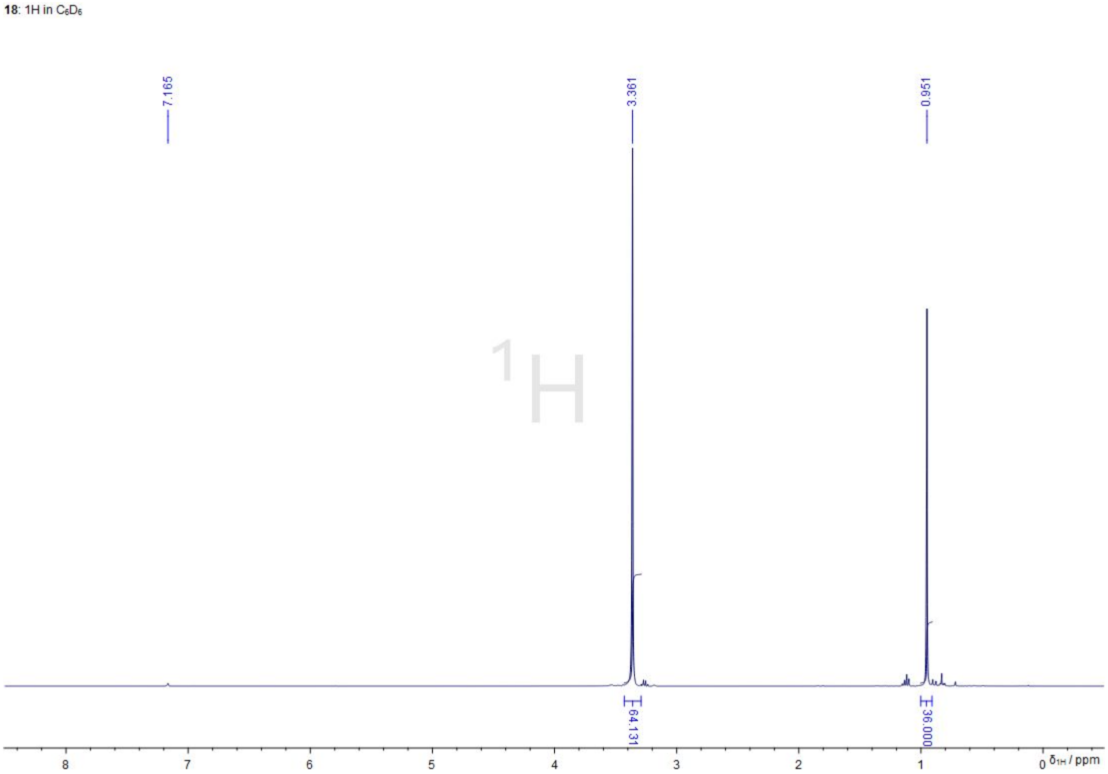

Figure S61. ^{1}H-NMR spectrum of **18** in C_6D_6

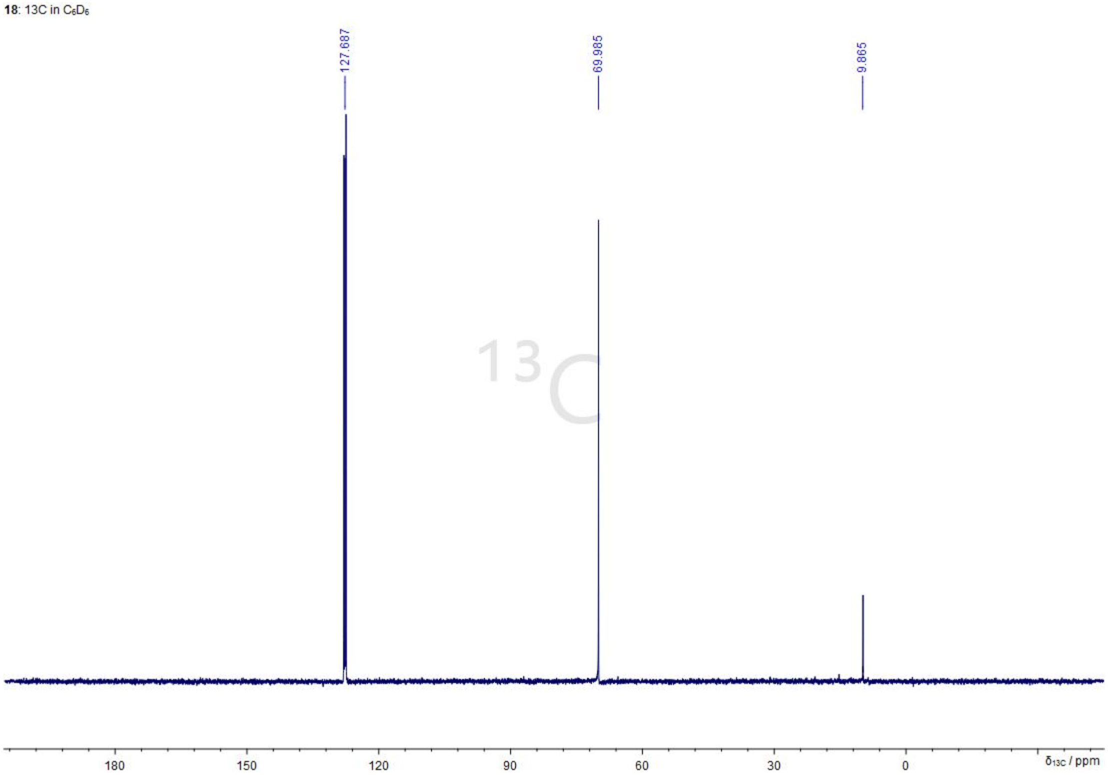

Figure S62. $^{13}C\{H\}$-NMR spectrum of **18** in C_6D_6

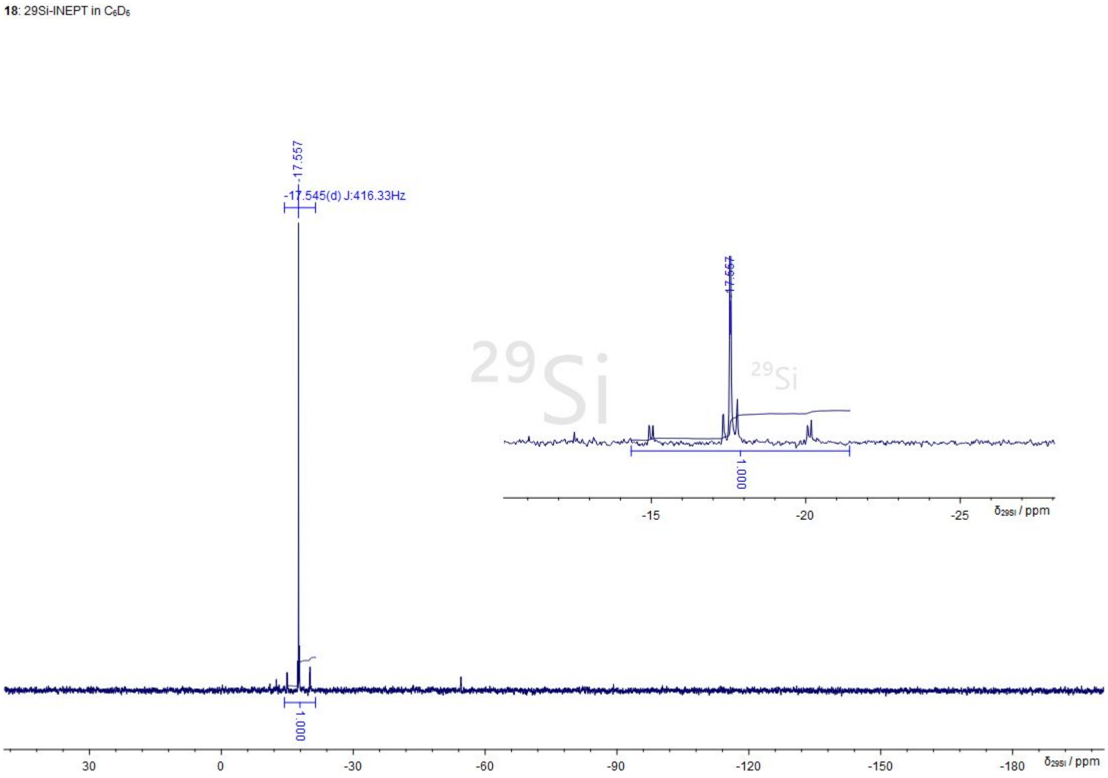

Figure S63. ^{29}Si{H}-INEPT-NMR spectrum of **18** in C_6D_6

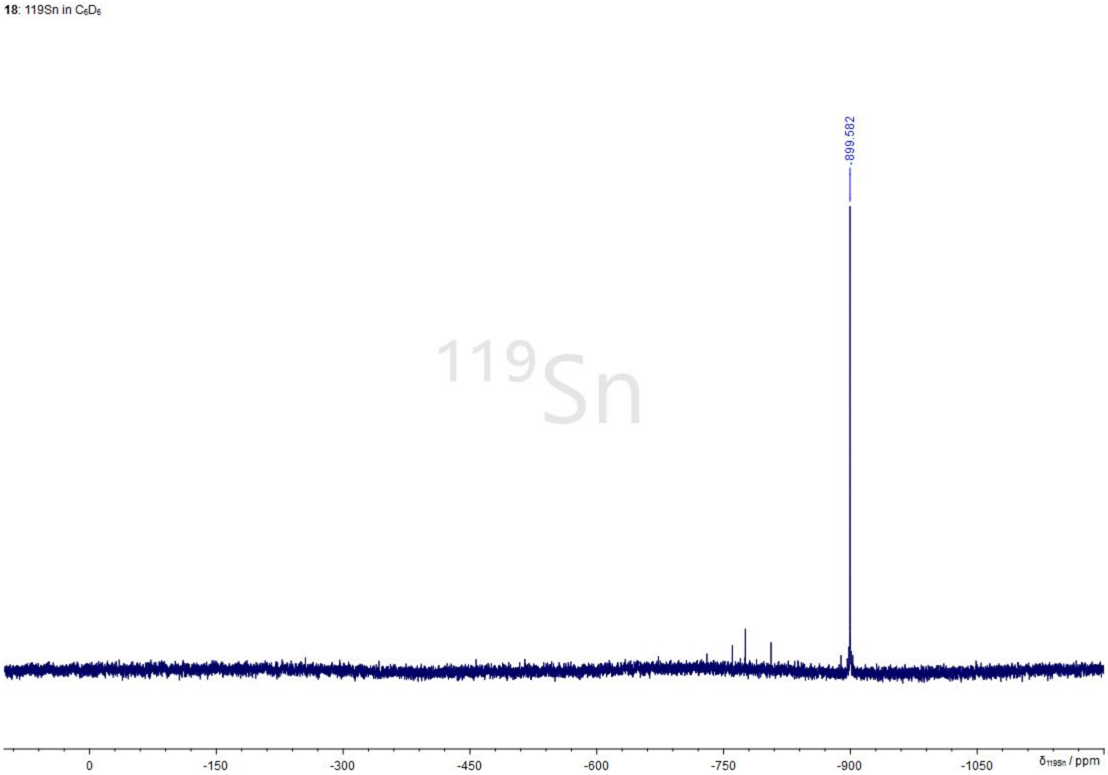

Figure S64. ^{119}Sn{H}-NMR spectrum of **18** in C_6D_6

Compound **19**:

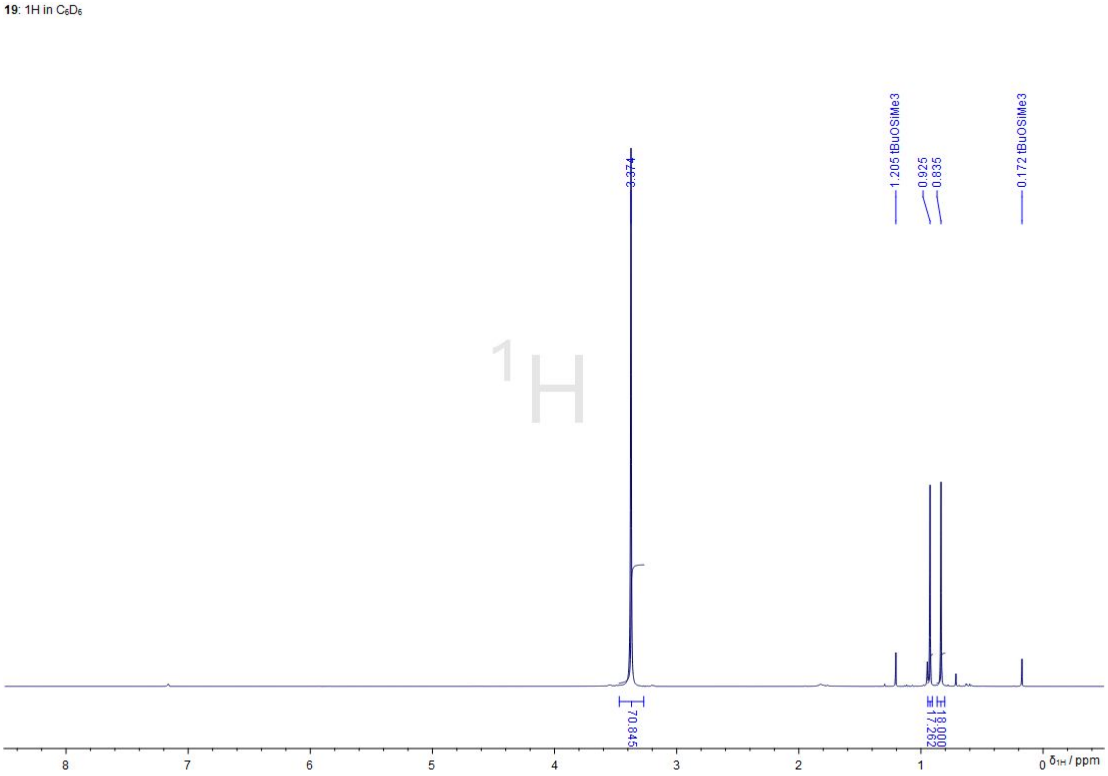

Figure S65. ^{1}H-NMR spectrum of **19** in C_6D_6

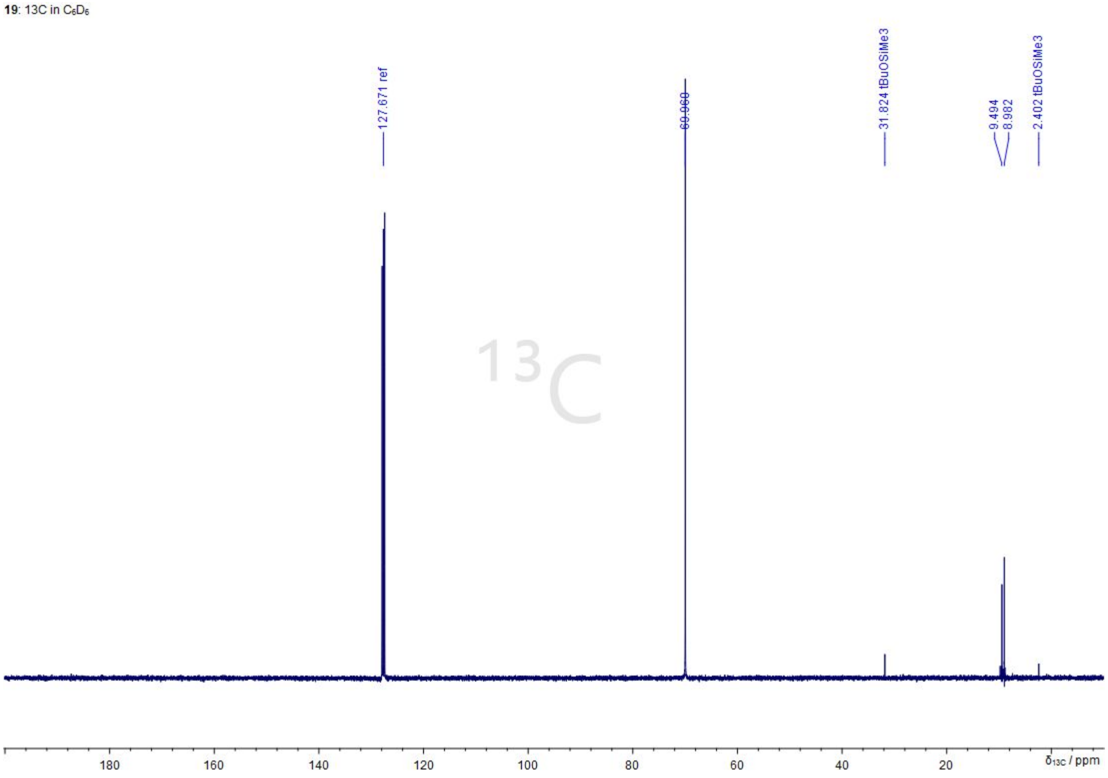

Figure S66. ^{13}C{H}-NMR spectrum of **19** in C_6D_6

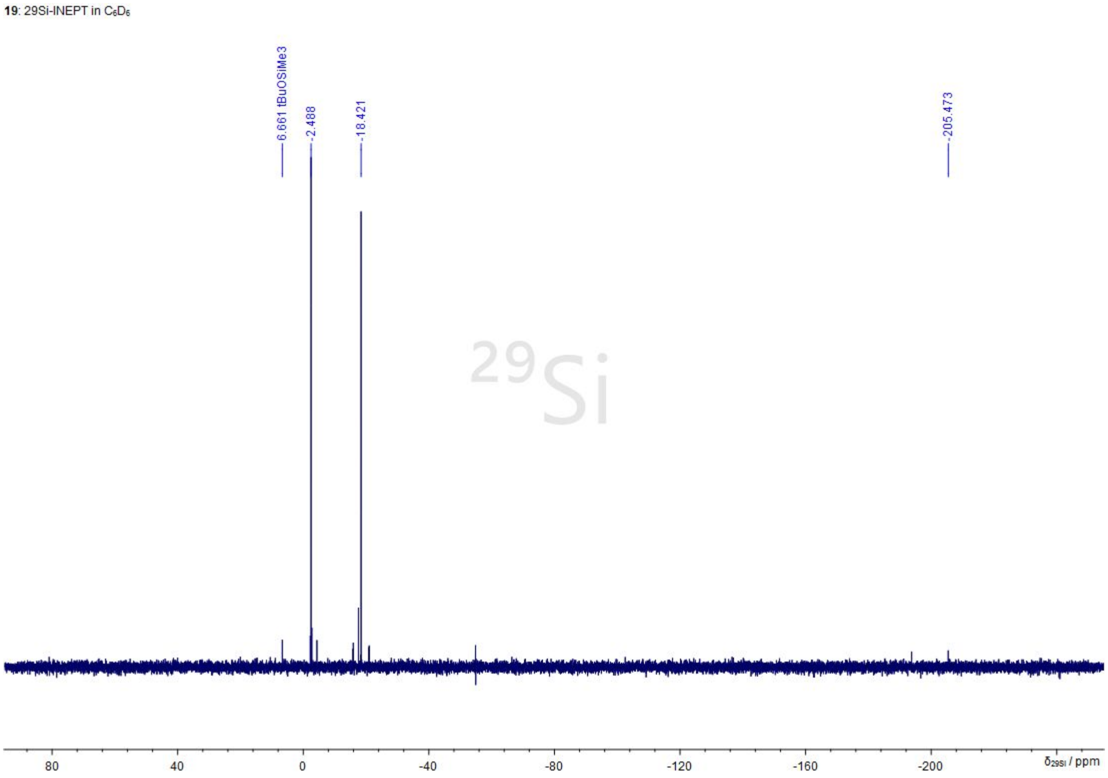

Figure S67. $^{29}Si\{H\}$-INEPT-NMR spectrum of **19** in C_6D_6

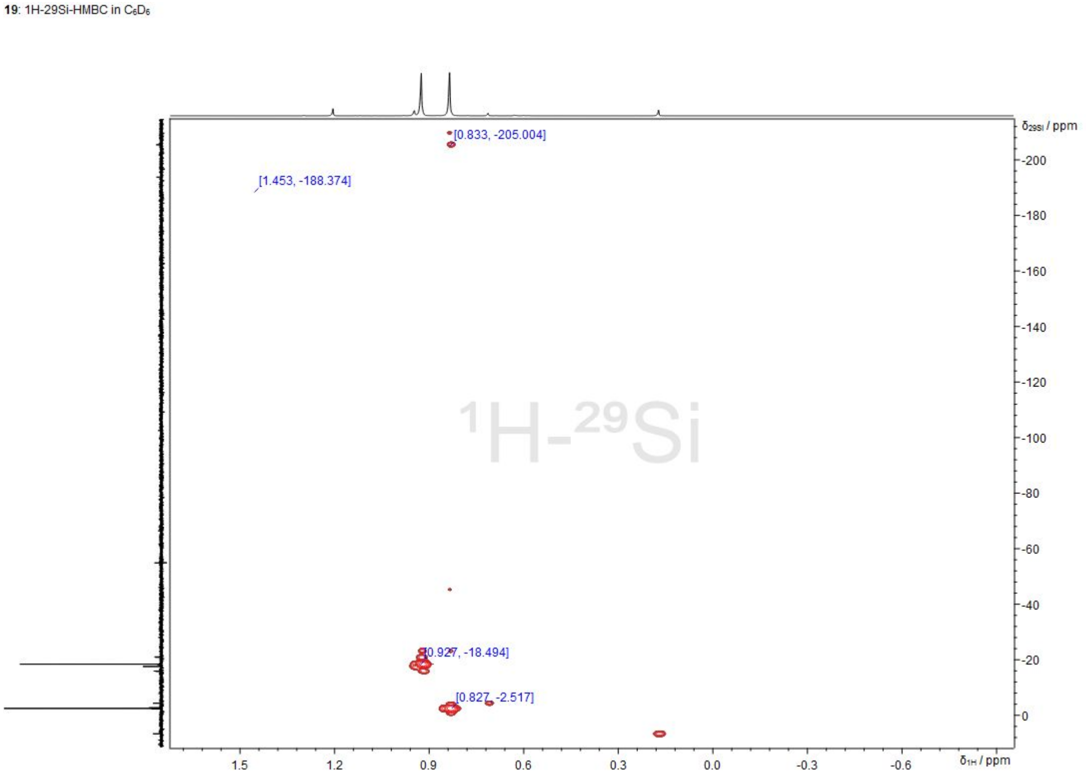

Figure S68. ^1H-^{29}Si HMBC spectrum of **19** in C_6D_6

19: 119Sn in C_6D_6

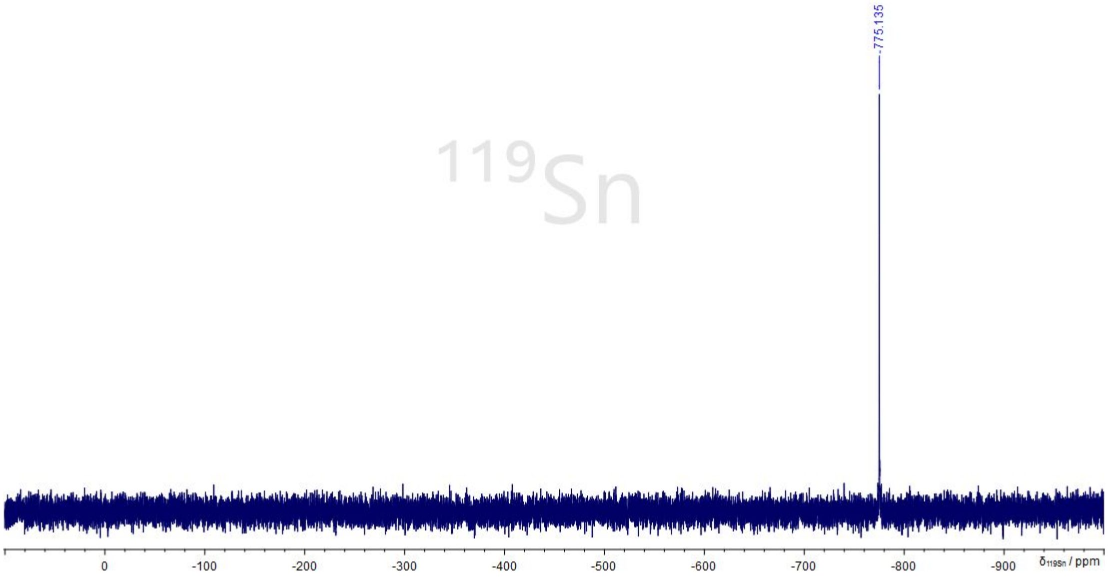

Figure S69. ^{119}Sn{H}-NMR spectrum of **19** in C_6D_6

3. Molecular structure of **19** in the solid state

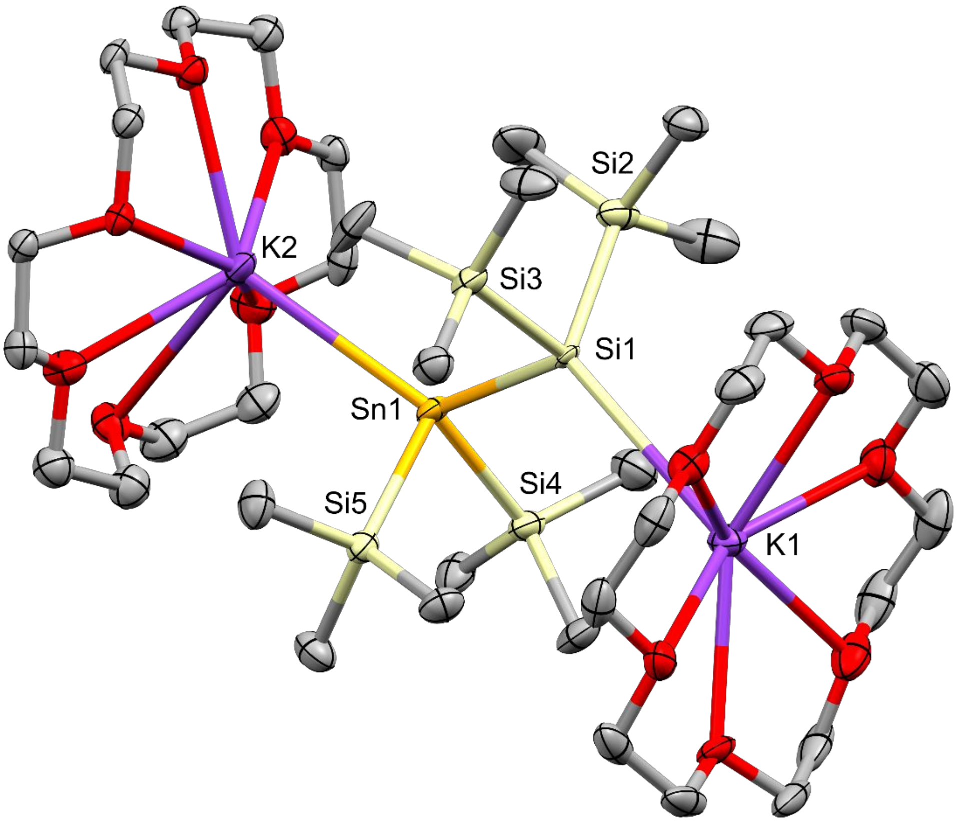

Figure S70. Molecular structure of tetrakis(trimethylsilyl)-2-siladistanna-1,2-diyl dipotassium **19** in the solid state (thermal ellipsoid plot drawn at the 50% probability level). Another half molecule of **19** on a special position, and two co-crystallized benzene molecules and all hydrogen atoms are omitted for clarity. Atom K(1) and the associated crown ether and are disordered. More seriously atoms Sn(1) and Si(1) are disordered over two positions in both molecules of **19**. A meaningful discussion of bond lengths and angles is therefore not possible.
